# Uncomplicated Clinical Malaria Features, the Efficacy of Artesunate-Amodiaquine and Their Relation with Multiplicity of Infection in the Democratic Republic of Congo

**DOI:** 10.1371/journal.pone.0157074

**Published:** 2016-06-09

**Authors:** Hypolite Muhindo Mavoko, Marion Kalabuanga, Christopher Delgado-Ratto, Vivi Maketa, Rodin Mukele, Blaise Fungula, Raquel Inocêncio da Luz, Anna Rosanas-Urgell, Pascal Lutumba, Jean-Pierre Van geertruyden

**Affiliations:** 1 Tropical Medicine Department, Faculty of Medicine, University of Kinshasa, Kinshasa, Democratic Republic of Congo; 2 Global Health Institute, University of Antwerp, Antwerp, Belgium; 3 Outpatients Department, Lisungi Health Center, Kinshasa, Democratic Republic of Congo; 4 Institute of Tropical Medicine, Antwerp, Belgium; University of California Los Angeles, UNITED STATES

## Abstract

**Background:**

In the Democratic Republic of Congo, artesunate-amodiaquine (ASAQ) is the first-line medication recommended for uncomplicated malaria treatment. We conducted a study in Kinshasa to describe the clinical features of the disease and assess the efficacy of ASAQ and its impact on the multiplicity of infection in children with uncomplicated malaria.

**Methods:**

Children aged 12 to 59 months with uncomplicated *P*. *falciparum* malaria were treated with ASAQ and followed up passively for 42 days. To distinguish new infections from recrudescent parasites, samples were genotyped using a stepwise strategy with three molecular markers (GLURP, MSP2 and MSP1). We then assessed PCR-corrected and -uncorrected day-42 cure rates and multiplicity of infection (MOI).

**Results:**

In total, 2,796 patients were screened and 865 enrolled in the study. Clinical features were characterized by history of fever (100%), coryza (59.9%) and weakness (59.4%). The crude and PCR-corrected efficacies of ASAQ were 55.3% (95%CI: 51.8–58.8) and 92.8% (95%CI: 91.0–94.6) respectively, as 83.6% (95%CI: 79.1–87.2) of the recurrences were new infections. Compared to monoclonal infections, polyclonal infections were more frequent at enrollment (88.1%) and in recurrences (80.1%; p = 0.005; OR: 1.8, 95%CI: 1.20–2.8). The median MOI at enrollment (MOI = 3.7; IQR: 0.7–6.7) decreased to 3 (IQR: 1–5) in the recurrent samples (p<0.001). Patients infected with a single haplotype on day 0 had no recrudescence; the risk of recrudescence increased by 28% with each additional haplotype (HR: 1.3, 95%CI: 1.24–1.44).

**Conclusion:**

The PCR-corrected efficacy of ASAQ at day 42 was 92.8%, but crude efficacy was relatively poor due to high reinfection rates. Treatment outcomes were positively correlated with MOI. Continued monitoring of the efficacy of ACTs—ASAQ, in this case—is paramount.

**Trial Registration:**

ClinicalTrials.gov NCT01374581

## Introduction

The Democratic Republic of Congo (DR Congo) is considered to be one of the countries most severely affected by malaria [1]. Malaria is endemic all over the country and transmission is stable in almost all areas [2]. Due to widespread chloroquine resistance, the Congolese National Malaria Control Program (NMCP) introduced sulfadoxine-pyrimethamine (SP) in 2001 as the first-line drug for uncomplicated malaria. After observing an increase in treatment failure rates with SP, the NMCP turned to artemisinin-based combination therapy (ACT) in 2005, in particular artesunate-amodiaquine (ASAQ), to replace SP. Artemisinin derivatives are known to act quickly and have a short half-life, and they are now being combined with a longer-acting partner drug which also plays a prophylactic role [3]. The synergistic action of the two drugs is expected to halt the emergence of resistance [4]. In 2010, the NMCP selected artemether-lumefantrine (AL) as an alternative first-line treatment, alongside ASAQ [5]. However, the availability of AL remains limited in the public sector [6]. Oral quinine was to be used as a rescue treatment in combination with an antibiotic that has antiplasmodial properties (tetracycline, doxycycline or clindamycine), according to the latest guidelines [5].

Monitoring the efficacy of ACTs is essential, especially since the reported emergence of resistance to artemisinin derivatives in Southeast Asia [3,7,8]. The latter constitutes a threat to recent gains in malaria control efforts, especially in Sub-Saharan Africa, where the majority of malaria cases occur. Fortunately, K13 mutations associated with artemisinin resistance have not yet been identified in Africa [8–10]. Since the implementation of ACT in DR Congo, most studies have reported ASAQ efficacy to be above the required threshold of 90% [6,11,12]. In one study conducted 10 years ago, PCR-corrected efficacy at day 28 was reported to be 85% in the Equator province [11]. However, the loose dose of ASAQ used in that study has a lower efficacy than the fixed dose [13].

This study describes malaria features in Kinshasa and assesses the efficacy of ASAQ after 42 days of treatment. In addition, it presents a cohort in which children who experienced clinical failures became eligible for inclusion in a randomized clinical trial (RCT) to assess the efficacy and safety of three rescue treatments, known as the Quinact study [14]. Furthermore, understanding of the diversity of malaria strains circulating and those involved in an infection episode can impact both treatment outcomes and the development of immunity in a community [15].

## Materials and Methods

### Trial design

This is a report on the first phase of the Quinact study in DR Congo, a bi-center, randomized, open label, 3-arm trial performed in three phases [14]. In DR Congo, the first phase consisted in treating eligible patients with ASAQ, the recommended drug for first-line treatment of uncomplicated malaria at the time the study was conceived. It was conducted at Lisungi Health Center in Kinshasa, the capital city of DR Congo.

### Patients and treatment

We screened boys and girls aged between 12 and 59 months attending Lisungi Health Center for fever (tympanic temperature ≥38.0°C) or history of fever. Enrollment in the study was based on the following criteria: body weight ≥9 kg; microscopically confirmed mono-infection or mixed infection containing *Plasmodium falciparum* (parasitemia ≥2,000/μl to 200,000/μl); hemoglobin (Hb) value ≥6.0 g/dl; signed (or thumb-printed and witnessed by an impartial witness if parents/guardians were illiterate) informed consent granted by the parents or guardians and parents’ or guardians’ willingness and ability to comply with the study protocol for the duration of the study. The body weight threshold of 9 kg was selected because the pre-qualified quinine used in the randomized phase was not available for children below this weight.

We excluded patients with severe malaria [16] or danger signs (not able to drink or breastfeed, vomiting more than twice in 24 hours, recent history of more than one convulsion in 24 hours, unconscious state, unable to sit or stand), known hypersensitivity and previous serious adverse events related to the study drugs, intercurrent illness, severe malnutrition, treatment with drugs which may prolong the QT interval (imidazole and triazole, antifungal agents), ongoing prophylaxis with drugs demonstrating antimalarial activity and those who had participated in any other investigational drug study during the previous 30 days.

ASAQ (Artesunate-amodiaquine Winthrop, Sanofi) was administered once daily over the course of three days. The tablets contained 50 mg of artesunate and 135 mg of amodiaquine for patients weighing 9 to 17.9 kg; and 100 mg of artesunate and 270 mg of amodiaquine for those weighing 18 to 35.9 kg. Treatment was observed directly by a study nurse. Patients were required to remain at the clinic for at least 60 minutes after drug intake. A full or half dose was repeated if vomiting occurred within 30 minutes or between 30 and 60 minutes of drug administration, respectively. If vomiting persisted, the patient was withdrawn from the study and referred to the health facility. The TREND checklist and the study protocol are available; see S1 TREND Checklist, S1 Protocol, and S2 Protocol.

### Follow up

After enrollment, patients were asked to attend the study site on the two following days for directly observed treatment in order to complete the 3-day course of ASAQ and then every two weeks until day 42. They were encouraged to attend the study site on any unscheduled day if they experienced any health problems. Adverse events (AEs) were documented at each visit. Follow-up was passive. Malaria infection was not screened systematically; this decision was made for logistical reasons, because the study team planned to implement standard active follow-up for the two subsequent phases [14]. Blood smears were only performed on day 42 and when patients were clinically suspected of having malaria during follow-up.

Blood samples were collected on filter paper (Whatman 3MM) on day 0 and on days when blood smears were performed, to enable subsequent parasite genotyping. Serum samples were also collected upon recruitment and frozen for further immunological assessment (to be reported elsewhere).

### Clinical assessment

Symptoms and physical examination findings were graded according to their severity (mild, moderate or severe) using the severity grading scale developed by the World Health Organization and the United States National Institute of Health, Division of Microbiology and Infectious Diseases.

### Laboratory procedures and molecular interpretation

Thick and thin blood films were prepared, dried and stained with Giemsa 10% for 10 minutes. Thin smears were fixed with methanol before staining. Slides were examined using a light microscope at 1000 times magnification. The density of asexual parasites was determined on the basis of the number of parasites per 200 white blood cells (WBC), assuming a total WBC count of 8,000/μl. If fewer than 10 parasites were read per 200 WBC, the count was extended to 500 WBC. An independent double reading was performed, with the average taken as the final result. A third reader was required in the event of discrepancies. Hb was measured using a portable spectrophotometer (Hemocontrol, EKF Diagnostics, Barleben, Germany). The data management team based at the Infectious Diseases Institute, Makerere University in Uganda, produced a list of random slides to be sent periodically to the Institute of Tropical Medicine (ITM) in Antwerp, Belgium, for external quality control. In addition, two laboratory-specific monitoring visits were conducted by a lab technician from ITM’s malaria unit.

PCR genotyping was performed at ITM, Antwerp, Belgium, to distinguish recrudescence from reinfection. DNA was extracted from dried blood spots using the QIAGEN QIAamp96 DNA blood kit. Each punch (5 mm) was eluted in 150 μl of water. The blood spot samples taken from the malaria patients were characterized by the presence of one or more *Plasmodium* clones. Each clone was identified by a particular genotype defined by the alleles present at three loci, encoding GLURP (glutamate-rich protein), MSP (merozoite surface protein) 1 and MSP2, respectively.

In order to characterize the parasites in each blood spot, alleles from each locus were determined and interpreted as follows:

*GLURP*: alleles were characterized by size, differences in which were caused by varying numbers of a repeated unit.
*MSP2*: two sequence families (3D7 and FC27) were recognized at this locus, both of which varied in size because of different repeated numbers. In order to achieve the highest assay sensitivity, we used fluorochrome-labelled reverse primers that were specific either to the 3D7 or FC27 allelic family. Alleles were distinguished by their fluorescent dye (indicating the family) and by their size, which was determined using an automated sequencer.
*MSP1*: each allele was amplified using a PCR primer set specific to its sequence family (K1, MAD20 or Ro33).

If only one clone was present in a sample, then only one allele was found at each of the loci described above. If several clones were present, however, the number of alleles found at each locus could range between one and the number of clones. To determine whether it was a new infection or recrudescence that was causing clinical or parasitemic failure, we compared the genetic signatures of the samples at day 0 (D0) and day X (DX, at treatment failure). If at least one identical allele was found in these paired samples at each of the three loci, the failure was classified as recrudescent. If only new alleles were observed at one or more loci at DX, the case was classified as a new infection. When the amplification reaction for one locus proved to be negative for either one or both of the paired samples, the outcome was classed as indeterminate. Outcomes were classified as protocol violations when *P*. *falciparum* was not identified in the samples collected at D0. All analyses, gel electrophoresis and data entries were double-checked by a second technician. GeneTools software version 4.03.05.0 (Synoptics Ltd, Cambridge, England) was used to score fragments of *GLURP* and *MSP1*, and GeneMapper version 4.1 (Applied Biosystems Inc., CA, USA) was used for *MSP2*.

The alleles recovered were binned according to allele size (base pairs, bp): 50 bp for GLURP; 3 bp for MSP2; and 25 bp for MSP1. The highly divergent alleles found at the MSP2 locus were grouped into dimorphic families known as FC27 and 3D7. Likewise, the MSP1 alleles were grouped into three families: K1, Mad20 and RO33. The stepwise genotyping strategy chosen for this study led to the genotyping of a different number of samples using each marker. In order to avoid bias due to repeated counts of alleles within one patient with multiple infections, the basal level of allelic patterns and genetic diversity was initially assessed using D0 samples only.

We then calculated and compared the allelic patterns and level of genetic diversity described by each marker between the baseline (D0) and recurrent infections (DX), where Na = number of alleles, Na frequency ≥5% = alleles found with a frequency ≥5% and *He* = expected heterozygosity, i.e. a measure of the degree of polymorphism in each marker. The infections were classified as baseline samples (D0) or recurrent infections (DX) and then according to the number of *P*. *falciparum*-carrying clones: monoclonal (1 allele per locus) or polyclonal infection (≥2 alleles in at least one locus). Multiplicity of infection (MOI, minimal number of distinct haplotypes or “strains” within a sample) was estimated using the locus with the highest number of alleles as a proxy [17].

### Sample size and data analysis

The sample size was calculated on the basis of the randomized phase of the Quinact trial (reported separately). Recruitment in the first phase was to be continued until the number of failures required for the RCT phase was reached [14]. Data were handled using the DataFax system (Clinical DataFax Systems Inc., Ontario, Canada), in collaboration with the Infectious Diseases Institute, Makerere University, Uganda, where the database was located. Stata software version 12 (Stata Corp, Lakeway, College Station, Texas, USA) was used for the analyses.

Frequencies, percentages, means and medians were obtained using descriptive statistics. The association between MOI and treatment failure was determined with Kaplan Meier survival analysis and a Cox proportional hazard model adjusted for age, D0 parasite density, Hb concentration and temperature. The proportions of polyclonal infections at D0 and DX were compared using a Pearson χ^2^ test and average MOIs compared using the Mann-Whitney U test. The allelic patterns—number of alleles and private alleles (alleles unique to a single group, i.e. D0 or DX) and level of genetic diversity (*He* = expected heterozygosity, ranges from 0 to 1, from no diversity to highly diverse)—were calculated using GenAlEx v.6.5 [18,19]. The odds of having a polyclonal infection were evaluated by means of a logistic regression controlling for age, parasite density, Hb concentration and presence of fever in D0 infections. Estimates of risks were based on survival analysis approaches, in which events were defined as recrudescence and data were censored for new infections and the end of observation. The curves compared the risk of encountering PCR-corrected treatment failure when either a monoclonal (MOI = 1) or polyclonal (MOI≥2) infection was observed at D0.

The primary outcome was adequate clinical and parasitological response (ACPR) at day 42, as the passive form of follow-up used in this study phase did not allow us to establish treatment outcomes according to the standard WHO classification [20]. We considered late treatment failure to be any failure occurring from day 14 onwards, as this was the threshold for recruitment in the RCT phase. ASAQ efficacy was assessed using per-protocol analysis. Patients with indeterminate results and/or protocol violations were not taken into account when adjusting treatment efficacy to PCR.

### Ethical approval

The study protocol was approved by the ethical committees of the University of Antwerp (Reference: UA-A11-02) and the School of Public Health, University of Kinshasa (Reference: ESP/CE/012B/2012). Parents or guardians were asked to sign (or thumb-print if they were illiterate) informed consent forms. The study was externally monitored by the Amsterdam Institute for Global Health and Development in order to guarantee the quality of the data. The study team complied with good clinical and laboratory practice requirements. The protocol was registered under the references NCT01374581 and PACTR201203000351114 in ClinTrials.gov and in the Pan African Clinical Trials Registry, respectively.

## Results

### Trial profile and baseline characteristics

**Fig 1:**
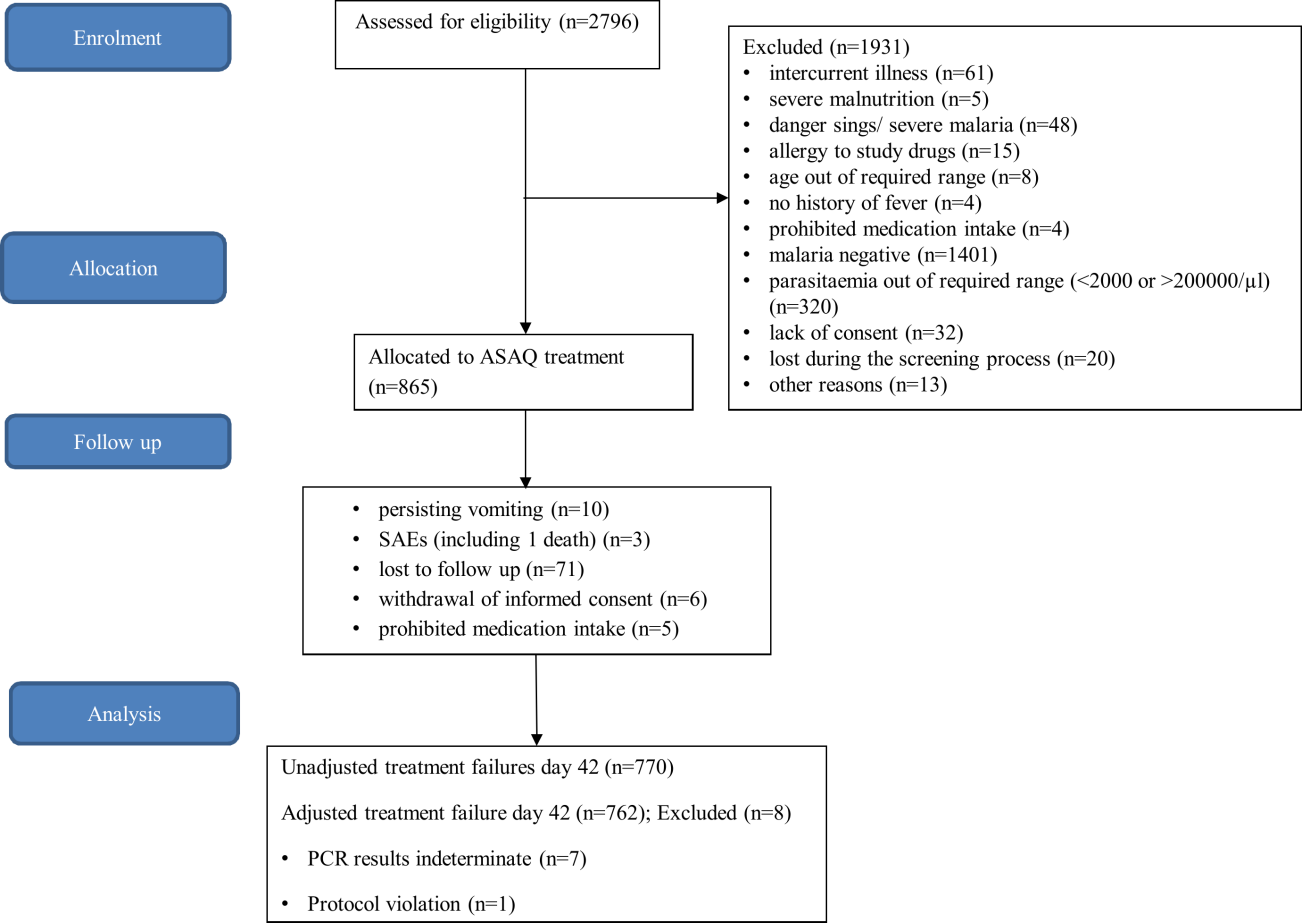
Trial profile of 42 days of passive follow-up after ASAQ treatment in Lisungi, Kinshasa, DR Congo.

A total of 2796 patients were screened for eligibility in the first phase of the Quinact trial between August 2012 and January 2014. Of these, 145 patients (5.2%) were excluded on clinical bases. Subsequently, 2651 patients (94.8%) were tested for malaria; more than half tested negative (52.8%). In total, 865 patients fulfilled the selection criteria and were treated with ASAQ. At recruitment, the mean age was 36.2 months (SD: 13.2) and mean Hb was 9.3 g/dl (SD: 1.6). Approximately one third of patients (34.8%) had slept under a mosquito net the night before enrollment (Table 1). Those who had not slept under a mosquito net were considered to be at greater risk of recurrent malaria (p<0.001). During follow-up, 95/865 patients (11.0%) were excluded from the study due to persisting vomiting (n = 10), serious adverse events (SAEs) (n = 3), loss to follow-up (n = 71), withdrawal of informed consent (n = 6) or intake of prohibited medication (n = 5) (Fig 1). SAEs consisted of hospitalizations and one case resulted in death, but none of these events were judged by the investigators to be related to ASAQ intake. Ultimately, 770 patients (89.0%) completed follow-up and were included in the analyses. Patients who did not complete follow-up had comparable baseline characteristics to the per protocol group.

**Table 1:** Demographic and baseline clinical characteristics of study participants receiving ASAQ treatment at Lisungi Health Center, Kinshasa, DR Congo.

Characteristic	ASAQ n = 865
Age (months, mean (SD))	36.2 (13.2)
Male (n (%))	442 (51.1)
Weight (kg, median (IQR))	12.5 (11.0–14.7)
Height (cm, median (IQR))	91.0 (82.9–99.5)
Slept under mosquito net the previous night (yes, n (%))	301 (34.8)
Tympanic temperature (°C, median (IQR))	37.6 (37.1–38.4)
Temperature ≥38°C (yes, n (%))	319 (37.0)
Hemoglobin concentration (g/dl, mean (SD))	9.3 (1.6)
Asexual parasites/μl (geometric mean (95%CI))	23,007 (21,047–25,149)
Gametocyte carriage (yes, n (%))	19 (2.2)
Sick in the last two months (yes, n (%))	495 (57.2)

SD, standard deviation; IQR, interquartile range; CI, confidence interval

### Medical history

The parents/guardians of 495 patients (57.2%) reported that their child had been sick in the last two months, but information about the condition was provided in only 106 (21.4%) of these cases. The most frequently reported condition was malaria, including co-morbidities (58.5%). Fever not clearly related to disease was reported by 41 (38.7%) parents/guardians. The parents/guardians of 278 patients were able to describe individual treatment courses prescribed during the month before enrollment. These had either been prescribed at health facilities or represented self-medication. The most common medication class that participants had received in the month before enrollment was antipyretics (44.2%), followed by antimalarial drugs (15.1%) and antibiotics (8.6%).

### Clinical and biological features

**Fig 2:**
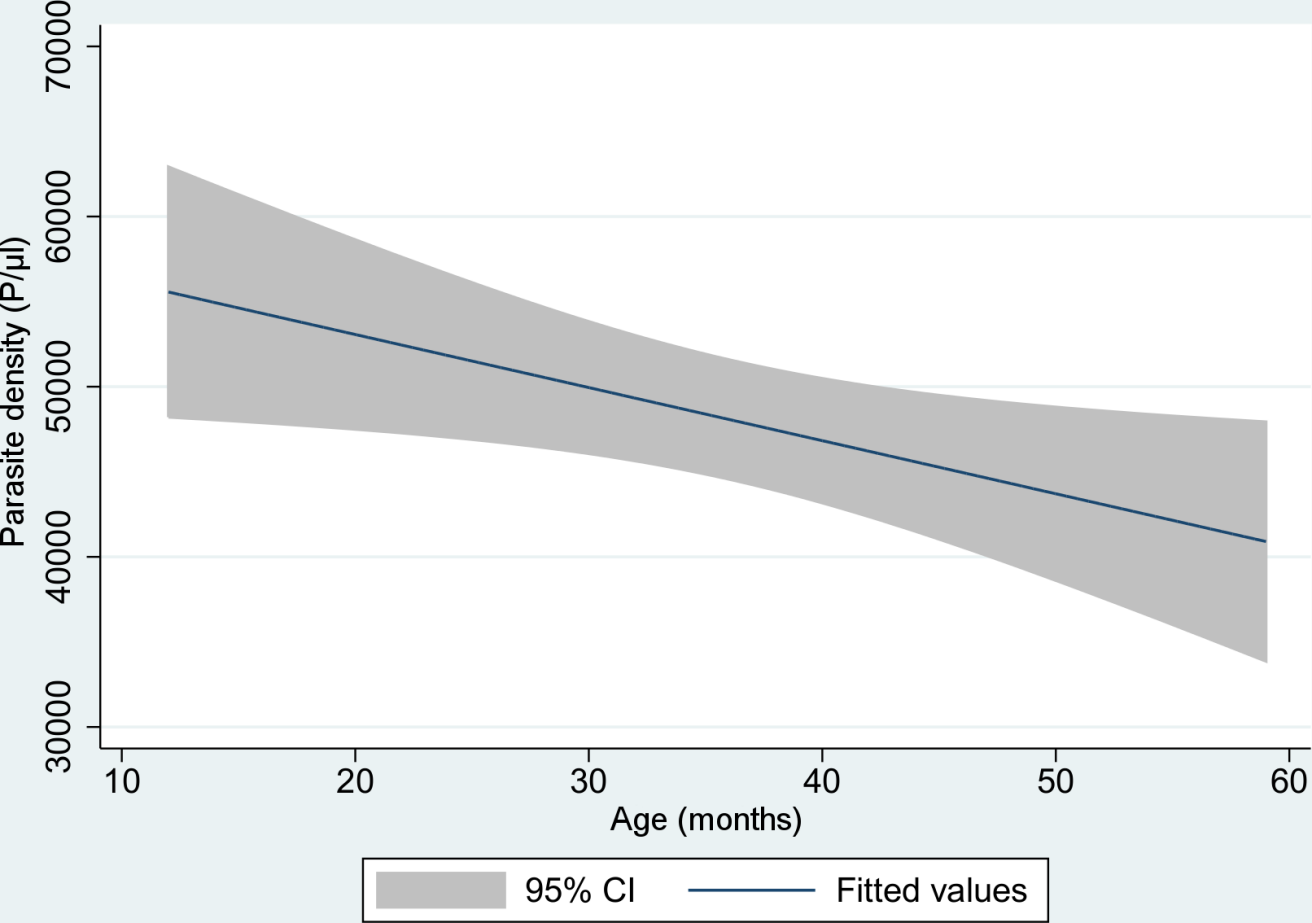
Fitted line plot of parasite density (P/ μl) according to age (months) among children under the age of five (shaded area is 95%CI).

All participants exhibited fever and/or history of fever, and this was the key symptom for screening. The most frequent symptoms were weakness, cough, behavioral changes and coryza. Jaundice and dehydration were rare, but chest examinations revealed abnormal results in 78% of patients (Table 2). Parasite density tended to diminish in older children (Fig 2).

**Table 2:** Baseline symptoms and signs before 42-day passive follow-up after ASAQ treatment in Lisungi, Kinshasa, DR Congo (865 patients).

	Absent	Mild	Moderate	Severe
Fever in the last 24 hours	-	-	865 (100)	-
Weakness	349 (40.4)	263 (30.4)	249 (28.7)	4 (0.5)
Vomiting	563 (65.1)	158 (18.3)	144 (16.6)	-
Allergic reaction	849 (98.2)	1 (0.1)	15 (1.7)	-
Diarrhea	709 (82.0)	134 (15.5)	22 (2.5)	-
Cough	399 (46.1)	275 (31.8)	157 (18.2)	34 (3.9)
Pruritus	683 (78.9)	90 (10.4)	89 (10.3)	3 (0.4)
Behavioral changes	320 (37.0)	293 (33.9)	251 (29.0)	1 (0.1)
Convulsion	861 (99.5)	-	-	4 (0.5)
Coryza	347 (40.1)	246 (28.4)	272 (31.5)	-
Muscle and joint pain*	371 (87.3)	24 (5.7)	30 (7.0)	-
Headache*	256 (60.2)	66 (15.5)	103 (24.3)	-
Nausea*	403 (94.8)	21 (4.9)	1 (0.3)	-
Dizziness*	416 (97.9)	8 (1.9)	1 (0.2)	-
Abdominal pain*	190 (44.7)	107 (25.2)	126 (29.6)	2 (0.5)
Tinnitus*	419 (98.6)	2 (0.5)	4 (0.9)	-
Jaundice	828 (95.7)	37 (4.3)	-	NA
Dehydration	848 (98.0)	14 (1.6)	3 (0.4)	NA
	**Normal**	**Abnormal**		
Chest examination	190 (22.0)	675 (78.0)	-	-
Abdominal examination**	568 (65.8)	295 (34.2)	-	-
Skin examination	668 (77.1)	197 (22.9)	-	-

NA, not applicable

*applicable only for children > 36 months old (n = 425)

**out of 863 (not performed for two patients)

### Allelic patterns and genetic diversity

The three markers showed high levels of polymorphism (*He*: 0.90–0.97). MSP2 was found to be the most polymorphic, exhibiting up to 114 different alleles (only two alleles with a frequency of above 5%), while 20 and 19 alleles (35% and 47.4% of total alleles with a frequency above 5%) were discovered for MSP1 and GLURP, respectively (Table 3). The levels of genetic diversity (*He*) of the three markers did not change among baseline samples (D0) or recurrence after treatment (DX). MSP2 revealed the largest number of private alleles in D0 and DX (26.3% and 17.5%), followed by MSP1 (15.0% and 19.0%) and GLURP (5.3% in both) (Table 3). The proportion of private alleles in the total number of alleles for each case is indicated between parentheses.

**Table 3:** Allelic patterns and genetic diversity of *P*. *falciparum* in baseline and recurrent infections.

Marker		GLURP	MSP2			MSP1			
Allelic family			3D7	FC27	overall MSP2	K1	Mad20	RO33	overall MSP1
Number of samples analyzed	D0	338	135	126	146	60	43	37	64
	DX	334	124	114	146	60	33	41	65
Allele range (bp)	overall	584–1564	168–618	91–639		140–389	126–362	199–256	
Number of alleles detected	D0	929	344	217	561	187	64	43	294
	DX	787	263	201	464	144	52	41	237
Na frequency ≥5%	D0	19 (9)	88 (1)	26 (5)	114 (2)	11 (7)	6 (5)	3 (3)	20 (7)
	DX	19 (7)	75 (0)	29 (5)	104 (3)	10 (6)	10 (6)	1 (1)	21 (7)
Number of private alleles	D0	1 (5.3%)	21	9	30 (26.3%)	1	0	2	3 (15.0%)
	DX	1 (5.3%)	8	12	20 (17.5%)	0	4	0	4 (19.0%)
*He *	D0	0.91	0.98	0.86	0.97	0.86	0.74	0.26	0.91
	DX	0.89	0.98	0.87	0.97	0.85	0.79	0.00	0.90

D0, day of recruitment; DX, day of treatment failure; bp, base pairs; Na, number of alleles; He, level of genetic diversity

Within-host parasite diversity, multiplicity of infection (MOI) and risk of recrudescence

**Fig 3:**
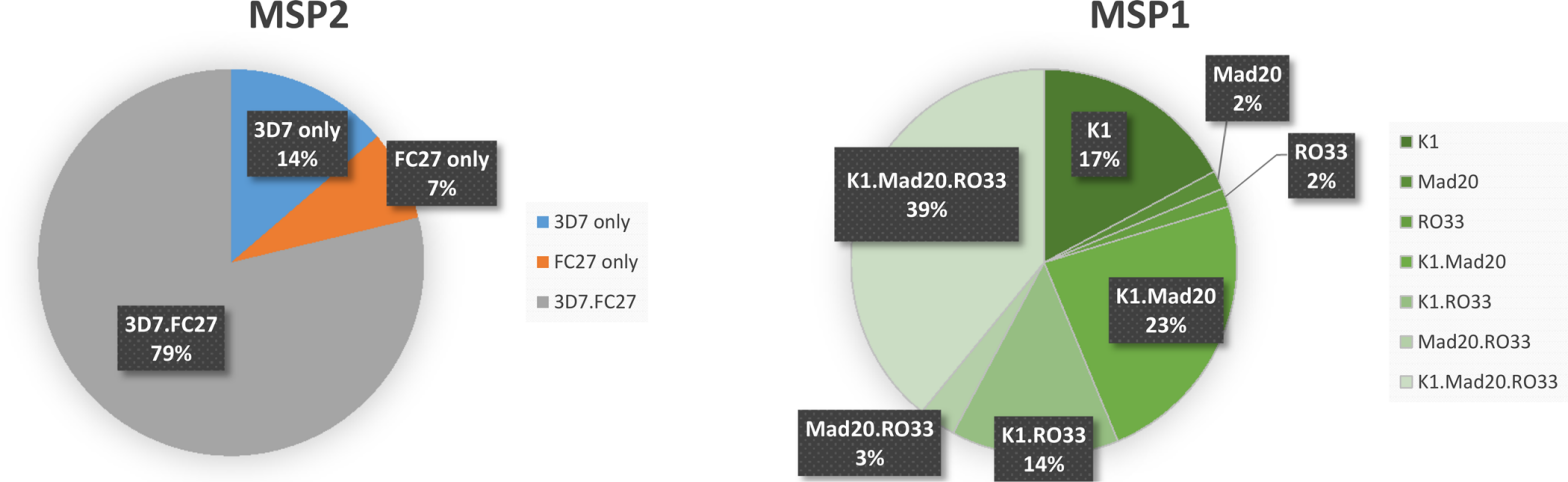
Distribution of the allelic families of MSP2 (3D7 and FC27) and MSP1 within baseline infections.

The majority of infections (78.8% of 146) contained a mixture of MSP2 family-specific alleles (3D7 and FC27), while only 13.7% and 7.5% exhibited parasites carrying only alleles 3D7 or FC27, respectively. Similarly, most infections contained a mixture of MSP1 family-specific alleles (79.7% of 64); and infections containing alleles from all three MSP1 allelic families reached 40%. Among infections containing parasites carrying only one MSP1 allelic family, K1 alleles were found to be more frequent (17.2%) than Mad20 and RO33 (1.6% in both cases) (Fig 3). Multiplicity of infection was not associated with parasite density (OR = 1.0, 95%CI: 0.99–1.00), age (OR: 0.99, 95%CI: 0.97–1.02) or Hb (OR: 1.06, 95%CI: 0.85–1.31), but did appear to be associated with fever (OR: 3.10, 95%CI: 1.83–17.52).

**Fig 4:**
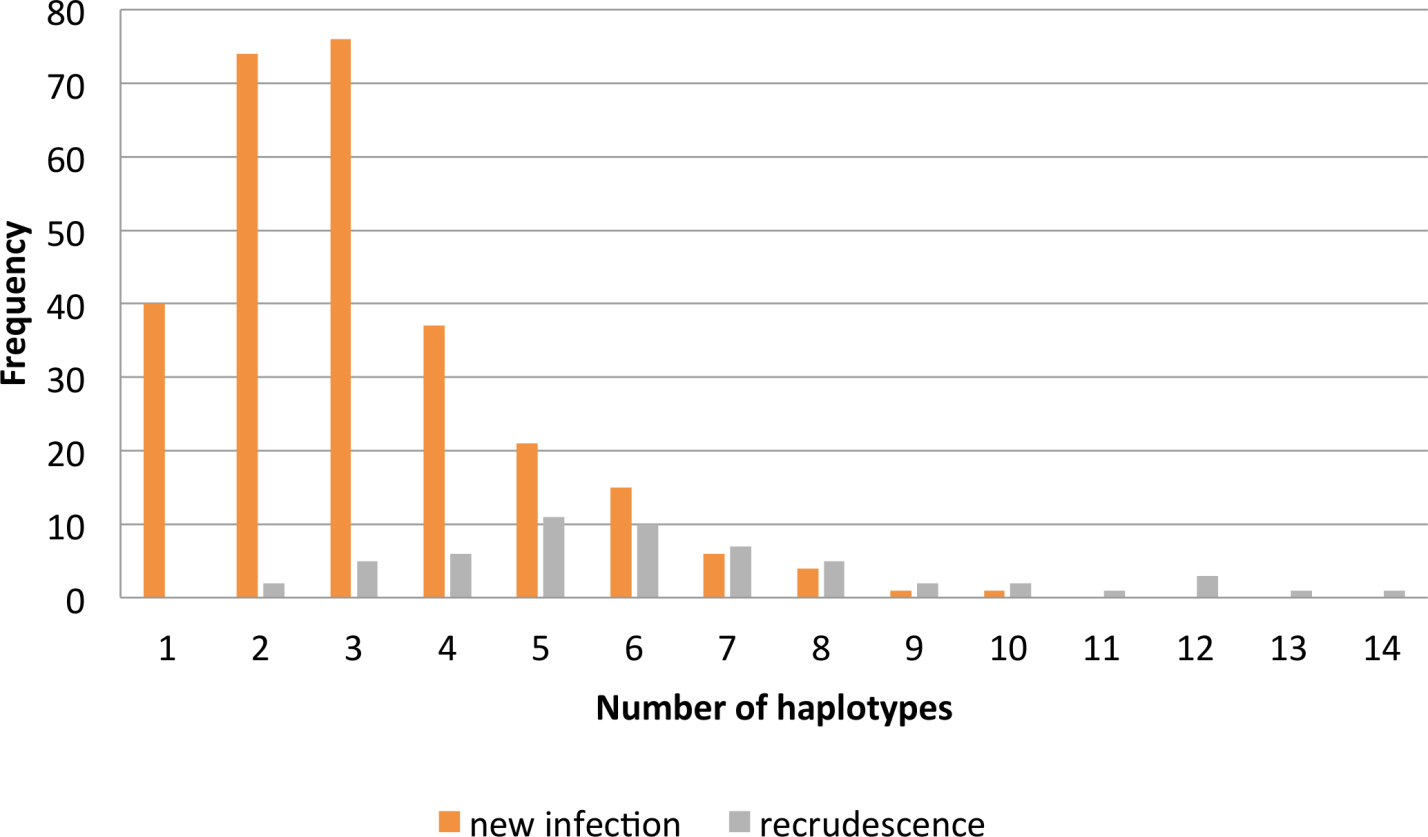
Frequency of MOI in new infections and recrudescences after ASAQ treatment failure.

In total, 64 and 146 samples (both monoclonal and polyclonal) were genotyped using MSP1 and MSP2, respectively. The infections contained alleles belonging either to a single allelic family or to two or three different families. Multiple alleles belonging to the same family were found within one infection. Polyclonal infections were more frequent at D0 and DX (88.1% and 80.1%, respectively) than were monoclonal infections (p = 0.005). The median MOI found in recurrence samples (MOI = 3; IQR: 1–5) was lower than the MOI found in D0 samples (MOI = 3.7; IQR: 0.7–6.7) (p<0.001). The odds of having a polyclonal infection were 1.8 times higher (95%CI: 1.20–2.8) for pre-treatment samples than for recurrences. Among patients with treatment failure, those with new infections were found to be carrying up to 10 haplotypes, while those with recrudescence were harboring up to 14 haplotypes (Fig 4).

**Fig 5:**
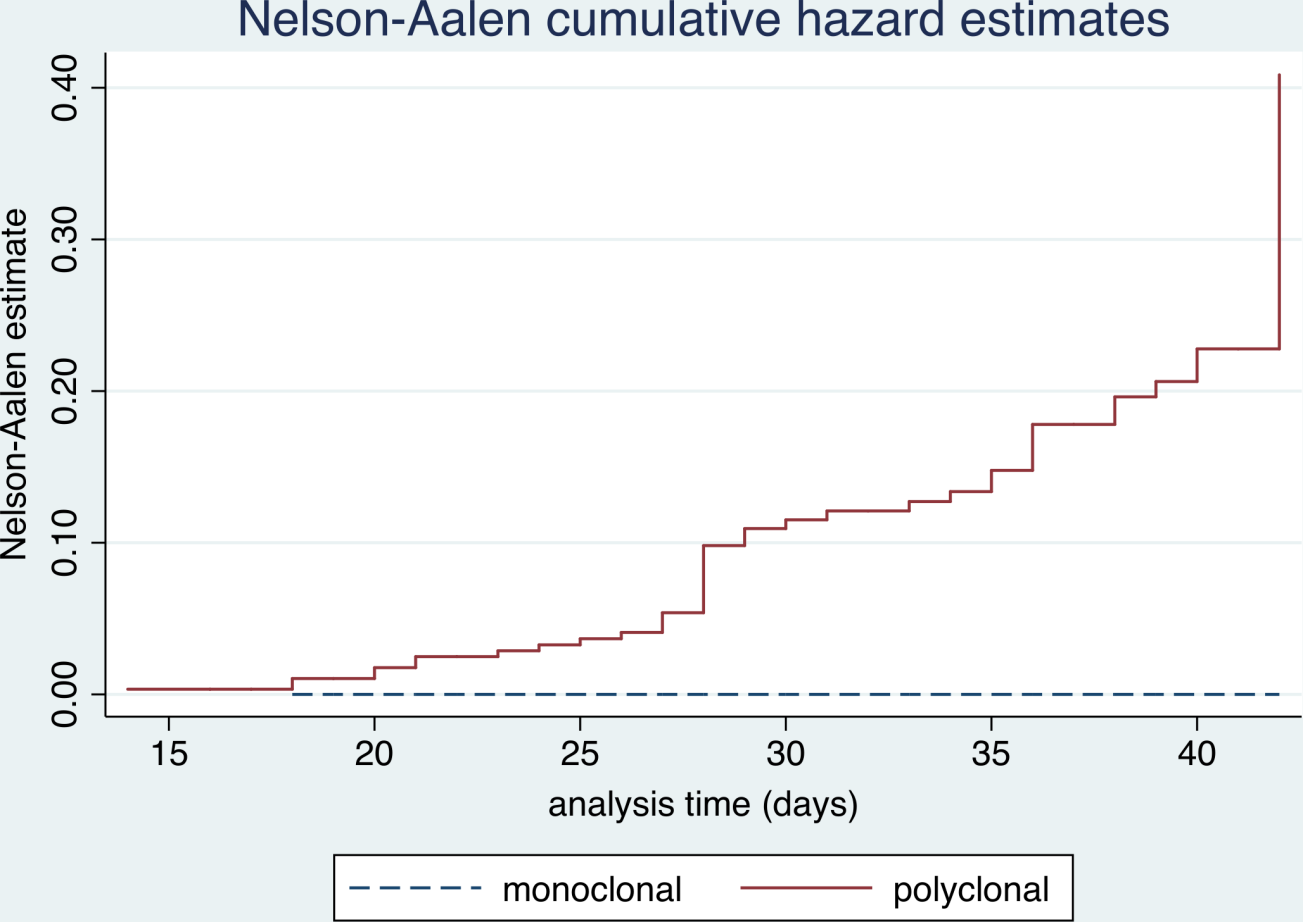
Multiplicity of infection in D0 infections and risk of recrudescence after 42 days of follow-up.

No risk of recrudescence was found when the D0 infection was monoclonal (Fig 5), while polyclonal infection was related to a recrudescence risk of 19.2% (p = 0.002). Similarly, the Cox hazard model states that the risk of treatment failure increases by 28% for each additional *P*. *falciparum* haplotype found within the D0 infection (HR: 1.3, 95%CI: 1.24–1.44, p<0.001).

### Treatment outcome

No clinical failure occurred before day 14. Forty-five percent of patients experienced treatment failure during the 42-day follow-up period (Table 4). PCR-corrected results revealed that 83.6% (95%CI: 79.1–87.2) of patients had a new infection. ASAQ efficacy after PCR adjustment was 92.8% (95%CI: 91.0–94.6). PCR confirmed that two participants had dual infections, including *P*. *ovale*, at recruitment, but neither of them experienced *P*. *ovale*-related recurrence.

**Table 4:** ASAQ treatment outcomes during passive follow-up in Lisungi, Kinshasa, DR Congo (770 patients).

	PCR uncorrected n/N (%)	PCR corrected n/N (%)
**Early clinical failure**	0	-
**Late clinical failure**	290/770 (37.7)	-
**Late parasitological failure**	54/770 (7.00)	-
**Total failure**	344/770 (44.7)	-
New infections	-	281/336 (83.6)
Recrudescence	-	55/336 (16.4)
Indeterminate	-	7
Protocol violations*	-	1
**ACPR**	426/770 (55.3)	707/762 (92.8)

ACPR, adequate clinical and parasitological response

*PCR revealed that this patient had a *P*. *malariae* and *P*. *ovale* mixed infection at recruitment and *P*. *falciparum* at failure.

## Discussion

This study highlights the clinical and molecular features of malaria in Kinshasa, DR Congo, and provides up-to-date data on ASAQ efficacy when treating uncomplicated malaria in children under five years of age. Malaria is clinically characterized by non-specific symptoms which may mimic flu-like syndromes and even lead to misdiagnosis of gastrointestinal infection [21]. Sixty percent and 54% of our study participants presented with coryza and cough, respectively, a finding which underscores the relationship between parasitemia and non-specific symptoms. Considering the prevalence of asymptomatic malaria infection—up to 30.9% around the study site [22]—it is likely that many patients attend the hospital when another cause of fever arises and that this contributes to the appearance of asymptomatic *Plasmodium* infection. In Nigeria, upper respiratory tract infections have been reported in 70% of patients testing positive for malaria in an outpatient department [23].

Furthermore, more research is needed to explore the causes of fever in the study area; a study conducted by D’Acremont *et al* in Tanzania, for example, has succeeded in revealing causes of fever beyond malaria [24]. However, malaria should be treated whenever the *Plasmodium* infection is confirmed, and co-morbidities should be managed accordingly. Our data suggest that parasite density tends to decrease as age increases (Fig 2), most likely as a result of developing immunity.

The proportion of patients with ACPR at day 42 was 92.8% after PCR adjustment. However, the proportion of recurrent malaria was high, at 44.7%. Onyamboko *et al* have already reported a similar finding in Kinshasa, namely a PCR-corrected ACPR of 93.7%, but accompanied by a much lower level of recurrent infections (27%) [6]. This discrepancy indicates that malaria features may be heterogeneous in one area, as highlighted by Mvumbi *et al* [25]. The relatively high number of recurrent malaria observed in this study could be attributed to various factors. One is the higher number of strains that were involved, which may have led to slower development of immunity in the study population, as suggested by Branch *et al* [26]. Another possible factor is the poor prophylactic effect of amodiaquine. In combination treatment for malaria, the artemisinin derivative is intended to clear the parasites quickly, as demonstrated by the lack of early treatment failures. The partner drug, which has a longer half-life, is expected to clear the remaining parasites and offer protection against new infections. We refer only to amodiaquine here, because mutations conferring resistance to artemisinin have not yet been identified in Africa [8–10]. The level of resistance to amodiaquine may be increasing, and affecting its prophylactic effect. Yet, two previous studies were unable to report concerns about amodiaquine resistance [27,28]. It is essential that resistance to amodiaquine, and other antimalarial drugs, be monitored on a regular basis. Levels of malaria transmission may also have played a role. Roughly half of our patients tested positive for malaria: a much higher proportion than the 20% reported by Muhindo *et al* [29]. The latter was an average for the city of Kinshasa as a whole, however, while almost all of the patients screened in our study were living in one particular area. Nonetheless, entomological studies are still needed to assess the level of malaria transmission. Furthermore, poor utilization of mosquito nets increases the risk of malaria recurrence. Host susceptibility could also be a factor explaining the relatively high proportion of recurrent malaria found in our study, but immunological assessment is needed.

In general, the patients screened in our study proved to be infected by a high number of *P*. *falciparum* strains. Children under five years of age carry the majority of the strains in circulation because of their vulnerability to malaria infection [15]. The results presented here are likely to reflect the reality of *P*. *falciparum* diversity in the study area and therefore represent baseline knowledge of the diversity and multiplicity of infection in Kinshasa. In addition, most malaria cases identified in this study were polyclonal infections. Some studies have demonstrated a positive association between high *P*. *falciparum* diversity and MOI, on the one hand, and high malaria transmission, on the other [30–33], though this association was not found in Papua New Guinea [34].

The odds of having a polyclonal infection were lower among recurrences than at enrollment. In order to reinforce this trend, which probably reflects the impact of effective medication; joint strategies for malaria prevention (e.g. insecticide-treated mosquito nets, intermittent preventive treatment and malaria vaccines) are needed to reduce multiple-strain infections [15]. However, the impact of these strategies on *P*. *falciparum* diversity is not entirely clear. Yet, even in areas where malaria incidence has decreased by 90%, no decrease in parasite diversity has been documented [35]. This study shows that MOI impacts the subsequent risk of recrudescence. Indeed, the risk of recrudescence actually increases along with the number of haplotypes involved in the initial episode. This finding is in line with reports from Uganda [15,36]. While theoretically possible, it is extremely unlikely that patients classified by PCR as having recrudescent infections had been re-infected by the same strains [37]. In contrast to the study by Kyabayinze *et al* [15], we found that patients presenting with fever at recruitment (tympanic temperature ≥38°C) were more likely to be infected by more than one haplotype.

The passive form of follow-up used in this study is likely to have had an impact on the estimation of the proportion of recurrent infections. If the standard procedures for antimalarial efficacy follow-up were applied in the study—with systematic malaria screening on days 1, 2, 3, 7, 14, 21, 28, 35 and 42 [20]—the study site could have been overloaded. As a result, the study team avoided active follow-up in order to maintain the quality of the data generated. Despite the limitations of the study design chosen, it did allow us to update estimates of ASAQ efficacy in Kinshasa. In addition, it provided some insight into the diversity of malaria parasites, in line with the Plasmodium Diversity Network Africa targets [38].

## Conclusions

ASAQ remains a suitable treatment for uncomplicated malaria in Kinshasa, with a PCR-corrected efficacy of 92.8% after 42 days of follow-up. The proportion of crude treatment failures was relatively high in this study, perhaps because of the poor prophylactic effect of amodiaquine, the high level of malaria transmission or the high host susceptibility to malaria among children participating in the study. Infection with a single haplotype of *P*. *falciparum* was found to predict a positive treatment outcome. Therefore, reducing the diversity of circulating *P*. *falciparum* may be key to slowing down the spread of resistant strains.

This study emphasizes the need for further research on *in vitro* amodiaquine resistance profiles and the consequences of MOI on treatment efficacy, malaria transmission and immunity development; and entomological studies for assessing malaria transmission levels. *In vivo* efficacy studies of ACTs are also of paramount importance.

## Supporting Information

S1 TREND Checklist (PDF)

S1 Protocol Quinact protocol published in Trials Journal. (PDF)

S2 Protocol Quinact protocol. (PDF)
