# Supplementary material for: Uncomplicated Clinical Malaria Features, the Efficacy of Artesunate-Amodiaquine and Their Relation with Multiplicity of Infection in the Democratic Republic of Congo
Source: PLoS One. 2016 Jun 9;11(6):e0157074. doi: 10.1371/journal.pone.0157074 (PMC4900589; doi:10.1371/journal.pone.0157074)
Supplement: S1 Protocol — (PDF) [file pone.0157074.s002.pdf]

Muhindo Mavoko et al. Trials 2013, 14:307
http://www.trialsjournal.com/content/14/1/307

STUDY PROTOCOL Open Access

Impact of retreatment with an artemisinin-based combination on malaria incidence and its potential selection of resistant strains: study protocol for a randomized controlled clinical trial

Hypolite Muhindo Mavoko[1*], Carolyn Nabasumba[2], Halidou Tinto[3], Umberto D'Alessandro[4,5], Martin Peter Grobusch[6], Pascal Lutumba[1] and Jean-Pierre Van Geertruyden[7]

Abstract

Background: Artemisinin-based combination therapy is currently recommended by the World Health Organization as first-line treatment of uncomplicated malaria. Recommendations were adapted in 2010 regarding rescue treatment in case of treatment failure. Instead of quinine monotherapy, it should be combined with an antibiotic with antimalarial properties; alternatively, another artemisinin-based combination therapy may be used. However, for informing these policy changes, no clear evidence is yet available. The need to provide the policy makers with hard data on the appropriate rescue therapy is obvious. We hypothesize that the efficacy of the same artemisinin-based combination therapy used as rescue treatment is as efficacious as quinine + clindamycin or an alternative artemisinin-based combination therapy, without the risk of selecting drug resistant strains.

Design: We embed a randomized, open label, three-arm clinical trial in a longitudinal cohort design following up children with uncomplicated malaria until they are malaria parasite free for 4 weeks. The study is conducted in both the Democratic Republic of Congo and Uganda and performed in three steps. In the first step, the pre-randomized controlled trial (RCT) phase, children aged 12 to 59 months with uncomplicated malaria are treated with the recommended first-line drug and constitute a cohort that is passively followed up for 42 days. If the patients experience an uncomplicated malaria episode between days 14 and 42 of follow-up, they are randomized either to quinine + clindamycin, or an alternative artemisinin-based combination therapy, or the same first-line artemisinin-based combination therapy to be followed up for 28 additional days. If between days 14 and 28 the patients experience a recurrent parasitemia, they are retreated with the recommended first-line regimen and actively followed up for another 28 additional days (step three; post-RCT phase). The same methodology is followed for each subsequent failure. In any case, all patients without an infection at day 28 are classified as treatment successes and reach a study endpoint. The RCT phase allows the comparison of the safety and efficacy of three rescue treatments. The prolonged follow-up of all children until they are 28 days parasite-free allows us to assess epidemiological-, host- and parasite-related predictors for repeated malaria infection.

Trial registration: NCT01374581 and PACTR201203000351114

Keywords: Malaria, Artemisinin-based combination treatment, Artesunate-amodiaquine, Artemether-lumefantrine, Quinine, Clindamycin, Randomized trial, Democratic Republic of Congo, Uganda

* Correspondence: mavoko@yahoo.com
[1]Département de Médecine Tropicale, Faculté de Médecine, Université de Kinshasa, B.P. 747 Kin XI, République Démocratique du Congo
Full list of author information is available at the end of the article

BioMed Central

© 2013 Muhindo Mavoko et al.; licensee BioMed Central Ltd. This is an Open Access article distributed under the terms of the Creative Commons Attribution License (http://creativecommons.org/licenses/by/2.0), which permits unrestricted use, distribution, and reproduction in any medium, provided the original work is properly cited.

Background

Malaria remains one of the great infectious killers in Africa. An estimated 300 to 500 million cases occur each year, causing 1.5 to 2.7 million deaths, primarily in children under the age of 5 years [1]. The reduction of malaria-associated morbidity and mortality relies largely on chemotherapy. Considering these facts, for the foreseeable future the major intervention available for the control of malaria (and a key Roll Back Malaria priority) remains the prompt treatment of symptomatic malaria with effective therapy. However, the success of this strategy has been greatly affected by the increasing resistance of malaria parasites to available drugs. This results in increased progression of disease from uncomplicated to complicated forms, and increased mortality [2]. Therefore, choices of the best treatment for uncomplicated malaria in Africa have become increasingly complex.

Following the World Health Organization (WHO) guidelines, most African countries have already opted for artemisinin-based combination therapy (ACT). Several clinical trials on artesunate-amodiaquine (ASAQ), an ACT, completed in Africa have shown an efficacy >90% [3-6]. Furthermore, after PCR analysis, over 75% of ASAQ and artemether-lumefantrine (AL) treatment failures have been classified as new infections, while recrudescences have low parasite densities [6]. ASAQ is safe and easy administered, with a good treatment adherence [3-5]. Therefore, effectiveness may be close to efficacy. ASAQ has now been developed as a fixed-dose combination and registered. The Democratic Republic of Congo (DRC) has chosen ASAQ as first-line treatment for uncomplicated malaria. Efficacy of the six-dose regimen of AL has been demonstrated in semi-immune and non-immune populations in Asia and Africa to be consistently greater than 95%, with rapid parasite and symptom clearance and significant gametocytocidal effect [7]. In Uganda, AL has been chosen as first-line treatment for uncomplicated malaria.

In DRC and Uganda, quinine is the rescue treatment for malaria. It is cheap, widely available and generally considered to be effective but is not popular due to its side effects. Quinine has a very short half-life; therefore, repeated dosing is required. In an efficacy study of quinine and artemisinin for uncomplicated malaria in Vietnam, recrudescence rates were 16% after 7 days of quinine monotherapy [8]. In studies conducted in Gabon, neighbouring DRC, *Plasmodium falciparum in vitro* sensitivity to quinine was high and had not changed over the past decade [9]. Although quinine monotherapy shows high efficacy in the setting of clinical trials, it has considerable disadvantages, mainly because of its poor tolerability and the prolonged treatment course. Poor adherence carries a high risk of treatment failure, particularly because quinine causes a syndrome of adverse effects known as cinchonism that includes primarily tinnitus, nausea, and vertigo. Other reported side effects are high tone hearing impairment, dizziness, and hypotension as well as headache and visual disturbances [10]. As a result of these side effects, some studies have reported poor compliance to treatment. A randomized trial in Thailand reported 71% adherence. Such poor adherence to the 7-day regimen is associated with a high risk of treatment failure [11], which can contribute to the development and spread of resistance [10]. Furthermore, in current practices, patients are often retreated with the recommended first-line drug. As quinine is effective against all species of malaria, including chloroquine-resistant strains of *P. falciparum*, it remains an important drug for severe malaria, although the current trend is to replace this with intravenous artesunate, which in some settings has already occurred whilst in others it is currently happening. Therefore, quinine should be protected from resistance by rational use, as its effectiveness in uncomplicated malaria is lower than ACT [12].

Potential selection of resistant strains

This study aims to assess the possible impact of using the same first-line treatment as rescue treatment. Furthermore, as part of the study protocol, mutations in the *P. falciparum* multidrug resistance (*pfmdr*)*1* will be analyzed in order to evaluate the potential risk of selecting drug-resistant strains. The longitudinal, continued follow-up of all treatment failures will additionally enable host-, environmental- and parasite-related predictors of recurrent, recrudescent or reinfections to be identified.

Rationale

Considering the facts that (i) over >75% of treatment failure to ASAQ or AL are new infections, (ii) parasite density is low in case of recrudescence occurring from day 14 onwards, and (iii) in real-life situations patients are retreated with the same first-line drug, there is a need to assess the role of the first-line treatment as rescue treatment. This efficacy will be compared to quinine + clindamycin and another ACT treatment in line with the WHO guideline [13]. We hypothesize that retreatment with the first-line ACT treatment beyond 14 days is as efficacious as any other rescue treatment, without the risk of selecting drug-resistant strains. Furthermore, a prolonged follow-up will enable the assessment of the host-related, parasite-related and environmental risk factors for repeated malaria infection. Therefore, we will collect dried samples and serum samples for analyzing (that is, immunological and molecular biological assessment).

Trial objectives

Efficacy

The primary objectives are: (1) to show that, in children aged 12 to 59 months with recurrent uncomplicated *P. falciparum* malaria within 42 days of treatment with an ACT (ASAQ in DRC or AL in Uganda), the PCR adjusted efficacy at 28 days after retreatment with the

same artemisinin-based combination therapy is at least 90%; and (2) to estimate the relative efficacy of retreatment with the same ACT compared to treatment with quinine + clindamycin and treatment with an alternative ACT (ASAQ after first-line AL treatment or AL after first-line ASAQ treatment).

Secondary objectives are: (1) to evaluate the PCR-unadjusted efficacy at 28 days of retreatment with the same ACT and to compare it to treatment with quinine + clindamycin and treatment with another ACT (ASAQ after first-line AL treatment or AL after first-line ASAQ treatment); (2) to evaluate and compare the efficacy of AL, ASAQ and quinine + clindamycin as rescue treatment for a recurrent *P. falciparum* malaria episode occurring 2 weeks after the administration of the first-line treatment, with and without PCR adjustment; (3) to evaluate and compare the 42-day clinical efficacy of AL (Uganda) and ASAQ (DRC) for the first-line treatment of uncomplicated *P. falciparum* malaria, with and without PCR adjustment; and (4) to evaluate and compare the efficacy of the different rescue treatment regimens in terms of fever clearance time, asexual parasite clearance time, gametocytemia at days 7, 14, 21 and 28, and hemoglobin changes between day 0 and days 14 and 28.

Additional objectives are: (1) to evaluate transcriptional changes and selection of *P. falciparum pfmdr1* and other alleles following therapy with quinine + clindamycin, AL and ASAQ; and (2) to assess epidemiological-, parasitological- and host-related predictors (including genetic factors) for recurrent malaria infections (adjacent studies will be developed in separate nested-study protocols).

Safety

To evaluate the safety and tolerability of AL, ASAQ and quinine + clindamycin when used as rescue treatments.

Drugs to be tested

Quinine + clindamycin

Quinine is an alkaloid from the bark of the cinchona tree that still constitutes one of the major components of the antimalarial pharmacopeia, as it has for over three centuries. Quinine is still widely used for the treatment of severe malaria and serves in oral formulation as a reserve drug for uncomplicated malaria, mostly in combination with a tetracycline or doxycycline or clindamycin. It is effective against all species of malaria including chloroquine-resistant strains of *P. falciparum*. Quinine belongs to the aryl amino alcohol group of drugs. It is a cinchona alkaloid that has a rapid schizonticidal action on the intra-erythrocytic parasites and is also gametocytocidal for *P. vivax* and *P. malariae* but not for *P. falciparum*.

In DRC, quinine + clindamycin is the second-line treatment for malaria. In Uganda, quinine is the rescue treatment, but a combination with clindamycin might be expected in the near future. Quinine is cheap, widely available and generally considered to be effective though it is not popular due to the unwanted side effects. Quinamax® is a formulation developed by Sanofi-Aventis (Paris, France) which comprises four alkoloids: quinine, quinidine, cinchonin and cinchonidine. In this study, we use dry tablets (125 mg) with no dosage adaptation for children below 9 kg (which explains why children below 12 months are excluded from our study).

Clindamycin is a lincosamide antibiotic derivative of lincomycin. It is very soluble in water. It inhibits the early stages of protein synthesis by a mechanism similar to that of the macrolides. It may be administered by mouth as capsules containing the hydrochloride or as oral liquid preparations containing the palmitate hydrochloride. Clindamycin is used twice daily for at least 5 days at 10 mg/kg for children aged 11 years and under. Clindamycin used in combination with quinine is safe but limited data have so far been gathered [14]. In this study, we use clindamycin from Pfizer (New York City, the United States of America) marketed under the brand name of Dalacin®.

Artemether-lumefantrine

This is a fixed-dose combination of artemether (a semi-synthetic artemisinin derivative) and lumefantrine (a slowly eliminated drug also referred to as benflumetol). The registered indications and branding for AL cover treatment of uncomplicated malaria caused by mono or mixed *Plasmodium* infections. The combination is expected to confer mutual protection against resistance and prevent recrudescence after artemether therapy. The components of this combination were originally studied and developed in China by the Academy of Military Medical Sciences (Beijing and Kunming Pharmaceutical Factory, Kunming). The fixed combination has been registered in China since 1992 and has undergone further development when Novartis (Basel, Switzerland) signed a collaborative agreement in 1994 with the Academy of Military Medical Sciences and CITITEC, the technology arm of the China International Trust and Investment Corporation (Beijing, China). Studies for the international registration started in 1995. AL from Novartis, marketed under the trademarks Riamet® and Coartem®, was registered in Switzerland in 1999. It is prequalified by WHO and has since received marketing authorization in several endemic and non-endemic countries. A recent review showed that the drug combination is highly efficacious against sensitive and multidrug resistant *falciparum* malaria as it offers the advantage of rapid clearance of parasites by artemether and the slower elimination of residual parasites by lumefantrine [7].

Artesunate-amodiaquine

ASAQ (co-formulated as Co-arsucam® or ASAQ Winthrop®; Sanofi-Aventis (Paris, France) is safe, easy to use and

efficacious, and the second most used ACT worldwide [5,6]. DRC, through the National Malaria Control Program, has complied with the WHO recommendation by recommending ASAQ as first-line treatment since 2005 for uncomplicated malaria. In a study conducted in 2004 in the eastern part of the country, the efficacy of ASAQ was estimated at 93% after PCR adjustment [4]. Twenty five trials (11,700 patients) carried out in Sub-Saharan Africa show a PCR-adjusted efficacy at day 28 of 94% [6]. A study has been conducted in Burkina Faso in children under 5 years of age and has shown that co-formulated ASAQ is well tolerated and its efficacy was 93% after PCR correction [5]. We use co-formulated ASAQ Winthrop® (Sanofi-Aventis), dosed by age, and put on the market in March 2007. This product has been prequalified by WHO.

Study design

This is a bi-centre, phase IIIb, randomized, open label, three-arm trial. It is performed in three steps (Figure 1) and there is informed consent for the first two steps.

Study phases

Pre-randomized controlled trial phase

All patients are treated with the first-line treatment - ASAQ for DRC and AL for Uganda. Before treatment, a blood sample is collected on filter paper (Whatman 3MM, Maidstone England) for subsequent parasite genotyping. A serum sample is also collected and frozen for further immunological assessment (adjacent study). Patients are passively followed up for the next 42 days. During this period, no systematic screening for malaria infection is done. Instead, blood samples are collected only for patients attending the health facilities with suspected clinical malaria. In case of confirmed failure before day 14, patients are treated with quinine + clindamycin and excluded from the follow-up as they have reached one of the endpoints. Patients experiencing a clinical failure (fever and parasitemia with any parasite density) between days 14 and 42 are eligible for the second phase. The day of failure corresponds to day 0 of the following phase. Patients are assessed as summarized in Additional file 1.

Randomized controlled trial phase

This step constitutes the core of the study. After the second informed consent (that covers also the post-RCT phase), patients are randomly assigned to ASAQ, AL or quinine + clindamycin. Rescue treatment allocation is concealed until the recruitment of the patient in the RCT phase. The randomization list was generated prior to the beginning of the study by the study statistician. At the study site, treatment allocation and administration of medications are performed by the study physician or nurse. Patients are assessed as summarized in Additional file 2.

Parents/guardians are encouraged to return to the clinic for follow-up assessments on days 3, 7, 14, 21 and 28, and on any unscheduled day if the child is not well.

Post- randomized controlled trial phase

All patients included in the RCT phase and presenting a clinical or parasitological failure from day 14 onwards are retreated with the country's first-line treatment (AL or ASAQ). We expect about 50 patients per site to participate in this phase. All patients are followed up exactly as during the RCT and study procedures are also identical. In case of confirmed failure before day 14, patients are treated with the first-line ACT and excluded from further follow-up. The same procedures are followed for each repetitive treatment failure occurring between 14 and 28 days after each treatment course. The liver function and hematologic parameters are monitored in participants exposed to three ASAQ courses or more.

Patient selection

Inclusion criteria for the pre-randomized controlled trial phase

In order to be eligible, patients should satisfy the following inclusion criteria:

1. Males and females aged between 12 months and 59 months inclusively. This criterion applies only for the recruitment in the first follow-up. For the subsequent follow-up, children included in the first follow-up are eligible, regardless of their age.
2. Body weight of 9 kg and above.
3. Microscopically confirmed, mono-infection of *P. falciparum* or mixed infection containing *P. falciparum* (parasitaemia ≥ 2,000/μL to 200,000/μL).
4. Fever (tympanic temperature ≥ 38.0°C) or history of fever in the previous 24 hours.
5. Hemoglobin value ≥ 6.0 g/dl.
6. Signed (or thumb-printed and witnessed by an impartial witness whenever parents/guardians are illiterate) informed consent by the parents or guardians. Note the first informed consent will be asked at recruitment in the pre-RCT phase and it will cover the first 42 days follow-up. The second informed consent will be asked for at enrolment for the randomized trial and will cover the remaining period of the study.
7. Parents' or guardians' willingness and ability to comply with the study protocol for the duration of the study.

Exclusion criteria for the pre-randomized controlled trial phase

Patients with at least one of the following criteria will be excluded:

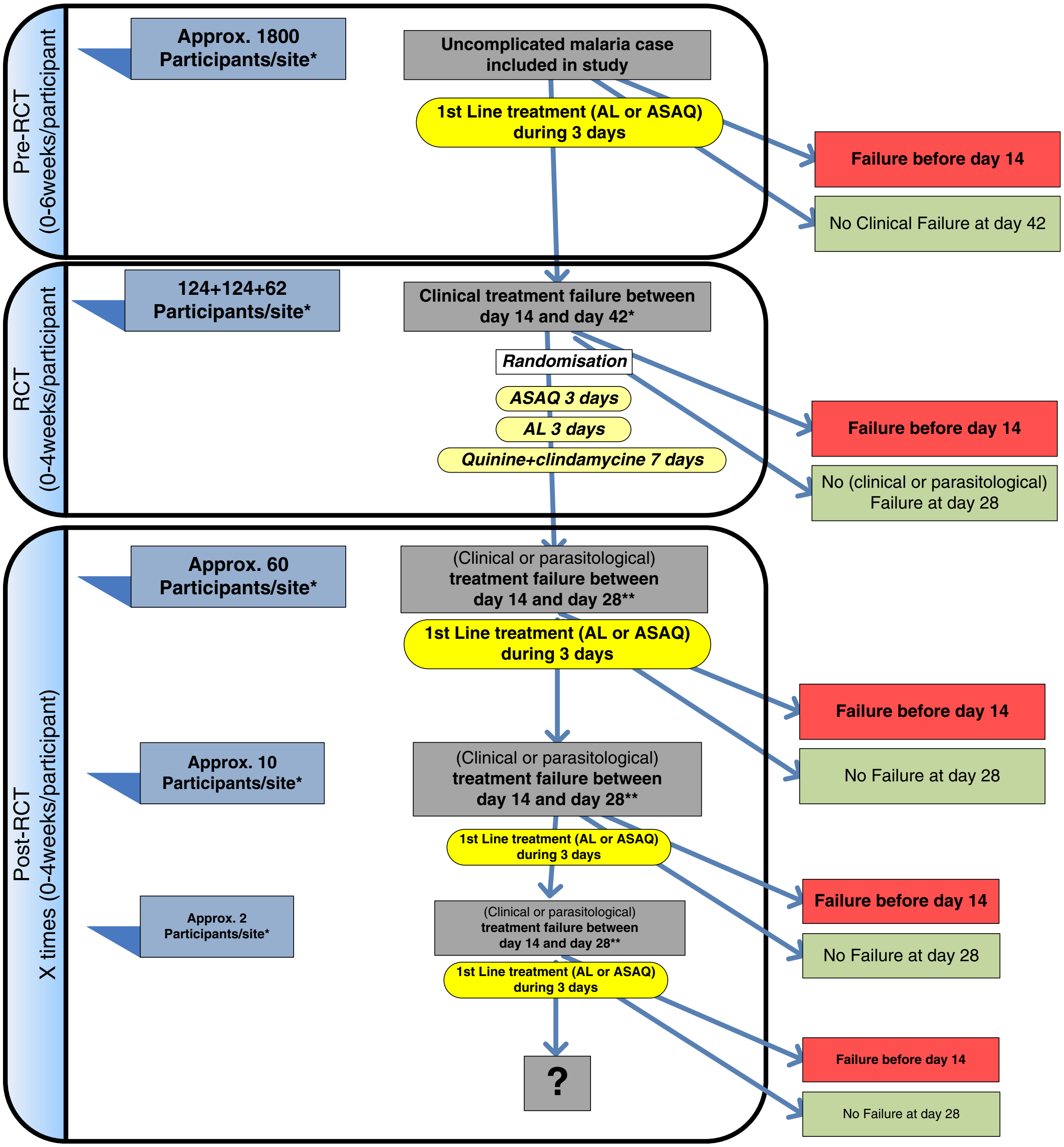

Figure 1 Study design flow chart. ACT, artemisinin-based combination therapy; AL, artemether-lumefantrine; ASAQ, artesunate-amodiaquine; RCT, randomized controlled trial.

1. Participation in any other investigational drug study (antimalarial or others) during the previous 30 days.
2. Known hypersensitivity and previous serious adverse events (SAEs) related to the study drugs.
3. Severe malaria [15] or danger signs: not able to drink or breast-feed, vomiting (more than twice in 24 hours), recent history of convulsions (more than once in 24 hours), unconscious state, unable to sit or stand.
4. Presence of intercurrent illness or any condition (cardiac, renal, hematologic, hepatic diseases) which would place the subject at undue risk or interfere with the results of the study, including known glucose-6-phosphate dehydrogenase deficiency.

5. Patients who are taking drug which may prolong the QT interval on the electrocardiogram (imidazole and triazole, antifungal agent).
6. Severe malnutrition (defined as weight for height <70% of the median National Center for Health Statistics/WHO Toxicity Grading Scale for Determining the Severity of Adverse Events).
7. Ongoing prophylaxis with drugs having antimalarial activity such as cotrimoxazole for the prevention of *Pneumocistis carinii* pneumonia in children born to HIV+ women.

Inclusion criteria for the randomized controlled trial phase

1. Have been enrolled in the first phase
2. Recurrent *P. falciparum* infection with clinical symptoms (this could be either mono or mixed infections).
3. Parents' or guardians' willingness and ability to comply with the study protocol for the duration of the study.
4. Signed (or thumb-printed whenever parents/guardians are illiterate) (second) informed consent by the parents or guardians. Note, the informed consent will cover the whole period of the study, including additional active follow-ups.

Exclusion criteria for the randomized controlled trial phase

Patients with any of the following criteria will not be admitted to the study:

1. Known hypersensitivity or serious drug-related adverse event (AE) to the study drugs.
2. Severe malaria.
3. Danger signs: not able to drink or breast-feed, vomiting (more than twice in 24 hours), recent history of convulsions (more than once in 24 hours), unconscious state, unable to sit or stand.
4. Treatment failure within 14 days in the first study phase.
5. Body weight below 9 kg.

Endpoints

Primary endpoint

The primary endpoints are: (1) PCR-adjusted efficacy at 28 days: the proportion of children with PCR adequate clinical and parasitological response (ACPR) at day 28; and (2) all early failures before day 7 plus the recurrent parasitemias detected later and classified by genotyping as recrudescence. The treatment failure is defined according to the WHO criteria [16] as the sum of early treatment failures (ETFs) and late treatment failures (LTFs) (Table 1).

The ACPR is defined as absence of parasitemia at the end of the follow-up period (day 28), irrespective of tympanic temperature without previously meeting any of the criteria of ETF or LTF. In the PCR-adjusted analyses, patients with late asexual parasite reappearance (with or without fever) will be considered ACPR if the PCR analysis shows a new infection rather than a recrudescence.

Secondary efficacy endpoints

1. PCR-unadjusted efficacy at 28 days - the proportion of children without (PCR not adjusted) treatment failure; all treatment failures detected during the active follow-up, regardless of genotyping.
2. Clinical efficacy at 42 days - all clinical treatment failures detected during the 42 days follow-up for the first-line treatment, with and without PCR

Table 1 Definition of early and late treatment failures

Early treatment failure (one of the following)	Late treatment failure
(i) Development of danger signs or severe malaria on day 0, day 1, day 2 or day 3, in the presence of parasitemia	Late treatment failure is divided into late clinical and late parasitological failure
(ii) Parasite density on day 2 > day 0 count, irrespective of tympanic temperature	Late clinical failure:
(iii) Presence of parasitemia on day 3 with fever (tympanic temperature ≥ 38.0°C)	(i) Development of danger signs or severe malaria after day 3 in the presence of parasitemia
(iv) Parasitemia on day 3 ≥ 25% of count on day 0	(ii) Presence of parasitemia and fever on any day from day 4 to day 28, without having previously met the criteria of early treatment failure
	Late parasitological failure:
	Parasitemia after day 3 in the absence of fever (tympanic temperature <38.0°C) and without having previously met the criteria of early treatment failure or late clinical failure

The treatment failure is defined according to the World Health Organization criteria [16] as the sum of early and late treatment failures.

adjustment. As no active monitoring of parasitemia after day 3 is planned, this includes ETF and late clinical failure (LCF) following WHO criteria.

3. Fever clearance time - defined as the time (in days) from the time of randomization to the first two consecutive measurements on 2 different days of tympanic temperature below 38.0°C.
4. Asexual parasite clearance time - defined as the time (in days) from time of randomization to 2 consecutive negative blood slides (collected at different days). The time to the event will be taken as the time to the first negative slide.
5. Gametocytemia (prevalence and density) at days 7, 14, 21 and 28 after treatment.
6. Hemoglobin changes between day 0 and days 14 and 28.

Tertiary efficacy endpoints

Tertiary efficacy endpoints will be defined in the substudy protocols.

Safety endpoints

Subjects will be monitored throughout the study for possible development of AEs. All AEs will be recorded on the specific form in the case report form (CRF). Vital signs and hematology will be monitored and changes in relevant laboratory parameters will be assessed.

PCR analysis

PCR plays a key role as it determines if recrudescence will be eliminated after retreatment with the first-line ACT (Figure 2). Blood samples collected on filter paper for PCR genotyping will be analyzed at the Institute of Tropical Medicine, Antwerp, Belgium. Samples will be collected according to the study-specific standard operating procedures.

Genotyping

Genotyping of the recurrent infections will be done by characterizing *msp1*, *msp2* and *glurp* genes in the *P. falciparum* genome. PCR amplification of DNA from a single parasite clone results in a single amplification product. For the three genes, each PCR amplification product of a different size is considered to originate from a different clone of *P. falciparum* and reflects a different genotype. For the samples collected from the same patient at day 0 and on the day of recurrent parasitemia, the length polymorphism of *msp1*, *msp2* and *glurp* will be determined (that is the number of bands in each PCR reaction and their respective size). Results will be interpreted as follows: recrudescence (for each marker (*msp1*, *msp2* and *glurp*), at least one identical length polymorphism is found in the sample collected at day 0 and on the day of recurrent parasitemia); new infection (for at least one marker, length polymorphism is different between the sample collected at day 0 and that on the day of recurrent parasitemia); indeterminate (samples

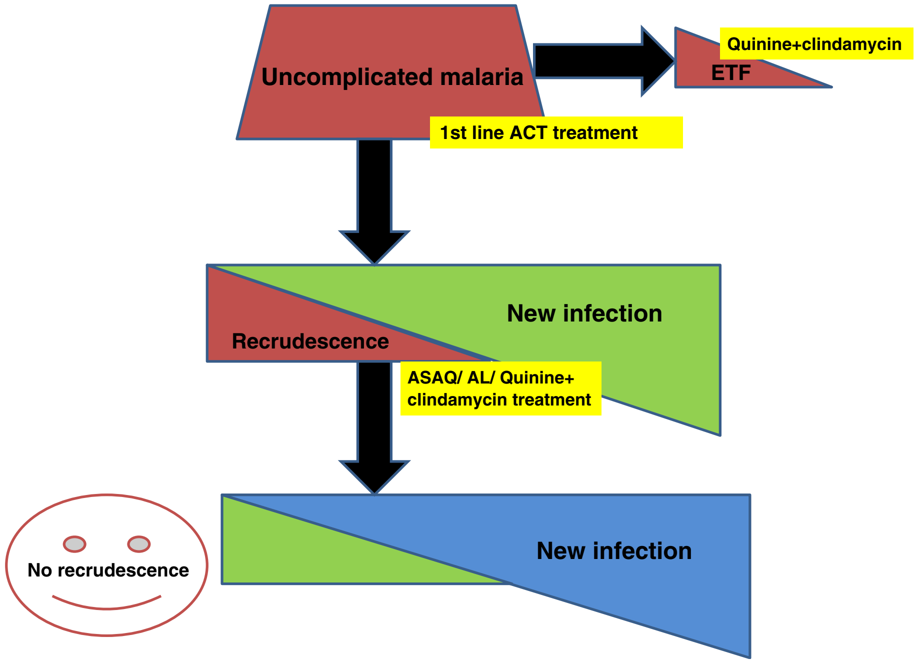

Figure 2 Role of molecular biology. ACT, artemisinin-based combination therapy; AL, artemether-lumefantrine; ASAQ, artesunate-amodiaquine; ETF, early treatment failure.

that failed to produce a result due to an inability to amplify DNA at day 0 and/or on the day of recurrent parasitemia).

Frozen serum samples
Frozen serum samples are stored on site at -70°C or at the sponsors premises until transferred for analysis if a research protocol adjacent to this study has been accepted by the relevant Ethics Committee.

Sample size
Assuming the true PCR-corrected efficacy at 28 days of retreatment with the same ACT is 95%, a sample size of 248 patients (that is, 124 per site) is needed to show with 80% power that the lower 95% confidence interval of the efficacy of retreatment with the same ACT should be 90%.

Based on the non-inferiority design, recruiting the same number of patients on the alternative ACT (that is, AL in DRC and ASAQ in Uganda) and half on quinine + clindamycin will allow the estimation of the relative risk in efficacy between the treatment groups to be within 5% assuming a similar efficacy of 95% for all three treatment groups (with the lower 95% confidence interval of the efficacy being 90%).

To allow for up to 15% drop-outs or non-evaluable patients, we will recruit 357 patients in each site. The 2:2:1 allocation ratio is chosen to reduce the number of patients randomized to the more demanding quinine + clindamycin treatment regimen. In addition, this allows greater precision for the documentation of the efficacy and safety of ACT regimens as a second line, rather than the standard rescue treatment quinine + clindamycin. However, the inclusion of the quinine + clindamycin arm is necessary as a benchmark for assessing the efficacy of the ACT as a second line and an internal consistency check of the study.

In DRC, the most recent study results [17] on the clinical efficacy of ASAQ in the Equator Province in 2004 show a PCR-uncorrected clinical failure rate of 41% at day 28. However, in Kinshasa, due to a lower reinfection incidence, the PCR-uncorrected clinical failure rate can be expected to be lower at approximately 25%. Assuming a clinical failure rate of 25% and assuming that 80% of failing patients would be eligible for the RCT and their guardians provide informed consent, we will need 1,800 children to be enrolled for the initial phase of the study in DRC to obtain 310 patients recruited in the clinical trial.

In Uganda, most recent study results on the clinical efficacy of AL in Mbarara in 2005 show a PCR-unadjusted clinical failure rate of 23% after 28 days of follow-up [18]. Empirically, expecting a clinical treatment failure rate of 25% (assuming a loss to follow-up of 10% and assuming that 80% of the failing patients would be eligible for the RCT after their guardians have provided informed consent) we will need 1,800 children to be enrolled for the initial study phase in Uganda to obtain 310 patients recruited in the clinical trial.

The recruitment will continue in each country until the required sample size in the RCT phase is reached. If recruitment rates in the RCT are lower than expected, the study may be extended to additional research sites.

Treatment administration
The study treatment is administered under the direct supervision of a study nurse. If a patient fails to return to the clinic in a timely manner for their daily dose of study drug, they are visited at home and brought to the clinic the same day. If a patient misses any dose of study drugs, they are not excluded from the study. The study nurse records the date and time that study drugs are administered. Study drugs are given to young children as specified by the manufacturer of each product. After drug administration, patients are kept for 60 minutes in the clinic. A dose is repeated in full if vomiting occurs within 30 minutes of administration, and in half if vomiting occurs between 30 and 60 minutes. If vomiting persists beyond two additional doses, the patient is withdrawn from the study and treatment changed.

Concomitant therapies
Disallowed concomitant drug therapies during the active follow-ups
Any antimalarial or antibiotic with antimalarial activity (for example, erythromycin or other macrolides, cotrimoxazole or other sulfonamides, and so forth) are disallowed. A list of drugs having antiplasmodium activity was provided to the study clinicians.

Allowed concomitant drug therapies
During the trial, patients can be prescribed drugs such as paracetamol and antibiotics with no known antimalarial activity (penicillins, cephalosporins). The dose, route, time and duration of any concomitant medical treatment is recorded in the CRF.

Special conditions
Parents or guardians are discouraged from obtaining drugs from any other source such as private pharmacies, markets or clinics. Parents/guardians are encouraged to bring their children to the study clinic if they are unwell or if they are worried about their health.

Rescue treatments
All patients who develop severe malaria during follow-up are treated following National Guidelines. Patients randomized to ACT and experiencing treatment failure

before 14 days are treated with quinine 10 mg/kg orally three times a day in combination with clindamycin 10 mg/kg twice daily for at least 5 days. Patients who fail with quinine + clindamycin therapy are treated with the first-line therapy (ASAQ or AL).

Patient withdrawal criteria
Patients will be excluded from further assessment if there is withdrawal of informed consent. SAEs related to the study drug are also a reason for withdrawal from the study. Intake of disallowed drugs leads to withdrawal of the patient from active follow-up. Participants also may be withdrawn if the study sponsor, government or regulatory authorities, or site Ethics Committee terminate the study prior to its planned end date. Every reasonable effort will be made to complete a final evaluation of participants who terminate from the study prior to the planned termination time period and study staff will record the reason(s) for all withdrawals from the study in participants' study records. At the time of withdrawal, all study procedures outlined for the end of study visit are completed. The primary reason for a patient's withdrawal from the study is to be recorded in the source documents.

Protocol violations
A protocol violation occurs when an event happens that does not allow for accurate interpretation of response to treatment. Protocol violations will be defined in the statistical analysis plan.

Safety variables
Safety and tolerability of the treatments will be evaluated by recording AEs and grading laboratory, and vital sign evaluations.

Adverse events
At each visit, the investigator will ascertain the occurrence of any AE since the last visit. Any event must be recorded on the CRF.

Definition of an adverse event
An AE is any untoward medical occurrence in a patient or clinical investigation subject administered a pharmaceutical product and which does not necessarily have a causal relationship with this treatment. An AE can therefore be any unfavorable and unintended sign (that could include a clinically significant abnormal laboratory finding), symptom or disease temporally associated with the use of a medicinal product, whether or not considered related to the medicinal product.

Examples of an AE include:

1. Significant or unexpected worsening or exacerbation of the condition under study.
2. Exacerbation of a chronic or intermittent pre-existing condition including either an increase in frequency and/or intensity of the condition.
3. New conditions detected or diagnosed after investigational product administration even though it may have been present prior to the start of the study.
4. Signs, symptoms, or the clinical sequelae of a suspected interaction.
5. Signs, symptoms, or the clinical sequelae of a suspected overdose of either investigational product or a concurrent medication (overdose as such should not be reported as an AE/SAE).
6. Significant failure of expected pharmacological or biological action.

Examples of an AE do not include:

1. Situations where an untoward medical occurrence did not occur (social and/or convenience admission to a hospital).
2. Anticipated day-to-day fluctuations of pre-existing chronic disease(s) or condition(s) present or detected at the start of the study that do not worsen.

Severity, relationship of event to study drug, and outcome
The severity of a clinical AE is to be scored according to the following scale:

1. Mild: awareness of sign or symptom, but easily tolerated
2. Moderate: discomfort enough to cause interference with usual activity
3. Severe: incapacitating with inability to work or perform usual activity
4. Life-threatening: patient at risk of death at the time of the event

Assessment of causality
The investigator is obliged to assess the relationship between the investigational product and the occurrence of each AE/SAE. The investigator will use clinical judgment to determine the relationship. Alternative causes, such as natural history of the underlying diseases, concomitant therapy, other risk factors, and the temporal relationship of the event to the investigational product will be considered and investigated. The investigator will also consult the drug information and the Data Safety and Monitoring Board (DSMB) as needed in the determination of their assessment.

There may be situations when an SAE has occurred and the investigator has minimal information to include in the initial report to the Trial Management Group (TMG). However, it is very important that the investigator always

make an assessment of causality for every event prior to transmission of the SAE report to the TMG. The investigator may change their opinion of causality in light of follow-up information, amending the SAE CRF accordingly.

The relationship of an AE to study drug is to be assessed according to the following definitions:

1. Definitely unrelated: should be reserved for those events which occur prior to test drug administration (for example, washout or single-blind placebo) or for those events which cannot be even remotely related to study participation (for example, injury caused by a third party).
2. Unlikely: there is no reasonable temporal association between the study drug and the suspected event and the event could have been produced by the subject's clinical state or other modes of therapy administered to the subject.
3. Possible: the suspected AE may or may not follow a reasonable temporal sequence from study drug administration but seems to be the type of reaction that cannot be dismissed as unlikely. The event could have been produced or mimicked by the subject's clinical state or by other modes of therapy concomitantly administered to the subject.
4. Probable: the suspected AE follows a reasonable temporal sequence from study drug administration, abates upon discontinuation of the drug, and cannot be reasonably explained by the known characteristics of the subject's clinical state.
5. Definitely related: should be reserved for those events which have no uncertainty in their relationship to test drug administration. This means that a rechallenge was positive.

The outcome of each AE must be assessed according to the following classification:

1. Completely recovered: the patient has fully recovered with no observable residual effects.
2. Not yet completely recovered: improvement in the patient's condition has occurred, but the patient still has some residual effects.
3. Deterioration: the patient's overall condition has worsened.
4. Permanent damage: the AE has resulted in a permanent impairment.
5. Death: the patient died due to the AE.
6. Ongoing: the AE has not resolved and remains the same as at onset.
7. Unknown: the outcome of the AE is not known because the patient did not return for follow-up (lost to follow-up).

Definition of a serious adverse event

An SAE or reaction is any untoward medical occurrence that at any dose fulfils at least one of the following criteria:

- Results in death.
- Is life-threatening. (Note that the term 'life-threatening' in the definition of 'serious' refers to an event in which the subject was at risk of death at the time of the event. It does not refer to an event, which hypothetically might have caused death, if it were more severe.)
- Requires hospitalization (other than for drug administration) or prolongation of existing hospitalization. (Note that, in general, hospitalization signifies that the subject has been detained (usually involving at least an overnight stay) at the hospital or emergency ward for observation and/or treatment that would not have been appropriate in the physician's office or outpatient setting. Complications that occur during hospitalization are AEs. If a complication prolongs hospitalization or fulfills any other serious criteria, the event is an SAE. When in doubt as to whether 'hospitalization' occurred or was necessary, the event should be considered an SAE. Hospitalization for elective treatment of a pre-existing condition that did not worsen from baseline is not considered an AE, nor hospitalization for non-medical reasons (for example, the patient stays at the hospital overnight because they live too far and/or there is no transport).)
- Results in persistent or significant disability/incapacity. (Note that the term disability means a substantial disruption of a person's ability to conduct normal life functions. This definition is not intended to include experiences of relatively minor medical significance such as uncomplicated headache, nausea, vomiting, diarrhea, influenza, and accidental trauma (for example, sprained ankle) which may interfere or prevent everyday life functions but do not constitute a substantial disruption.)

Medical or scientific judgment should be exercised in deciding whether reporting is appropriate in other situations, such as important medical events that may not be immediately life-threatening or result in death or hospitalization but may jeopardize the subject or may require medical or surgical intervention to prevent one of the other outcomes listed in the above definition. These should also be considered serious. Examples of such events are invasive or malignant cancers, intensive treatment in an emergency room or at home for allergic bronchospasm, blood dyscrasias or convulsions that do

not result in hospitalization, or development of drug dependency or drug abuse.

All SAEs, whether or not deemed drug-related or expected, must be reported immediately or within 24 hours (one working day), using the Serious Adverse Event Notification Form, by telefax to +32-2652875 or email to international.health@ua.ac.be (International Health Unit, Department of Epidemiology and Social Medicine, University of Antwerp, Universiteitsplein 1, BE-2610 Antwerp Wilrijk). The fax should state "Urgent Serious Adverse Event" and the study code on the cover page. All other AEs not fulfilling the criteria of immediate reporting must be recorded on the CRF. This AE information will be collected on a regular basis during the clinical trial.

Reporting of adverse events

For all AEs identified, an AE form is completed. For each possible AE identified and considered as serious, an SAE event notification form is completed.

A severity grading scale, based on toxicity grading scales developed by the WHO and the National Institutes of Health, Division of Microbiology and Infectious Diseases, is used to grade severity of all symptoms, physical examination findings, and hemoglobin results (Guidelines for Grading Patient Symptoms, signs and laboratory findings). Any new event, or an event present at baseline that is increasing in severity, will be considered as an AE.

Length of follow-up for adverse events

For AEs presenting during the study, a patient still experiencing an AE at the end of a follow-up (that is at day 42 or 28) will be managed as follows:

- If the AE has been detected and reported before the last visit and
    - it is mild (grade 1), the patient will be managed according to good medical practice and the active follow-up will be stopped. The end date for the AE will be recorded as day 28.
    - its grade is more than 1, the patient will be followed until the AE resolves, improves, or stabilizes.
- If the AE is new, the AE will be reported and the patient will be followed until the AE resolves, improves, or stabilizes.

For patients classified as clinical treatment failures (ETF/LCF/late parasitological failure (LPF)), formal study follow-up ends when a patient is classified as a treatment failure (ETF or LTF/LPF), and patients should be treated and managed according to good medical practice. Additional follow-up for AEs in patients classified as treatment failures is not typically indicated, unless the AE is serious, or is felt to be probably or definitely related to the study medications.

For patients with SAEs, any patient who experiences an SAE should be followed until it resolves or improves (< grade 1). Although formal study follow-up is typically terminated when a patient is classified as a treatment failure, any patient with severe malaria/danger signs should be followed up to ensure their SAE has resolved/improved.

Laboratory evaluations

Blood samples are properly labeled with patients' initials, site number, study number, the study day and the date the sample is taken. Hematology assessments are performed locally at sites. All laboratory results are reported in Standard International Units or in conventional units. Blood samples collected on filter paper for PCR genotyping will be analyzed at the Institute of Tropical Medicine, Antwerp, Belgium.

Case report form

All data and observations must be initially documented in the CRF or copied from source documents to the CRF (in other words, laboratory data). For this study we will use DATAfax (Datafax Incorporation, Texas, The Unated States of America).

Monitoring and quality assurance

The Sponsor will share monitoring and quality assurance with the Amsterdam Institute for Global Health and Development based in Uganda. An agreement was signed to this effect. The task of the monitor is to verify the best conduct of the study through frequent contacts by phone and in person with the Principal Investigator and site staff, in accordance with the Standard Operating Procedures and Good Clinical Practice, with the purposes of facilitating the work and attaining the objectives of the study. These visits enable the monitor to maintain current, personal knowledge of the study through review of the records, comparison with source documents, observation and discussion of the conduct of the study with the investigator. Each site will be visited at least five times during the conduct of the trial, including a pre-study visit and a close-out visit. At each visit, the monitor carries out at least 30% source data verification on the first phase of the study (pre-RCT phase) and 30% source data verification on the second phase of the study (RCT and post-RCT phase); however, these percentages might be increased in case of serious or systematic findings during the monitoring visits. The investigator shall maintain source documents for each patient in the study, consisting of case and visit notes (hospital or clinic medical records) containing demographic and medical information, laboratory data, and the results of any other tests

or assessments. The data on the CRF shall be either the source or traceable to the source documents in the patient's file. The investigator shall keep one copy of the original informed consent form signed by the parent/ guardian (the other will remain with the parent/guardian). The investigator shall give the monitor, auditors, inspectors, Institutional Review Board, and Ethics Committee full access to all relevant source documents to confirm their consistency with the CRF entries.

Data management

The Infectious Diseases Institute in Uganda is in charge of centralized data management. Clinical data, as explained above, are collected and recorded on appropriate paper CRF. Laboratory data, as requested in the protocol, are registered at the laboratory and recorded onto the CRF. All data are then processed from the CRFs into an electronic database. During the conduct of the study, data are verified and reviewed to produce and maintain high-quality data. All unresolved issues are queried and resolved on a regular basis. Data transfer and handling is done with appropriate security measures and with regard to rights, safety and well-being of trial subjects. A report on data management processes will be produced at study completion. The report will include a full field listing and description of the file structure of the electronic data:

– Reference ranges and units for laboratory data
– A brief description of all programs run on the data
– Level of errors found at each stage of checking the data
– General comments on data quality and significant problems encountered with the data
– A detailed list of any unresolved data queries
– A statement of any queries/errors which have not been corrected on the database
– A statement of the storage location of the electronic database

The statistician will review the database prior to finalization and report on any problem encountered during the analysis.

Statistical analysis

A detailed analysis plan will be drawn up prior to the analysis. The statistical analysis of the study will be performed by the DRC Principal Investigator (PI), Dr Hypolite Muhindo Mavoko, in consultation with statisticians at the Institute of Tropical Medicine and/or University of Antwerp, who will also review the analysis plans and analysis results.

Baseline comparability

Children in the treatment groups in each country will be described separately with respect to baseline characteristics. The clinical importance of any imbalance will be noted, although statistical tests of significance will not be undertaken.

Efficacy analyses

Primary

The primary hypothesis of the study is that the PCR-adjusted efficacy at 28 days of retreatment with the same ACT is at least 90%. This hypothesis will be tested by constructing a two-sided 95% confidence interval for the proportion of children without PCR-adjusted rescue treatment failure. If the 95% confidence interval lies entirely above 90% the hypothesis is accepted.

The confidence interval will be constructed using Wilson's score method, pooling the data from the two countries (that is, ASAQ treatment group in DRC and AL treatment group in Uganda).

In addition, the relative efficacy of the retreatment with the same ACT compared to treatment with quinine + clindamycin and treatment with another ACT will be assessed using log-binomial models [19] with fixed effects for treatment group and country. From this model, the relative risks of treatment success will be estimated together with their 95% confidence interval. Appropriateness of pooling the results over the two countries will be assessed by testing country-by-treatment interactions in this log-binomial model.

For the efficacy analysis, both an intention-to-treat and a per-protocol approach will be adopted, with the intention-to-treat analysis being the primary approach. Rules for inclusion/exclusion of children from the per-protocol population will be specified in advance.

Secondary analyses

Statistical methods for secondary analyses will be described in the data analysis plan.

Every effort will be made to minimize the amount of missing data in the trial. Whenever possible, information on the reason for missing data will be obtained. Sensitivity analyses will be undertaken to assess the robustness of the conclusions to the missing data.

Safety analyses

All non-serious and SAEs will be grouped according to a pre-specified side-effect coding system and tabulated for each treatment group. In contrast to the primary efficacy analysis, actual treatment groups will be assessed.

The number (and percentage) of patients experiencing any AE, SAE, or drug-related SAE will be compared between treatment groups using Fisher's exact test. Safety will be analyzed using the all-patients-treated approach. Data analysis will be primarily performed using STATA statistical software packages (Stata Corp, Lakeway, College Station, Texas, USA). Descriptive

statistics will be used to summarize baseline characteristics of study patients. Efficacy and safety data will be evaluated using a modified intention-to-treat analysis and will only include patients who meet all selection criteria. Categorical variables will be compared between the treatment groups using odds ratio, chi-square tests or Fisher's exact tests, and continuous variables will be compared using *t* tests or non-parametric tests. A *P* value of <0.05 will be considered statistically significant. Estimates of the risk of failure for all primary outcomes will be made using the Kaplan-Meier product limit formula. Patients excluded after enrollment will be censored at the time of their last assessment. Additionally, for genotyping adjusted outcomes, patients with recurrent malaria or recurrent parasitemia due to new infections will be censored. Stratified analysis shall be done for outcomes in patients with recurrent symptomatic (ETF, LCF) and recurrent asymptomatic (LPF) malaria. The number (and percentage) of patients experiencing each AE will be compared between the treatment groups. No formal statistical testing will be undertaken.

Investigator responsibility

The term "investigator" as used in this protocol and on the CRFs refers to the PI or a member of the staff that the investigator designates to perform a certain duty under this protocol. The investigator is ultimately responsible for the conduct of all aspects of the study. For all other relevant investigator responsibilities, see [20].

Administrative procedures

Regulatory authorities and ethical review committee

This protocol has been approved by the Ethical Committee of the University of Antwerp (Reference: UA A11-02), the Uganda National Council for Science and Technology (Reference: HS 1108) and the Ethic Committee of the School of Public Health, University of Kinshasa (Reference: ESP/CE/012B/2012).

Informed consent

All interviews are conducted in the native language of the patients by the study personnel. Consent forms in the local language are provided to the parents or guardians for their review. The parents or guardians are asked to sign (or thumb-print whenever the parents/guardians are illiterate) consent to participate in a research study. The informed consent describes the purpose of the study, the procedures to be followed, and the risks and benefits of participation. If a parent or guardian is unable to read or write, an impartial witness takes part in the informed consent discussion and signs the consent form. Parents or guardians are informed that participation in the study is completely voluntary and that they may withdraw their child from the study at any time without any negative consequences.

Confidentiality and publication of results

All study documents are provided by the investigators and their appointed staff. None of this material may be disclosed to any party not directly involved in the study without written permission from the investigator. Publication policy will be addressed in a separate agreement.

Protocol amendments

Clinical protocol amendments are alterations to a legal document (the clinical protocol) and have the same legal status and must pass through the appropriate steps before being implemented. In general, any change must be prior approved by the Independent Ethics Committee to be effective. Administrative changes need only notification to the Independent Ethics Committee without approval. Any subsequent amendments shall be made available with a new protocol with the amendments incorporated and on separate sheets and must pass through the approval process.

Insurance

A no-fault liability insurance has been taken by the Sponsor and covers both trial sites.

Archiving

The relevant study documents are those documents which individually and collectively permit assessment of the conduct of the trial, the quality of the data produced and the compliance with Good Clinical Practice standards and applicable regulatory requirements. The on-site file of the PI contains all the essential documents as listed in the Sponsor's SOP (Set up and maintenance of the Investigator Trial File). A copy of all source data and CRFs must always be kept on site.

The PI is responsible for ensuring a secure and appropriate location for their file and any other trial-related documentation present at site, as well as for ensuring that only site staff that are competent and delegated to work for the trial has access to the files. All the relevant study documentation present at all partners involved should be retained for a minimum of 5 years and according to the applicable National Legislation. The Sponsor should always be informed prior to destruction of the files. After completion of the study, the investigator's file will remain available for internal audits and/or inspections of regulatory authorities for a period of 20 years, unless otherwise requested by national authorities.

Data safety and monitoring board

An independent DSMB with three members (a pediatrician, a statistician and a malariologist/ epidemiologist) has been

created before field activities started. The DSMB was established for the purpose of providing independent advice on the quality of the data produced and the efficacy and safety of the treatments tested, so contributing to safeguarding the interests of the trial participants. More specifically the DSMB:

- assesses the quality of data, including completeness;
- monitors recruitment figures and loss to follow-up;
- monitors compliance with the protocol by participants and investigators;
- monitors evidence for treatment differences in the main efficacy and safety outcome measures, thus recommending action when/whether the main trial question has been answered;
- monitors evidence for treatment harm (for example, toxicity, SAEs, deaths);
- recommends whether the trial should continue to recruit or follow-up;
- recommends any major changes to the protocol where necessary (for example, changes to recruitment procedures, inclusion criteria, endpoints, data collection, and so forth);
- advises on and/or endorsing any major protocol modifications suggested by the Trial Steering Committee (for example, changes to the inclusion criteria, endpoints, data collection, and so forth);
- monitors planned sample size assumptions;
- suggests additional data analyses;
- assesses the impact and relevance of any external evidence provided;
- monitors compliance with previous DSMB recommendations;
- considers the ethical implications of any recommendations made by the DSMB.

In this respect, the DSMB has the possibility to monitor the safety of the treatment on a continuous basis and possibly stop the study if any major safety concern appears. A DSMB charter, where the relevant terms of reference are clearly defined, has been signed by each member of the Committee.

Ethical issues

The investigators agreed to conduct the present study in full agreement with the principles of the "Declaration of Helsinki" and subsequent relevant amendments. All research activities are run in accordance with the standards and codes of conduct accepted by the International Conference on Harmonisation guidelines.

AL and ASAQ have been successfully used for the treatment of falciparum malaria in Phase III studies and are both widely used to treat uncomplicated malaria in Africa. The combinations are well tolerated. In DRC, quinine + clindamycin is the second-line treatment for malaria. Uganda has not yet adjusted a treatment policy. The treatment is cheap, widely available and generally considered to be effective.

Blood is collected for the hematology, biochemistry, blood slides, and later genotyping and other tests that the clinician may request. The amount collected for hemotology and biochemistry is around 3 ml (venous) while for the other tests the amount is a few drops collected by fingerprick. Residual serum will is separated, stored and transported at -70°C to be assayed by ELISA and fluorescence-activated cell sorting for malarial antibody quantification (described in an independent protocol after the operational part of the study is finished).

Trial status

Active recruitment.

Additional files

Additional file 1: **Follow-up chart for the pre-randomized controlled trial phase.**

Additional file 2: **Follow-up chart for the randomized controlled trial phase (and post-randomized controlled trial).**

Abbreviations

ACPR: Adequate clinical and parasitological response; ACT: Artemisinin-based combination therapy; AE: Adverse event; AL: Artemether-lumefantrine; ASAQ: Artesunate-amodiaquine; CRF: Case report form; DSMB: Data Safety and Monitoring Board; DRC: Democratic Republic of Congo; ELISA: Enzyme-linked immunosorbent assay; ETF: Early treatment failure; LCF: Late clinical failure; LPF: Late parasitological failure; LTF: Late treatment failure; PCR: Polymerase chain reaction; pfmdr: *Plasmodium falciparum* multidrug resistance; PI: Principal Investigator; RCT: Randomized clinical trial; SAE: Serious adverse event; TMG: Trial Management Group; WHO: World Health Organization.

Competing interests

The authors declare that they have no competing interests.

Authors' contributions

HMM and JPVG have written and designed the study protocol. CN, HT, UDA, MPG and PL have commented and delivered expert opinion on the protocol. HMM and CN are site principal investigators, respectively, in DRC and Uganda. JPVG is coordinating the trial on behalf of the sponsor. All authors read and approved the final manuscript.

Acknowledgements

The authors thank all co-funders of this study: EDCTP (Grant reference MS.2010.10800.004), FWO (Grant reference G.078.11), VLIR-UOS (Grant reference ZRDC2012MP076 and ZEIN2013PR401) and the Belgian Technical Cooperation in DRC (Grant reference FEE/06/12). We acknowledge the contribution of Raffaella Ravinetto to the conception of the idea. We would like to thank the monitoring team: Elske Straatsma, Joachim Doua, Caroline Bako and Sophie Nabasirye. We acknowledge the reliability of the study staff in both sites. Many thanks to the participants and their parents, as well as the managing boards of health facilities where the study is conducted.

Author details

[1]Département de Médecine Tropicale, Faculté de Médecine, Université de Kinshasa, B.P. 747 Kin XI, République Démocratique du Congo. [2]Epicentre Mbarara Reasearch Base, P. O. Box 930, Mbarara, Uganda. [3]Centre Muraz/ Institut de Recherche en Sciences de la Santé, P. O. Box 545, Bobo Dioulasso, Burkina Faso. [4]Institute of Tropical Medicine, Nationalestraat 155, B-2000,

Antwerp, Belgium. [5]Medical Research Council, the Gambia Unit, P. O. Box 273, Banjul, The Gambia. [6]Academic Medical Center, University of Amsterdam, Meibergdreef 9, 1105 AZ, Amsterdam, Netherlands. [7]International Health Unit, University of Antwerp, Campus Drie Eiken, Universiteitsplein 1, 2610, Wilrijk, Belgium.

Received: 20 April 2013 Accepted: 11 September 2013
Published: 23 September 2013

References

1. Hay S, Guerra C, Tatem A, Noor A, Snow R: **The global distribution and population at risk of malaria: past, present, and future.** *Lancet Infect Dis* 2004, **4**:327–336.
2. White NJ, Olliaro P: **Artemisinin and derivatives in the treatment of uncomplicated malaria.** *Med Trop* 1998, **58**(Suppl 3):54–56.
3. Ndiaye JL, Randrianarivelojosia M, Sagara I, Brasseur P, Ndiaye I, Faye B, Randrianasolo L, Ratsimbasoa A, Forlemu D, Moor VA, Traore A, Dicko Y, Dara N, Lameyre V, Diallo M, Djimde A, Same-Ekobo A, Gaye O: **Randomized, multicentre assessment of the efficacy and safety of ASAQ - a fixed-dose artesunate-amodiaquine combination therapy in the treatment of uncomplicated *Plasmodium falciparum* malaria.** *Malar J* 2009, **8**:125.
4. Swarthout TD, van den Broek IV, Kayembe G, Montgomery J, Pota H, Roper C: **Artesunate + amodiaquine and artesunate + sulphadoxine-pyrimethamine for treatment of uncomplicated malaria in Democratic Republic of Congo: a clinical trial with determination of sulphadoxine and pyrimethamine-resistant haplotypes.** *Trop Med Int Health* 2006, **11**:1503–1511.
5. Sirima SB, Tiono AB, Gansané A, Diarra A, Ouédraogo A, Konaté AT, Kiechel JR, Morgan CC, Olliaro PL, Taylor WR: **The efficacy and safety of a new fixed-dose combination of amodiaquine and artesunate in young African children with acute uncomplicated *Plasmodium falciparum*.** *Malar J* 2009, **8**:48.
6. Zwang J, Olliaro P, Barennes H, Bonnet M, Brasseur P, Bukirwa H, Cohuet S, D'Alessandro U, Djimdé A, Karema C, Guthmann JP, Hamour S, Ndiaye JL, Mårtensson A, Rwagacondo C, Sagara I, Same-Ekobo A, Sirima SB, van den Broek I, Yeka A, Taylor WR, Dorsey G, Randrianarivelojosia M: **Efficacy of artesunate-amodiaquine for treating uncomplicated falciparum malaria in sub-Saharan Africa: a multi-centre analysis.** *Malar J* 2009, **8**:203.
7. Byakika-Kibwika P, Lamorde M, Mayanja-Kizza H, Merry C, Colebunders B, van Geertruyden JP: **Update on the efficacy, effectiveness and safety of artemether-lumefantrine combination therapy for treatment of uncomplicated malaria.** *Ther Clin Risk Manag* 2010, **6**:11–20.
8. de Vries PJ, Bich NN, Van Thien H, Hung LN, Anh TK, Kager PA, Heisterkamp SH: **Combinations of artemisinin and quinine for uncomplicated falciparum malaria: efficacy and pharmacodynamics.** *Antimicrob Agents Chemother* 2000, **44**:1302–1308.
9. Yeka A, Achan J, D'Alessandro U, Talisuna AO: **Quinine monotherapy for treating uncomplicated malaria in the era of artemisinin-based combination therapy: an appropriate public health policy?** *Lancet Infect Dis* 2009, **9**:448–452.
10. Bloland P: *Drug Resistance in Malaria. A Background Document for the WHO Global Strategy for Containment of Antimicrobial Resistance.* Geneva: WHO/CDS/CSR/DRS; 2001.
11. Fungladda W, Honrado ER, Thimasarn K, Kitayaporn D, Karbwang J, Kamolratanakul P, Masngammueng R: **Compliance with artesunate and quinine + tetracycline treatment of uncomplicated falciparum malaria in Thailand.** *Bull World Health Organ* 1998, **76**(Suppl 1):59–66.
12. Achan J, Tibenderana JK, Kyabayinze D, Mangen FW, Kamya MR, Dorsey G, D'Alessandro U, Rosenthal P, Talisuna AO: **Effectiveness of quinine versus artemether-lumefantrine for treating uncomplicated falciparum malaria in Ugandan children: randomised trial.** *BMJ* 2009, **339**:b2763.
13. WHO: *Guidelines for the Treatment of Malaria.* Geneva: World Health Organization; 2010.
14. Obonyo CO, Juma EA: **Clindamycin plus quinine for treating uncomplicated falciparum malaria: a systematic review and meta-analysis.** *Malar J* 2012, **11**:2.
15. WHO: **Severe falciparum malaria.** *Trans R Soc Trop Med Hyg* 2000, **94**:1–90.
16. WHO: *Assessment and Monitoring of Antimalarial Drug Efficacy for the Treatment of Uncomplicated falciparum Malaria.* Geneva: World Health Organization; 2003.
17. Bonnet M, Broek I, van Herp M, Urrutia PP, van Overmeir C, Kyomuhendo J, Ndosimao CN, Ashley E, Guthmann JP: **Varying efficacy of artesunate +amodiaquine and artesunate+sulphadoxine-pyrimethamine for the treatment of uncomplicated falciparum malaria in the Democratic Republic of Congo: a report of two in-vivo studies.** *Malar J* 2009, **8**:192.
18. Bassat Q, Mulenga M, Tinto H, Piola P, Borrmann S, Menéndez C, Nambozi M, Valéa I, Nabasumba C, Sasi P, Bacchieri A, Corsi M, Ubben D, Talisuna A, D'Alessandro U: **Dihydroartemisinin-piperaquine and artemether-lumefantrine for treating uncomplicated malaria in African children: a randomised, non-inferiority trial.** *PLoS One* 2009, **4**:e7871.
19. McNutt LA, Wu C, Xue X, Hafner JP: **Estimating the relative risk in cohort studies and clinical trials of common outcomes.** *Am J Epidemiol* 2003, **157**:940–943.
20. EMEA: *Guideline for Good Clinical Practice ICH Topic E6 (R1).* London: European Medicines Agency CPMP/ICH/135/95; 2002.

doi:10.1186/1745-6215-14-307
Cite this article as: Muhindo Mavoko *et al.*: Impact of retreatment with an artemisinin-based combination on malaria incidence and its potential selection of resistant strains: study protocol for a randomized controlled clinical trial. *Trials* 2013 **14**:307.

Submit your next manuscript to BioMed Central and take full advantage of:

- Convenient online submission
- Thorough peer review
- No space constraints or color figure charges
- Immediate publication on acceptance
- Inclusion in PubMed, CAS, Scopus and Google Scholar
- Research which is freely available for redistribution

Submit your manuscript at
www.biomedcentral.com/submit

BioMed Central
