# Supplementary material for: Uncomplicated Clinical Malaria Features, the Efficacy of Artesunate-Amodiaquine and Their Relation with Multiplicity of Infection in the Democratic Republic of Congo
Source: PLoS One. 2016 Jun 9;11(6):e0157074. doi: 10.1371/journal.pone.0157074 (PMC4900589; doi:10.1371/journal.pone.0157074)
Supplement: S2 Protocol — (PDF) [file pone.0157074.s003.pdf]

A Randomized Clinical Trial to measure the impact of retreatment with an artemisinin-based combination on malaria incidence and its potential selection of resistant strains

Code : UA-IHU- 2010-01
Version : protocol 1.3.
Date: 1 September 2011Sponsor
University of Antwerp
Faculty of Medicine,
Antwerp University,
Universiteitsplein 1
BE-2610 Antwerpen (Wilrijk)

Study director (sponsor)
Prof. Jean-Pierre Van geertruyden MD PhD
Head **International Health** Unit,
Department of Epidemiology and Social Medicine,
Faculty of Medicine, Antwerp University,
Universiteitsplein 1
BE-2610 Antwerpen (Wilrijk)
Tel: ++ 32 32652528
Mobile ++ 32 484618493
Skype: jean.pierre.van.geertruyden

Country Director (RDC)
Professeur Pascal Lutumba
Université de Kinshasa
Faculté de Médecine
B.P. 747 Kinshasa XI
République Démocratique du Congo

Country Director (Uganda)
Professeur Moses Kamya
Infectious Disease Institute
University Makarere
2190 Kampala Place, Dulles,
VA 20189-2190
Uganda

	Final
**Date**	1 September 2011
**Version**	One
**Title**	The impact of retreatment with an artemisinin-based combination on malaria incidence and its potential selection of resistant strains
**Protocol Id.**	QUINACT
**Phase**	IIIb
**ClinTrialsGov number**	NCT01374581
**Country**	DRCongo, Uganda.
**Sites :**	• Centre de Santé Lisungi,<br>Route Kimwenza n°23,<br>Commune de Mont-Ngafula,<br>Kinshasa.<br>Telephone: +243 816696000<br><br>• Kazo Health centre IV<br>P.O Box 5 Rushere<br>Kiruhura<br>Tel no: +256 782 311 919.
**Study drugs**	Artesunate-amodiaquine (Co-arsucam®, Sanofi-Aventis)<br>Quinine (Quinimax®, Sanofi-Aventis) + clindamycine<br>Artemether+Lumefantrine (Coartem®, Novartis)
**Sponsor:**	University of Antwerp, Antwerp, Belgium<br>Prof. Jean-Pierre Van geertruyden
**Monitoring, training, safety reporting:**	**International Health** Unit<br>Department of Epidemiology and Social Medicine,<br>Faculty of Medicine, Antwerp University,<br>Universiteitsplein 1<br>BE-2610 Antwerpen (Wilrijk)<br>Tel: ++ 32 32652528<br>Mobile ++ 32 484618493<br>Skype: jean.pierre.van.geertruyden<br>&<br>The Amsterdam Institute for Global Health and Development (AIGHD)<br>Plot 537, Tufnell Drive, Mulago Hill<br>P.O. Box 70345<br>Kampala, Uganda
**Country Director (DRC)**	Prof. Pascal Lutumba
**PI (RDC)**	Dr. Hypolite Muhindo
**Country Director (Uganda)**	Prof. Moses Kamya
**PI**	Dr. Carolyn Nabasumba
**Address of the sponsor:**	University of Antwerp, Faculty of Medicine,<br>Antwerp University, Universiteitsplein 1, BE-2610 Antwerpen (Wilrijk)
**Telephone:**	Tel: ++ 32 32652528
**Email:**	Jean-pierre.vangeertruyden@ua.ac.be
**Skype**	jean.pierre.van.geertruyden

PROTOCOL APPROVAL

**Approval**	**Signature**	**Date**
Dr Hypolite Muhindo Mavoko	________________________________<br>Principal Investigator DRCongo	____/____/___
Dr Carolyn Nabasumba	______________________________________<br>Principal Investigator Uganda	___/____/___
Prof. Jean-Pierre Van geertruyden	___________________________<br>Sponsor Study Director	___/____/___
Prof. Pascal Lutumba	_______________________________________<br>Country Study Director RDCongo	___/____/___
Prof. Moses Kamya	____<br>Country Study Director Uganda	___/____/___
Prof. Alain Verschoren	______________________________________<br>Vice Chancelor University of Antwerp	____/____/___

Other scientific collaborators who have read and commented on the protocol are

- Prof. Umberto d'Alessandro, Institute of Tropical Medicine, Belgium
- Dr. Halidou Tinto, Centre Muraz, Burkina Faso
- Martin Grobusch, Amsterdam Medical Centre, Netherlands
- Frank Cobelens , Amsterdam Medical Centre, Netherlands
- Raffaella Ravinetto, Institute of Tropical Medicine, Belgium
- Celine Schurmans, Clinical Trials Unit, Institute of Tropical Medicine, Belgium
- Joris Menten, Clinical Trials Unit, Institute of Tropical Medicine, Belgium
- Prof. Robert Colebunders, University of Antwerp, Belgium

STUDY ACKNOWLEDGEMENT/CONFIDENTIALITY

By signing this protocol, the Principal Investigator(s) acknowledges and agrees:
The protocol contains all necessary information for conducting study. The Principal Investigator will conduct this study as detailed herein and will make every reasonable effort to complete the study within the time designated. The Principal Investigator commits to carry out the study in compliance with the protocol, amendments, current SOP's and other study-related documents provided by the Sponsor, and in compliance with applicable ethical, GCP and regulatory requirements.

The protocol and all relevant information on the study drugs relating to pre-clinical, clinical and post-marketing experience, provided by the drugs' manufacturers and by the Sponsor, will be made available to all physicians, nurses and other personnel who participate in conducting this study. The Investigator will use this material for their training so that they are fully informed regarding the drugs and the conduct of the study.

This document contains information that is privileged or confidential. As such, it may not be disclosed unless specific permission is granted in writing by the University of Antwerp, Antwerp, Belgium, or such disclosure is required by national or other laws or regulations. In any event, persons to whom the information is disclosed must be informed that the information is privileged or confidential and may not be further disclosed by them. These restrictions on disclosure will apply equally to all future information supplied which is indicated as privileged or confidential.

The University of Antwerp, Belgium, , the Infectious Disease Institute, Uganda, the Amsterdam Medical Centre, Netherlands and the Department of Tropical Medicine, University of Kinshasa, DRC will have access to any source documents from which Case Report Form information may have been generated. The Case Report Forms and any other data pertinent to this study are the joint property of the University of Antwerp, Belgium, , the Department of Tropical Medicine, University of Kinshasa, DRC and the Infectious Disease Institute, Makerere University, Uganda who may utilise the data jointly in various ways, such as publication of the results of this study. No partners will use such data independently, without the previous agreement of the other partners.

The conduct and results of this study will be kept confidential until completion, unless an interim publication or presentation is agreed upon among the five partners.

Name **Signature** **Date**

Dr Hypolite Muhindo Mavoko _______________________________ ____/____/___
Principal Investigator DRCongo

Dr Carolyn Nabasumba _______________________________ ____/____/___
Principal Investigator Uganda

Signing this document I commit to carry out the trial according to the protocol and to all the applicable ethical and regulatory requirements. I also declare to have read the paragraph relevant to study acknowledgement and confidentiality and authorise the University of Antwerp, Belgium to record my data on a computerised archive containing all the data pertinent to the study.

PROTOCOL APPROVAL 3
STUDY ACKNOWLEDGEMENT/CONFIDENTIALITY 4
SYNOPSIS 7
1. BACKGROUND AND RATIONALE 10
2. TRIAL OBJECTIVES AND PURPOSE 10
3. DRUGS TO BE TESTED 11
3.1. Quinine + clindamycin. 11
3.2. Artemether –lumefantrine (AL) 12
3.3. Amodiaquine artesunate 12
4. STUDY DESIGN 12
4.1. Study phases 12
4.2. Patient selection 14
Exclusion criteria (RCT Phase) 15
4.3. Endpoints 15
4.3.1. Primary Endpoint 15
4.3.2. Secondary Efficacy Endpoints 16
4.3.3. Safety Endpoints 16
4.4. PCR analysis 16
4.4.1. Genotyping 17
4.4.2. Molecular markers **Error! Bookmark not defined.**
4.4.3. Interpretation of PCR results 17
4.5. Frozen Plasma samples 17
4.6. Sample size 17
4.7. Study Procedures and Duration of patient follow up 18
4.7.1. Pre-RCT phase: First line treatment 18
4.7.2. RCT-phase 19
4.7.3. Post RCT-Phase- Further rescue treatment 22
4.8. Randomisation and treatment allocation 22
4.9. Treatment administration 22
5. CONCOMITANT THERAPIES 23
5.1. Disallowed Concomitant Drug Therapies during the active follow ups 23
5.2 Allowed Concomitant Drug Therapies 23
5.3 Special Conditions 23
5.4 Rescue Treatments 23
6. PATIENT WITHDRAWAL CRITERIA 23
7. PROTOCOL VIOLATIONS 23
8. SAFETY VARIABLES 23
8.1. Adverse Events 23
8.1.1. Definition of an adverse event 23
8.1.2. Severity, relationship of event to study drug, and outcome 24
8.1.3. Definition of a serious adverse event 25
8.1.4. Reporting of adverse events 25
8.1.5. Length of follow-up for adverse events 26
8.2. Laboratory Evaluations 26
9. CASE REPORT FORM (CRF) 26

10. MONITORING AND QUALITY ASSURANCE 27
11. DATA MANAGEMENT 27
12. STATISTICAL ANALYSIS 28
1) Baseline comparability 28
2) Efficacy Analyses 28
3) Safety Analyses 28
13. INVESTIGATOR RESPONSIBILITY 29
14. ADMINISTRATIVE PROCEDURES 29
14.1. Regulatory Authorities and Ethical Review Committee 29
14.2. Informed Consent 29
14.3. Confidentiality and Publication of Results 29
14.4. Protocol Amendments 29
14.5. Insurance 29
14.6. Archiving 30
15. DATA SAFETY AND MONITORING BOARD 30
16. ETHICAL ISSUES 30
17. REFERENCES 31
APPENDICES 32
APPENDIX I 32
APPENDIX II* 37
APPENDIX III 38
APPENDIX IV 40
APPENDIX V 44
APPENDIX VI. PARTICIPANT SELECTION AND ENROLLMENT 45
APPENDIX VII. CRITICAL STEPS. 46

SYNOPSIS

Title

The impact of retreatment with an artemisinin-based combination on malaria incidence and its potential selection of resistant strains

Methodology

This is a study incorporating a bi-centre, phase IIIb, randomized, open label, 3-arm trial. It will be performed in three steps and there will be informed consent for the first two steps. In the first step, each patient will be passively followed up for 42 days without systematic screening. Throughout the study, any failures appearing before 14 days of any treatment will be treated according the National guidelines. If, during the follow up, the patient experiences a second uncomplicated malaria episode, s/he will be randomized to

- Quinine + clindamycin, the recommended rescue treatment,
- the same first line ACT, a common practice fielding peripheral health centres
- a second ACT as recommended by WHO

and actively followed up for 28 additional days. If, during the active follow up, the patient experiences a new uncomplicated malaria episode, s/he will be retreated with the recommended first line ACT and actively followed up for 28 additional days. The same methodology will be followed for each subsequent failure up to not more than 3 post RCT treatment courses. In any case, all patients without an infection at day 28, will be classified as treatment successes and will leave the study.

Study Hypothesis

Re-treatment with the first line ACT treatment beyond 14 days is as efficacious as any other rescue treatment, without the risk of selecting drug resistant strains

Clinical Trial Objectives

Efficacy

The primary objective is:

(a) to show that, in children aged 12 to 59 months with recurrent uncomplicated *P. falciparum* malaria within 42 days of treatment with an artemisinin-based combination therapy (ASAQ in DRC or AL in Ug), the PCR adjusted efficacy at 28 days after re-treatment with the same artemisinin-based combination therapy is at least 90% and

(b) to estimate the relative efficacy of re-treatment with the same artemisinin-based combination compared to treatment with quinine + clindamycin and treatment with another artemisinin-based combination therapy (ASAQ after first line AL treatment or AL after first line ASAQ treatment).

Secondary objectives are:

1. To evaluate the PCR-unadjusted efficacy at 28 days of re-treatment with the same artemisinin-based combination therapy and to compare it to treatment with quinine + clindamycin and treatment with another artemisinin-based combination therapy (ASAQ after first line AL treatment or AL after first line ASAQ treatment).
2. To evaluate and compare the efficacy of AL, ASAQ and quinine + clindamycin as rescue treatment for a recurrent *P. falciparum* malaria episode occurring 2 weeks after the administration of the first line treatment, with and without PCR adjustment.
3. To evaluate and compare the 42 days clinical efficacy of AL (Ug) and ASAQ (RDC) for the first line treatment of uncomplicated *P. falciparum* malaria, with and without PCR adjustment.
4. To evaluate and compare the efficacy of the different rescue treatment regimens in terms of fever clearance time, asexual parasite clearance time, gametocytaemia at day 7, 14, 21 and 28, and, Hb changes between day 0 and days 14 and 28.

Additional objectives are:

1. To evaluate the selection of *Plasmodium falciparum* pfmdr1 alleles following therapy with quinine + clindamycin, AL and ASAQ.
2. To assess epidemiological, parasitological and host related predictors for recurrent malaria infections*

*adjacent study that will be developed in a separate nested study protocol.

Safety

To evaluate the safety and tolerability of AL, ASAQ and quinine + clindamycin when used as rescue treatments.

Study endpoints

Primary Efficacy Endpoint
PCR adjusted efficacy at 28 days: the proportion of children with PCR adequate clinical and parasitological response at day 28 (ACPR28A): all early failures before day 7 plus the recurrent parasitaemias detected later and classified by genotyping as recrudescence.
The TF is defined according to the WHO criteria (WHO 2003) as the sum of early* and late** treatment failures.
Early (ETF) and late treatment failures (LTF) are defined according to the WHO criteria (WHO 2003). Total treatment failure (TF) is the sum of ETF and LTF.

Secondary Efficacy Endpoints
1. PCR unadjusted efficacy at 28 days: the proportion of children with (PCR non adjusted) treatment failure (ACPR28U): all treatment failures detected during the active follow up, regardless of genotyping.
2. 42 days clinical treatment failure: all clinical treatment failures detected during the 42 days follow up for the first line treatment, with and without PCR adjustment. As no active monitoring of parasitaemia after drug administration is planned this includes ETF and LCF following WHO criteria.
3. Fever clearance time (FCT): Fever clearance time is defined as the time (in days) from the time of treatment to the first two consecutive measurements on 2 different days of tympanictemperature below 38.0°C.
4. Asexual parasite clearance time (PCT): Asexual parasite clearance time is defined as the time (in days) from time of randomization to 2 consecutive negative blood slides (collected at different days). The time to the event will be taken as the time to the first negative slide.
5. Gametocytaemia (prevalence and density) at day 7, 14, 21 and 28 after treatment.
6. Hb changes between day 0 and days 14 and 28.

Tertiary Efficacy Endpoints will be defined in the substudy protocols

Safety Endpoints: Subjects will be monitored throughout the study for possible development of adverse events. All adverse events will be recorded on the specific form in the CRF. Vital signs, and haematology will be monitored and changes in relevant laboratory parameters will be assessed

Number of Subjects
We will recruit 310 patients in each site (124 on ASAQ and AL each, and 62 on quinine + clindamycin). The 2:2:1 allocation ration is chosen to reduce the number of patients randomized to the more demanding quinine + clindamycin treatment regimen, 620 in total.

Countries
2 African countries: DRCongo and Uganda

Study Center(s)
2 sites: Lisungi health Centre, DRCongo & Mbarara HC, Uganda

Follow-up Chart pre-RCT phase: 1st line treatment (ASAQ in DRC and AL in Uganda)

Day	0	1	2		14[1]		28[1]		42[1]	Any other day[1]
History (symptoms)	X				X		X		X	X
Examination (clinical)	X	X	X		X*		X*		X*	X
Temperature	X	X	X		X*		X*		X*	X
Rapid test	X									
Blood film	X		X*		X*		X*		X*	X
Filter paper PCR	X		X*		X*		X*		X*	X
Informed consent	X									
Plasma sample	X									
Haematology	X									
Treatment	X	X	X							
Adverse events	X	X	X		X		*X*		*X*	*X*
Concomitant medications	X	X	X		X		*X*		*X*	*X*

X = perform this task; * only if symptoms indicate a possible clinical treatment failure
[1] For a clinical treatment failure see day 0 RCT phase

Follow up Chart RCT phase: Rescue treatment (ASAQ, AL or QN) [1]
Flow chart

Day	0	1	2	3	4-6	7	14[1]	21[1]	28[1]	Any other day[1]
History (symptoms)	X					X	X	X	X	X
Examination (clinical)	X	X	X	X		X	X	X	X	X
Temperature	X	X	X	X		X	X	X	X	X
Blood film	X		X	X		X	X	X	X	X
Filter paper PCR	X		X	X		X	X	X	X	X
Informed consent	X									
Haematology	X						X		X	X
Plasma sample	X									
Treatment	X	X	X	X[1]	X[1]					
Adverse events	X	X	X	X	X[1]	X	X	X	*X*	*X*
Concomitant medications	X	X	X	X	X[1]	X	X	X	*X*	*X*

X[1] = quinine + clindamycin treatment administration; only for RCT phase

[1] For a treatment failure see day 0 Post-RCT phase

All study medications for patients randomised to ACT's shall be administered under direct observation. Study medications for patients randomised to quinine + clindamycin shall be administered under direct observation on the first 3 days (9 doses) and monitored closely to warrant treatment adherence. For the remaining quinine + clindamycin doses, the two doses for the day shall be administered in the clinic under direct supervision by the study nurse and the third dose for the day shall be administered by the parents or guardians at home. Patients will have the option to be admitted for observation and study drug administration or to commute from home. Parents/guardians will be encouraged to return to the clinic for follow up assessments on days 3, 7, 14, 21, 28 and on any unscheduled day if the child is not well. ***Study Duration***

Calendar of activity.

**YEAR**	**2011**				**2012**				**2013**	
**QUARTERS**	**1**	**2**	**3**	**4**	**1**	**2**	**3**	**4**	**1**	**2**
**Preparatory phase**										
Meeting of research groups (coordination)										
Updating finalizing of detailed protocol (after EDCTP approval)										
e-CRF and data base design										
Ethical Committee approval of participating sites										
Information/agreement with study populations										
Training in GCP										
Training data entry and data management										
**Implementation phase**										
Coordination meetings (teleconferences every 3 months)										
Monitoring initiation visit										
Recruitment of patients										
Monitoring visits (>3 times per site)										
Closure monitoring visit										
Genotyping blood samples										
Data Analysis										
Publications										

Institutions involved

- Department of tropical medicine, University of Kinshasa, RDCongo
- Institute of Infectious Diseases, Kampala, Uganda
- International Health Unit, University of Antwerp, Belgium
- Institute of Tropical Medicine, Antwerp, Belgium
- AMC-CPCD/AIGHD Uganda
- Amsterdam Medical Centre, Amsterdam, Holland

1. BACKGROUND and RATIONALE

Malaria remains one of the great infectious killers in Africa. An estimated 300 to 500 million cases occur each year, causing 1.5 to 2.7 million deaths, primarily in children under the age of five (1). The reduction of malaria-associated morbidity and mortality relies largely on chemotherapy. Considering these facts, for the foreseeable future, the major intervention available for the control of malaria (and a key Roll Back Malaria priority) remains the prompt treatment of symptomatic malaria with effective therapy. However, the success of this strategy has been greatly affected by the increasing resistance of malaria parasites to available drugs. Increased progression of disease from uncomplicated to complicated forms, and increased resultant mortality (2). Choices of the best treatment for uncomplicated malaria in Africa have become increasingly complex.

Following the WHO guidelines, most African countries have already opted for artemisinin-based combination therapy (ACT). Several clinical trials on amodiaquine-artesunate (ASAQ), an ACT, completed in Africa have shown an efficacy > 90% (3-5). Furthermore, after PCR analysis, over 75 % of ASAQ & AL treatment failures have been classified as new infections, while recrudescences have low parasite densities (6). ASAQ is safe and easy administered, with a good treatment adherence (3-5). Therefore, effectiveness may be close to efficacy . ASAQ has now been developed as fixed-dose combination and registered. The Democratic Republic of Congo (DRC) has also chosen artesunate-amodiaquine (ASAQ) as first-line treatment for uncomplicated malaria.
Efficacy of the 6 dose regimen of AL has been demonstrated in semi-immune and non-immune populations in Asia and Africa to be consistently greater than 95%, with rapid parasite and symptom clearance and significant gametocytocidal effect (7).In Uganda, AL has already been chosen as first-line treatment for uncomplicated malaria.

In DRC and Uganda, quinine is the rescue treatment for malaria. It is cheap, widely available and generally considered to be effective but is not popular due to its side effects. Quinine has a very short half-life, therefore repeated dosing is required. In an efficacy study of quinine and artemisinin for uncomplicated malaria in Vietnam, recrudescence rates were 16% after 7 days of quinine monotherapy (8). In studies conducted in Gabon, *Plasmodium falciparum in vitro* sensitivity to quinine was high and had not changed over the past decade (9). Although quinine monotherapy shows high efficacy in the setting of clinical trials, it has considerable disadvantages, mainly because of its poor tolerability and the prolonged treatment course. Poor adherence carries a high risk of treatment failure, particularly because quinine causes a syndrome of adverse effects known as cinchonism that includes primarily tinnitus, nausea, and vertigo. Other reported side effects are high tone hearing impairment, dizziness, hypotension as well as headache and visual disturbances (10). As result of these side effects, some studies have reported poor compliance to treatment. A randomized trial in Thailand reported 71% adherence Such poor adherence to the 7-days regimen is associated with a high risk of treatment failure (11), which can contribute to the development and spread of resistance (10). Furthermore, in current practices patients are often re-treated with the recommended first line drug, i.e. ASAQ.
As quinine is effective against all species of malaria including chloroquine-resistant strains of *P. falciparum,* it is widely used for the treatment of severe malaria. Therefore, it should be protected from resistance by a rational use, as its effectiveness in uncomplicated malaria is lower than ACT (12).

Rationale

Considering the facts that: (i) over >75% of treatment failure to ASAQ or AL are new infections, (ii) parasite density is low in case of recrudescence occurring from day 14 onwards and (ii) in real-life situations patients are re-treated with the same first line drug, there is the need to assess the role of the first line treatment as rescue treatments. This efficacy will be compared to quinine + clindamycin and another ACT treatment in line with the WHO guideline (15) to provide clear guidelines. We hypothesize that re-treatment with the first line ACT treatment beyond 14 days is as efficacious as any other rescue treatment, without the risk of selecting drug resistant strains. Furthermore, a prolonged follow up will allow the assessment of the epidemiological, parasite related risk factors for repeated malaria infection and to collect samples for immunological risk factors for repeated malaria attacks.

2. TRIAL OBJECTIVES AND PURPOSE

Efficacy

The primary objective is:

(a) to show that, in children aged 12 to 59 months with recurrent uncomplicated *P. falciparum* malaria within 42 days of treatment with an artemisinin-based combination therapy (ASAQ in DRC or AL in Ug), the PCR adjusted efficacy at 28 days after re-treatment with the same artemisinin-based combination therapy is at least 90% and

(b) to estimate the relative efficacy of re-treatment with the same artemisinin-based combination compared to treatment with quinine + clindamycin and treatment with another artemisinin-based combination therapy (ASAQ after first line AL treatment or AL after first line ASAQ treatment).

Secondary objectives are:
1. To evaluate the PCR-unadjusted efficacy at 28 days of re-treatment with the same artemisinin-based combination therapy and to compare it to treatment with quinine + clindamycin and treatment with another artemisinin-based combination therapy (ASAQ after first line AL treatment or AL after first line ASAQ treatment).
2. To evaluate and compare the efficacy of AL, ASAQ and quinine + clindamycin as rescue treatment for a recurrent *P. falciparum* malaria episode occurring 2 weeks after the administration of the first line treatment, with and without PCR adjustment.
3. To evaluate and compare the 42 days clinical efficacy of AL (Ug) and ASAQ (RDC) for the first line treatment of uncomplicated *P. falciparum* malaria, with and without PCR adjustment.
4. To evaluate and compare the efficacy of the different rescue treatment regimens in terms of fever clearance time, asexual parasite clearance time, gametocytaemia at day 7, 14, 21 and 28, and, Hb changes between day 0 and days 14 and 28.

Additional objectives are:

1. To evaluate the selection of *Plasmodium falciparum pfmdr1* alleles following therapy with quinine + clindamycin, AL and ASAQ.
2. To assess epidemiological, parasitological and host related predictors for recurrent malaria infections*
*adjacent study that will be developed in a separate nested study protocol.

Safety
To evaluate the safety and tolerability of AL, ASAQ and quinine + clindamycin when used as rescue treatments.

3. Drugs to be tested

3.1. Quinine + clindamycin.

Quinine is an alkaloids from the bark of the cinchona tree that still constitutes one of the major components of the antimalarial pharmacopeia, as they have for over three centuries. Quinine is widely used for the treatment of severe malaria. It is effective against all species of malaria including chloroquine-resistant strains of *P. falciparum*. Quinine belongs to the aryl amino alcohol group of drugs. It is a cinchona alkaloid that has a rapid schizonticidal action on the intra-erythrocytic parasites and is also gametocytocidal for *P. vivax* and *P. malariae* but not for *P. falciparum*.
In DRCongo, quinine + clindamycin is the second line treatment for malaria. In Uganda, quinine is the rescue but a combination with clindamycin might be expected In the near future. Quinine is cheap, widely available and generally considered to be effective though it is not popular due to the unwanted side effects. Quinine has a very short half-life so that repeated daily dosing is required. In an efficacy study of quinine and artemisinin for uncomplicated malaria in Vietnam, recrudescence rates were 16% after 7 days of quinine monotherapy (8). In studies conducted in Gabon, *Plasmodium falciparum in vitro* sensitivity to quinine was high and had not changed over the past decade (9). Although quinine monotherapy shows high efficacy in clinical trial settings, it has considerable disadvantages, mainly because of its poor tolerability and the prolonged treatment course. Poor adherence carries a high risk of treatment failure, particularly because quinine causes a syndrome of adverse effects known as cinchonism, including primarily tinnitus, nausea, and vertigo. Other reported side effects include high tone hearing impairment, dizziness, hypotension as well as headache and visual disturbances [14]. As a result of these side effects some studies have reported poor compliance to treatment, a randomized trial in Thailand recorded 71% adherence rate (11). Quinamax® is a formulation developed by Sanofi which comprises four alkoloids: (i)quinine (ii) quinidine, (iii) cinchonin and (iv) cinchonidine in this study we will use dry tablets (125mg), of whom the dosage is not adapted to children below 9 kg. The latter explains why children below 12 months will be excluded from our study.

Clindamycin is a lincosamide antibiotic derivative of lincomycin. It is very soluble in water. It inhibits the early stages of protein synthesis by a mechanism similar to that of the macrolides. It may be administered by mouth as capsules containing the hydrochloride or as oral liquid preparations containing the palmitate hydrochloride.
Clindamycin is used twice daily for 7 days at 10 mg/kg for children of 11 years and under. Clindamycin used in combination with quinine is safe but limited data is so far gathered.

3.2. Artemether –lumefantrine (AL)

This is a fixed-dose combination of artemether (a semi-synthetic artemisinin derivative) and lumefantrine (a slowly eliminated drug also referred to as benflumetol). The registered indications and branding for AL cover treatment of uncomplicated malaria caused by mono or mixed *Plasmodium* infections. The combination is expected to confer mutual protection against resistance and prevent recrudescence after artemether therapy. The components of this combination were originally studied and developed in China by the Academy of Military Medical Sciences (AMMS), Beijing and Kunming Pharmaceutical Factory (KPF), Kunming. The fixed combination has been registered in China since 1992 and has undergone further development when Novartis signed a collaborative agreement in 1994 with AMMS, KPF and CITITEC, the technology arm of the China International Trust and Investment Corporation (CITIC). Studies for the international registration started in 1995. AL of Novartis, marketed under the trademarks Riamet® and Coartem®, was registered in Switzerland in 1999, it is pre-qualified by the WHO and has since received marketing authorisation in several endemic and non-endemic countries. A recent review showed that the drug combination is highly efficacious against sensitive and multidrug resistant *falciparum* malaria as it offers the advantage of rapid clearance of parasites by artemether and the slower elimination of residual parasites by lumefantrine. (7)

3.3. Amodiaquine artesunate

ASAQ is safe, easy to use and efficacious and the second most used ACT worldwide (5;6). RDC, through the National Malaria Control Program has complied with the WHO recommendation by recommending since 2005 ASAQ as first line treatment for uncomplicated malaria. In a study conducted in 2004 in the eastern part of the country, the efficacy of ASAQ was estimated at 93% after PCR adjustment. (4). Twenty five trials (11,700 patients) carried out in Sub-Saharan Africa show a PCR-adjusted efficacy at day 28 of 94% (6). ASAQ is currently the second most used ACT globally (co formulated Co-arsucam® or ASAQ Winthrop®, Sanofi-Aventis(6). A study has been conducted in Burkina Faso in children under 5 and has shown that co-formulated ASAQ is well tolerated and its efficacy was 93% after PCR correction (5). We'll use this co-formulated ASAQ Winthrop® of Sanofi-Aventis, age dosed and put on the market since March 2007. This product has been pre-qualified by the WHO.

4. Study Design

This is a bi-centre, phase IIIb, randomized, open label, 3-arm trial. It will be performed in three steps and there will be informed consent for the first two steps:

4.1. Study phases

Pre-RCT phase

All patients will be treated with the first line treatment, ASAQ for RD Congo and AL for Uganda. Before treatment, a blood sample will be collected on filter paper (Whatman 3MM) for subsequent parasite genotyping. A plasma sample will also be collected and frozen for further immunological assessment (adjacent study). Patient will be followed up for the next 42 days. During this period, no systematic screening for malaria infection will be done. Instead, blood samples will be collected only for patients attending the health facilities with suspected clinical malaria. In case of confirmed failure before day 14, patients will be treated with quinine + clindamycin and excluded from the follow up as they would have reached one of the end points. Patients experiencing a clinical failure (fever and parasitaemia with any parasite density) between days 14 and 42 will be eligible for the second phase.

RCT phase

Clinical failures identified between day 14 and 42 (see above) will be randomised to either ASAQ, AL or quinine + clindamycin. The randomization list will be generated prior to the beginning of the study by the study statistician. Rescue treatment allocation will be concealed until the recruitment of the patient in the RCT phase (see section 4.7);

The interpretation of the PCR reading will be blinded/masked with regard to the treatment allocation of the patients (see section 4.5); An independent Data Safety Monitoring Board will review all efficacy and safety data (see section 14).

Assuming the true PCR corrected efficacy at 28 days of re-treatment with the same artemisinin-based combination therapy is 95%, a sample size of 248 patients (i.e., 124/site) is needed to show with 80% power that the efficacy is at least 90%. Recruiting the same number of patients on the alternative artemisinin-based combination therapy (i.e., AL in DRC and ASAQ in Ug) and half on quinine + clindamycin will allow the estimation

of the relative risk in efficacy between the treatment groups to within 5% assuming a similar efficacy of 95% of all 3 treatment groups. The total sample size for the RCT will be 620 (310 per site).

To allow for up to 15% drop-outs or unevaluable patients, we will recruit 714310 patients in each site (357). The 2:2:1 allocation ration is chosen to reduce the number of patients randomized to the more demanding quinine + clindamycin treatment regimen. In addition, this allows greater precision for the documentation of the efficacy and safety of ACT regimens in second line, rather than the standard recue treatment quinine + clindamycin. However, the inclusion of the quinine + clindamycin arm is necessary as a benchmark for assessing the efficacy of the ACT in second line and an internal consistency check of the study.

In DRC, based on the most recent study results (4,16) and the malaria endemicity at the study site the PCR uncorrected, we will need 1800 children to be enrolled for the initial phase of the study to obtain 310 patients recruited in the clinical trial.

In Uganda, following the same logic, we will need 1800 children to be enrolled for the initial phase of the study in RDC to obtain 310 patients recruited in the clinical trial

post-RCT phase

All patients included in the RCT phase and presenting a clinical or parasitological failure from day 14 onwards will be retreated with the country's first line treatment (AL or ASAQ). We expect about 50 patients per site to participate in this phase. All patients will be followed up exactly as during the RCT and study procedures will be also identical. In case of confirmed failure before day 14, patients will be treated with the first-line ACT and excluded from further follow up. The same procedures will be followed for each repetitive treatment failure occurring between 14 and 28 days after each treatment course (see figure) with a maximum of 3 post RCT follow up. The liver function and hematologic parameters will be monitored in participants exposed to 3 ASAQ courses or more.

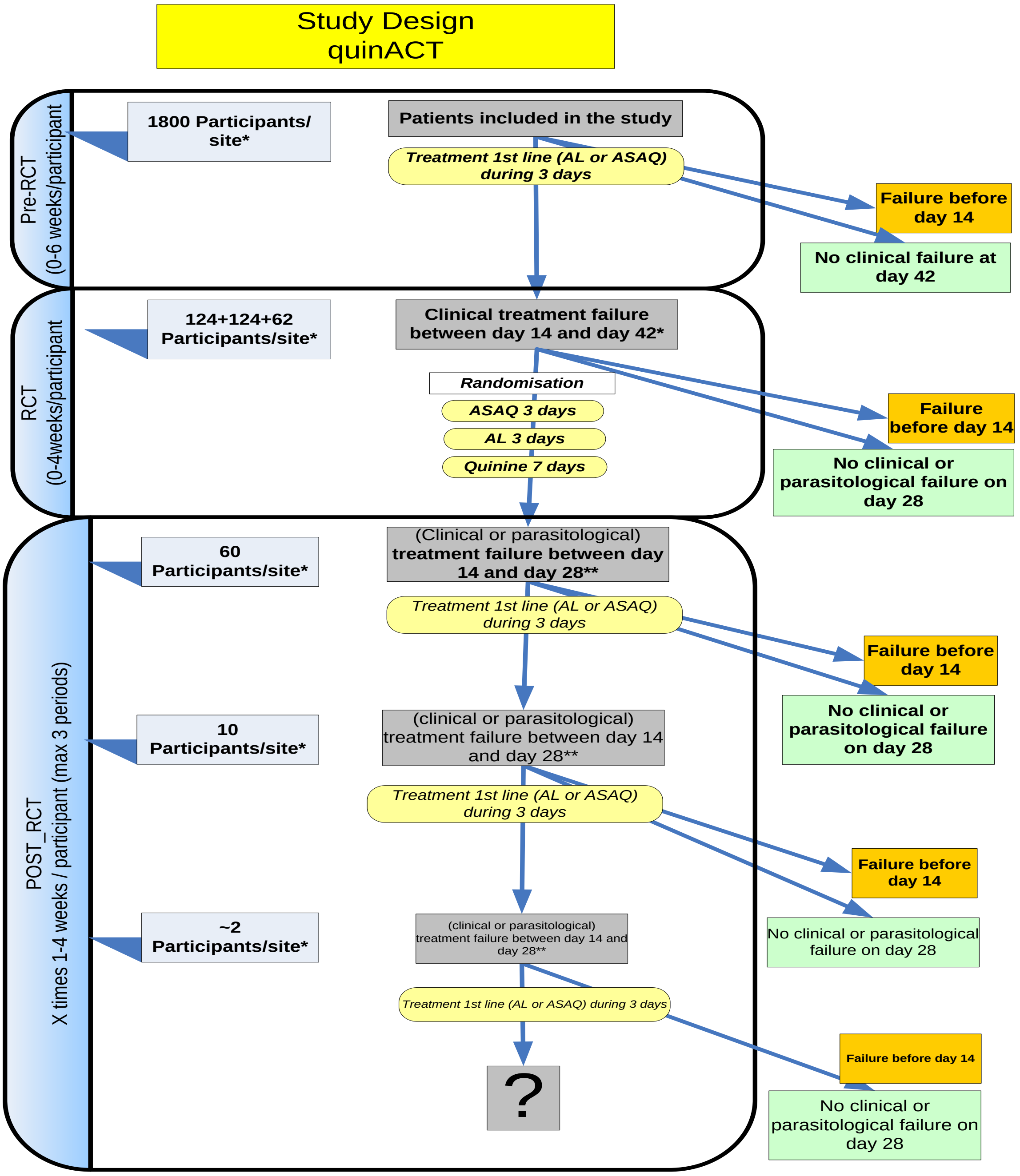

* treatment failures before day 14 will be treated with 7 days quinine (following national Guidelines)
** treatment failures before day 14 will be treated with a second ACT

Errata: Quinine 7 days should be replaced by Quinine + Clindamycine in flow chart above

4.2. Patient selection

Inclusion criteria Initial treatment (Pre RCT phase)

In order to be eligible, patients should satisfy the following inclusion criteria:

1. Males and Females aged between 12 months and 59 months inclusive. This criterion applies only for the recruitment in the first follow up. For the subsequent follow up, children having been included in the first follow up are eligible, regardless of their age.
2. Body weight of 9 Kg and above.
3. Microscopically confirmed, mono-infection of *Plasmodium falciparum* (parasitaemia ≥ 2,000/µL to 200,000/µL).
4. Fever (tympanic temperature at ≥ 38.0°C) or history of fever in the previous 24 hours.
5. Haemoglobin value ≥ 6.0 g/dl;
6. Signed (or thumb-printed and witnessed by an impartial witness, whenever parents/guardians are illiterate)

informed consent by the parents or guardians. Note the first informed consent will be asked at recruitment in the pre-RCT phase and it will cover the first 42-days follow up. The second informed consent will be asked at enrolment for the randomized trial and will cover the remaining period of the study.

7. Parents' or guardians' willingness and ability to comply with the study protocol for the duration of the study.

Exclusion criteria(Pre RCT phase)

Patients with at least one of the following criteria will be excluded:

1. Participation in any other investigational drug study (antimalarial or others) during the previous 30 days.
2. Known hypersensitivity and previous Serious Adverse Events related to the study drugs.
3. Severe malaria(WHO 2000) or danger signs: not able to drink or breast-feed, vomiting (> twice in 24hours), recent history of convulsions (>1 in 24h), unconscious state, unable to sit or stand.
4. Presence of intercurrent illness or any condition (cardiac, renal, hematologic, hepatic diseases) which would place the subject at undue risk or interfere with the results of the study, including known G6PD deficiency.
5. Patients who are taking drug which may prolong the QT (imidazole and triazole, antifungal agent).
6. Severe malnutrition (defined as weight for height <70% of the median NCHS/WHO reference).
7. Ongoing prophylaxis with drugs having antimalarial activity such as cotrimoxazole for the prevention of *Pneumocisti carini* pneumonia in children born to HIV+ women.

*WHO 2000: Severe falciparum malaria. *Trans. R. Soc. Trop. Med. Hyg.* **94**, 1-90.

For being eligible for the RCT the patient has to respond to the following criteria

Inclusion criteria Initial treatment (RCT phase)

1. Have been enrolled in the first phase
2. Recurrent *Plasmodium falciparum* infection with clinical symptoms.
3. Parents' or guardians' willingness and ability to comply with the study protocol for the duration of the study.
4. Signed (or thumb-printed whenever parents/guardians are illiterate) (second) informed consent by the parents or guardians. Note: the informed consent will cover the whole period of the study, including additional active follow ups

Exclusion criteria (RCT Phase)

Patients with any of the following criteria will not be admitted to the study:

1. Known hypersensitivity or serious drug-related adverse event to the study drugs.
2. Severe malaria.
3. Danger signs: not able to drink or breast-feed, vomiting (> twice in 24hours), recent history of convulsions (>1 in 24h), unconscious state, unable to sit or stand.
4. Treatment failure within 14 days in the first study phase.
5. Body weight below 9 Kg

4.3. Endpoints

4.3.1. Primary Endpoint

PCR adjusted efficacy at 28 days: the proportion of children with PCR adequate clinical and parasitological response at day 28 (ACPR28A): all early failures before day 7 plus the recurrent parasitaemias detected later and classified by genotyping as recrudescence.

The TF is defined according to the WHO criteria (WHO 2003) as the sum of early* and late** treatment failures.

*** Early Treatment Failure (ETF) (one of the following)**

(i) Development of danger signs or severe malaria (see Appendix V) on Day 0, Day 1, Day 2 or Day 3, in the presence of parasitaemia,
(ii) Parasite density on Day 2 > Day 0 count, irrespective of tympanic temperature,
(iii) Presence of parasitaemia on Day 3 with fever (tympanic temperature ≥ 38.0°C),
(iv) Parasitaemia on Day 3 ≥ 25 % of count on Day 0.

**** Late treatment failure (LTF)**

LTF is divided in late clinical and late parasitological failure.

Late Clinical Failure (LCF):
(i) Development of danger signs or severe malaria after Day 3 in the presence of parasitaemia, (See Appendix V for the criteria of severe malaria/danger signs).
(ii) Presence of parasitaemia and fever on any day from Day 4 to Day 28, without having previously meet the criteria of ETF.

Late Parasitological Failure (LPF):
Parasitaemia after day 3 in the absence of fever (tympanic temperature <38.0°C) and without having previously met the criteria of ETF or LCF

The **adequate clinical and parasitological response (ACPR)** is defined as absence of parasitaemia at the end of the follow up period (day 28), irrespective of axillary temperature without previously meeting any of the criteria of early and late treatment failure.

In the **PCR adjusted analyses**, patients with late asexual parasite reappearance (with or without fever) will be considered ACPR if the PCR analysis shows a new infection rather than a recrudescence.

4.3.2. Secondary Efficacy Endpoints

1. PCR unadjusted efficacy at 28 days: the proportion of children without (PCR not adjusted) treatment failure (TF28U): all treatment failures detected during the active follow up, regardless of genotyping.

2. 42 days clinical efficacy: all clinical treatment failures detected during the 42 days follow up for the first line treatment, with and without PCR adjustment. As no active monitoring of parasitaemia after day 3 is planned this includes ETF and LCF following WHO criteria.

3. Fever clearance time (FCT): Fever clearance time is defined as the time (in days) from the time of randomization to the first two consecutive measurements on 2 different days of tympanic temperature below 38.0°C.

4. Asexual parasite clearance time (PCT): Asexual parasite clearance time is defined as the time (in days) from time of randomization to 2 consecutive negative blood slides (collected at different days). The time to the event will be taken as the time to the first negative slide.

5. Gametocytaemia (prevalence and density) at day 7, 14, 21 and 28 after treatment.

6. Hb changes between day 0 and days 14 and 28.

Tertiary Efficacy Endpoints will be defined in the substudy protocols

4.3.3. Safety Endpoints

Subjects will be monitored throughout the study for possible development of adverse events. All adverse events will be recorded on the specific form in the CRF. Vital signs, and haematology will be monitored and changes in relevant laboratory parameters will be assessed

4.4. PCR analysis

Blood samples collected on filter paper for PCR genotyping will be analysed at the Institute of Tropical Medicine, Antwerp, Belgium. Sample will be collected

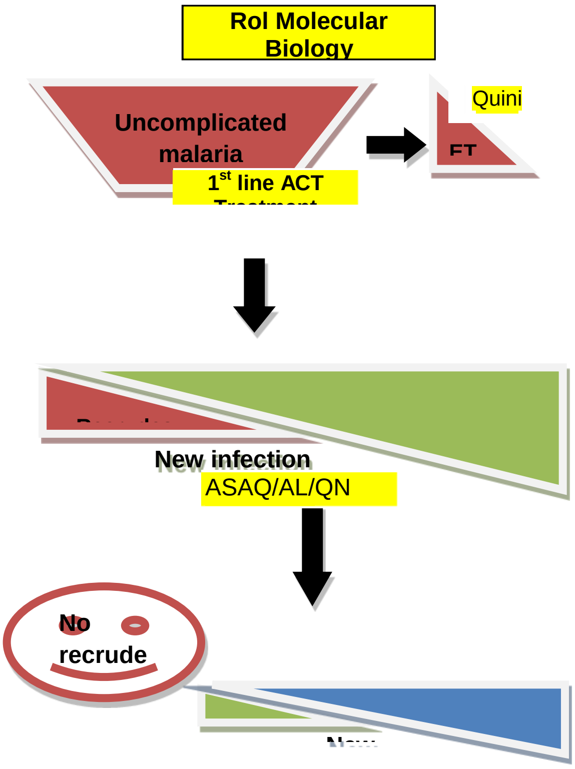

ETF =Early Treatment Failure
PCR plays a key role as determines if recrudescences will be eliminated after retreatment with 1st line ACT
PCR will determine firmly if
- ACTs can be used as retreatment or
- strong advocacy is needed to ban retreatment with ACTS within 6 weeks after initial treatment

according to the study-specific standard operating procedures.

4.4.1. Genotyping

Genotyping of the recurrent infections will be done by characterizing msp1, msp2 and glurp genes in the *Plasmodium falciparum* genome. PCR-amplification of DNA from a single parasite clone results in a single amplification product. For the three genes, each PCR-amplification product of a different size is considered to originate from a different clone of *Plasmodium falciparum* and reflects a different genotype. For the samples collected from the same patient at day 0 and day of recurrent parasitaemia, the length polymorphism of MSP1, MSP2 and GLuRP will be determined, i.e. the number of bands in each PCR reaction and their respective size. Results will be interpreted as follows:

4.4.2. Interpretation of PCR results

Recrudescence: For each marker (msp1, msp2 and glurp), at least one identical length polymorphism is found in the sample collected at day 0 and day of recurrent parasitaemia.
New infection: For at least one marker, length polymorphism is different between the sample collected at day 0 and that at day of recurrent parasitaemia.
Indeterminate: Samples that failed to produce a result due to an inability to amplify DNA at day 0 and/or day of recurrent parasitaemia.

4.5. Frozen Plasma samples

Frozen plasma samples will stored on site at -70C°at the sites They only will be transferred to a centralized if a research protocol adjacent to this study have been accepted by the concerned ECs. An agreement will be made between partners in this regards(will also be stipulated in agreement on publication policy). Only samples which have been allowed through the IC by the study participants will be transferred

4.6. Sample size

Assuming the true PCR corrected efficacy at 28 days of re-treatment with the same artemisinin-based combination therapy is 95%, a sample size of 248 patients (i.e., 124/site) is needed to show with 80% power that the efficacy is at least 90%.

Recruiting the same number of patients on the alternative artemisinin-based combination therapy (i.e., AL in DRC and ASAQ in Ug) and half as many on quinine + clindamycin will allow the estimation of the relative risk in efficacy between the treatment groups to within 5% assuming a similar efficacy of 95% of all 3 treatment groups.

To allow for up to 15% drop-outs or un-evaluable patients, we will recruit 357 patients in each site (124 on ASAQ and AL each, and 71 on quinine + clindamycin). The 2:2:1 allocation ration is chosen to reduce the number of patients randomized to the more demanding quinine + clindamycin treatment regimen. In addition, this allows greater precision for the documentation of the efficacy and safety of ACT regimens in second line, rather than the standard recue treatment quinine + clindamycin. However, the inclusion of the quinine + clindamycin arm is necessary as a benchmark for assessing the efficacy of the ACT in second line and an internal consistency check of the study.

In RDC, most recent study results (16) on the clinical efficacy of ASAQ in the Equator Province in 2004 show a PCR uncorrected clinical failure rate of 41% at day 28. However in Kinshasa, due to a lower reinfection incidence the PCR uncorrected clinical failure rate can be expected to be lower at approximately 25%. Assuming a clinical failure rate of 25% and assuming that 80% of failing patients would be eligible for the RCT and their guardians provide informed consent, we will need 1800 children to be enrolled for the initial phase of the study in RDC to obtain 310 patients recruited in the clinical trial.

In Uganda, most recent study results on the clinical efficacy of AL in Mbarara in 2005, show a PCR unadjusted clinical failure rate of 23% after 28 days of follow up. Empirically, expecting a clinical treatment failure rate of 25%, assuming a lost to follow up of 10% and assuming that 80% of the falling patients would be eligible for the RCT after their guardians have provided informed consent, we will need 1800 children to be enrolled for the initial phase of the study in Uganda to obtain 310 patients recruited in the clinical trial (13).

The recruitment will continue in each country till the required sample size of 310 children recruited in each country in the RCT is reached. If recruitment rates in the RCT are lower than expected, the study may be extended to additional research sites.

4.7. Study Procedures and Duration of patient follow up

The critical steps for the study period are described in Appendix VII.

4.7.1. Pre-RCT phase: First line treatment

Each patient will be followed up actively after recruitment in the pre-RCT phase for 42 days without systematic screening.

The community health worker (CHW) is in charge to contact the parents or legal guardian before each scheduled visit. If difficulties arise, the CHW will make home visits. From day 14 onwards, a range of ±1 day will be accepted. Patients will be assessed as summarized in the following flow-chart.

The following steps will be taken:

Follow-up Chart Pre-RCT Phase

Day	0	1	2		14[1]		28[1]		42[1]	Any other day[1]
History (symptoms)	X				X		X		X	X
Examination (clinical)	X	X	X		X*		X*		X*	X
Temperature	X	X	X		X*		X*		X*	X
Rapid test	X									
Blood film	X		X*		X*		X*		X*	X
Filter paper PCR	X		X*		X*		X*		X*	X
Informed consent	X									
Haematology	X									
Plasma sample	X									
Treatment	X	X	X							
Adverse events	X	X	X		X		*X*		*X*	*X*
Concomitant medications	X	X	X		X		*X*		*X*	*X*

X = perform this task; * only if symptoms indicate a possible clinical treatment failure

[1] For a clinical treatment failure see day 0 RCT phase

A Case Report Form consisting of demographic and physical/clinical information will be completed;

<u>Day 0: screening visit/ administration of the First line medication</u>

1 <u>History, Physical and Clinical Examination</u>

An anamnesis and general physical examination will be performed (see Appendix I).

A clinical examination will be performed (see Appendix I): symptoms and tympanic temperature.

2 <u>Rapid test</u>

A Rapid test will be used as pre- screening to verify the presence of *P. falciparum*. If the test is positive a thick and thin blood smear will be obtained. If the test is negative, the patient will be refered to the health facility management system.

3 <u>Blood Slide</u>

A thick and thin blood smear will be obtained from the subject to verify the presence of *P. falciparum* and to calculate the parasite density. Thick and thin blood films will be prepared, dried and stained with Giemsa stain according to standard operating procedures.

Parasite density will be calculated by counting the number of asexual parasites per 200 leukocytes in the thick blood film, based on an assumed WBC of 8,000 /µl by light microscopy at 1000 X magnification. One hundred high-powered fields (HPF) will be examined (independent of presence or absence of asexual parasite stages). The parasite density per microlitre will be calculated using the following formula:

$$\text{Parasite density} / \mu l = \frac{\text{Number of parasites counted} \times 8{,}000}{\text{Number of leukocytes counted}}$$

A few drops of bloods will be put impregnated filter papers (Whatmann 3MM) for molecular analysis,

4 1st Informed consent

After an in-depth interview, with one physician of the study or with another qualified person, the parent or legal guardian) will be asked to document her consent by signing an informed consent. The signed informed consent (or thumb-printed whenever the parents/guardians are illiterate) must be obtained before any tests or evaluations related to the study are carried out. However, a rapid diagnostic test as used as screening and confirmed by thick blood film before the informed consent can be done as this can be considered a normal procedure for the management of patients suspected having clinical malaria. The preparation of the PCR samples, with a few drops of blood will be done at the same moment.

5 Weight

Weight will be measured and BMI calculated.

6 Laboratory Tests

Venous blood (3ml) will be used to measure hemoglobin (HemoControl®) and other tests that the clinician may request for. Residual plasma will be separated, stored and transported at –70°C to be assayed in adjacent studies i.e. malarial antibody quantification by ELISA and FACS (described in independent protocol)

7 Supervised treatment.

See 4.8 treatment administration for modalities

8 Adverse Events Report

All adverse events occurred after the drug's administration will be recorded. See section 7.1.4 for the details on the information collected.

9 Concomitant Medications

Any medications taken by the study subject after the drug's administration will be recorded in the CRF.

Day 1 and 2: *Open Label Treatment Period*

1 Physical and Clinical Examination

A general physical examination and a clinical examination will be performed: symptoms, axillary temperature (electronic thermometer).

2 Concomitant Pharmacological Treatments

Concomitant medications taken by the patients will be recorded. For a list of allowed and disallowed medications, see section 5.

3 Adverse Events Report

All adverse events will be recorded. See section 8.1.4 for the details on the information collected

4 Administration of the Study Drugs

See 4.8 treatment administration for modalities

Day 7, 14, 28, 42 and Unscheduled visits throughout follow up

The following information will be collected:

1 Medical History

Symptoms and other relevant episodes between visits will be recorded.

2 Adverse Events Report

All adverse events will be recorded. See section 8.1.4 for the details on the information collected.

3 Concomitant Medications

Any medications taken by the study subject will be recorded in the CRF

On day 42, a thick and thin blood smear will be obtained and filter paper impregnated before patients without clinical failure leave the study.

If a clinical failure is observed see day 0: rescue treatment.

4.7.2. RCT-phase

Randomization and treatment allocation

Patients, after record of the second informed consent, will be randomly assigned to ASAQ, AL or quinine + clindamycin. The randomization list will have been computer generated before the start of the study by the study statistician. At the study site, treatment allocation and administration of medications will be performed by the study physician or nurse.

Flow chart (Post-) RCT Phase

Day	0	1	2	3	4-6	7	14[1]	21[1]	28[1]	Any other day[1]
History (symptoms)	X					X	X	X	X	X
Examination (clinical)	X	X	X	X		X	X	X	X	X
Temperature	X	X	X	X		X	X	X	X	X
Blood film	X		X	X		X	X	X	X	X
Filter paper PCR	X		X	X		X	X	X	X	X
Informed consent	X									
Haematology	X						X		X	X
Plasma sample	X									
Treatment	X	X	X	X[1]	X[1]					
Adverse events	X	X	X	X	X[1]	X	X	X	*X*	*X*
Concomitant medications	X	X	X	X	X[1]	X	X	X	*X*	*X*

X[1] = quinine + clindamycin treatment administration; only for RCT phase

[1] For a treatment failure see day 0 Post-RCT phase

All study medications for patients randomised to ACT's shall be administered under direct observation. Study medications for patients randomised to quinine + clindamycin shall be administered under direct observation on the first 3 days (9 doses). For the remaining quinine + clindamycin doses, the two doses for the day shall be administered in the clinic under direct supervision by the study nurse and the third dose for the day shall be administered by the parents or guardians at home. The site will take site adapted measures to warrant full treatment adherence. Patients will have the option to be admitted for observation and study drug administration or to commute from home. Parents/guardians will be encouraged to return to the clinic for follow up assessments on days 3, 7, 14, 21, 28 and on any unscheduled day if the child is not well.

Day 0: recruitment in the RCT-phase/ randomization to rescue treatment

The day of treatment failure on the former 42-days period following the treatment of the first malaria episode, corresponds to the day 0 of the RCT-phase.

1 History, Physical and Clinical Examination

A general physical examination will be performed (see Appendix I).

A clinical examination will be performed (see Appendix I): symptoms and tympanic temperature (electronic thermometer).

2 Blood Slide

A thick and thin blood smear will be obtained from the subject to verify the presence of *P. falciparum* and to calculate the parasite density. Thick and thin blood films will be prepared, dried and stained with Giemsa stain according to standard operating procedures.

Parasite density will be calculated by counting the number of asexual parasites per 200 leukocytes in the thick blood film, based on an assumed WBC of 8,000 /µl by light microscopy at 1000 X magnification. One hundred high-powered fields (HPF) will be examined (independent of presence or absence of asexual parasite stages). The parasite density per microlitre will be calculated using the following formula:

$$\text{Parasite density} / \mu l = \frac{\text{Number of parasites counted} \times 8{,}000}{\text{Number of leukocytes counted}}$$

A few drops of bloods will be put impregnated filter papers (Whatmann 3MM) for molecular analysis,

3 2nd Informed consent

After an in-depth interview, with one physician of the study or with another qualified person, the parent or legal guardian) will be asked to document her consent by signing an informed consent. The signed informed consent (or thumb-printed whenever the parents/guardians are illiterate) must be obtained before any tests or evaluations related to the study are carried out. However, a thick blood film before the informed consent can be done as this can be considered a normal procedure for the management of patients suspected having clinical malaria. The preparation of the PCR samples, with a few drops of blood will be done at the same moment

4 Weight

Weight will be measured and BMI calculated.

5 Laboratory Tests

Venous blood (3ml) will be used to measure hemoglobin (HemoControl®) and other tests that the clinician may request for. Residual plasma will be separated, stored and transported at –70°C to be assayed in adjacent studiesi.e. malarial antibody quantification by ELISA and FACS (described in independent protocol).

6 DOT Administration of the study drugs

See 4.8 treatment administration for modalities

7 Adverse Events Report

All adverse events will be recorded. See section 7.1.4 for the details on the information collected.

8 Concomitant Medications

Any medications taken by the study subject will be recorded in the CRF.

Day 1 and 2: *Open Label Treatment Period*

1 Physical and Clinical Examination

A general physical examination and a clinical examination will be performed: symptoms, tympanic temperature (electronic thermometer).

2 Blood Slide

A thick blood smear will be obtained on Day 2 to check for presence and density of asexual and sexual stages of *P*. *falciparum*.

3 Concomitant Pharmacological Treatments

Concomitant medications taken by the patients will be recorded. For a list of allowed and disallowed medications, see section 5.

4 Adverse Events Report

All adverse events will be recorded. See section 8.1.4 for the details on the information collected

5 DOT Administration of the Study Drugs

See 4.8 treatment administration for modalities

Day 3: *follow-up and Quinine + clindamycin group open label Treatment Period*

1 Physical and Clinical Examination

A general physical examination and a clinical examination will be performed: symptoms, tympanic temperature).

2 Blood Slide

A thick blood smear will be obtained to determine the presence and the density of asexual and sexual stages of *P*. *falciparum.*

3 Concomitant Pharmacological Treatments

Concomitant medications taken by the patients will be recorded. For a list of allowed and disallowed medications, see section 4.

4 Adverse Events Report

All adverse events will be recorded. See section 8.1.4 for the details on the information collected

5 Administration of study drugs for quinine + clindamycin group

Day 4-6: Quinine + clindamycin group open label Treatment Period

1 Physical and Clinical Examination

A general physical examination and a clinical examination will be performed: symptoms, tympanic temperature physical and clinical examination

2 Administration of the Study Drugs for quinine + clindamycin

3 Concomitant Pharmacological Treatments

Concomitant medications taken by the patients will be recorded. For a list of allowed and disallowed medications, see section 4.

4 Adverse Events Report

All adverse events will be recorded. See section 8.1.4 for the details on the information collected

5

Day 7, 14 and 21: *follow-up*

As at Day 3. In addition, the following information will be collected:

1 Medical History Physical and Clinical Examination

A general physical examination and a clinical examination will be performed: symptoms, tympanic temperature (electronic thermometer). Symptoms and other relevant episodes between visits will be recorded.

2 Laboratory Tests

Venous blood (3 ml) will be used to prepare blood films, impregnated filter papers (Whatmann 3MM) for molecular analysis, measurement of hemoglobin (HemoControl®) on day 14 and other tests that the clinician may request for.
If a clinical or parasitological failure is observed see day 0: further rescue treatment

3 PCR

A blood sample will be collected on filter paper (Whatmann 3MM) for later genotyping.

Day 28: *follow-up*

As Day 7.

1 PCR

A blood sample will be collected on filter paper (Whatmann 3MM) for later genotyping.

4 Laboratory Tests

Blood haemoglobin, and other tests that the clinician may request for.

Unscheduled visits throughout the RCT-phase follow up

During this visits, the same procedures as day 7 will be applied. Haemoglobin will be measured if the patient is classified as treatment failure.

The PCR readings will be centralised and masked to the treatment allocation of study subjects. It will be done by personnel different from the treating physician/investigator. In addition, a centralized and independent double-check of at least 10% of blood slides and filter paper blood samples (PCR) will be carried-out. The percentages of slides and films to be reviewed and the corresponding statistical justifications will be specified in the Statistical Analysis Plan.

If a clinical or parasitological failure is observed see day 0: further rescue treatment

4.7.3. Post RCT-Phase- Further rescue treatment

For failures happening beyond day 14, procedures and flow chart are identical to 4.7.2. rescue treatment but patients will receive the recommended first line treatment. The liver function and hematologic parameters will be monitored in participants exposed to 3 ASAQ courses or more.

4.8. Randomisation and treatment allocation

A randomisation list of blocks of varying size and stratified according to the number of recruitment points in each site will be provided. Sealed envelopes labelled with the patients unique code and containing the treatment allocated to the patient will also be provided according to the above mentioned list. This will guarantee concealment as the envelope will be opened only after recruitment

4.9. Treatment administration

The study treatment will be administered under the direct supervision of a study nurse. The patient will have the option of being admitted for the first 3 days of treatment administration or going home and returning to the clinic for the remaining doses. For patients who choose to go home between doses, a home visitor will collect information on where they live. If a patient fails to return to the clinic in a timely manner for their daily dose of study drug, they will be visited at home and brought to the clinic the same day. If patients miss any dose of study drugs, they will not be excluded from the study. The study nurse will record the date and time study drugs are administered. From day 3 to 6 the remaining quinine + clindamycin doses shall be administered. The two doses for the day shall be administered in the clinic under direct supervision and the third dose for the day shall be administered by the parents/guardians at home. Study drugs given to young children as specified by the manufacturers of each product.(Appendix II). After drug administration, patients will be kept for sixty minutes in the clinic. A dose will be repeated in full if vomiting occurs within 30 minutes of administration , and in half if vomiting occurs between 30 and 60 minutes. This event will be documented in the case record form (CRF). If vomiting persists beyond two additional doses, the patient will be withdrawn from the study and treatment changed.

Upon receipt of the study treatment supplies, an inventory will be performed and a drug receipt log filled out and signed by the pharmacy staff accepting the delivery. Accountability for the drug at all study sites is the responsibility of the principal investigator. The investigator will ensure that the drug is used only in accordance with this protocol. Accountability records will include dates, quantities, lot numbers, expiration dates, and patient numbers.

5. CONCOMITANT THERAPIES

5.1. Disallowed Concomitant Drug Therapies during the active follow ups

Any antimalarial, or antibiotic with antimalarial activity (erythromycin or other macrolides), co-trimoxazole or other sulfonamides, any tetracycline including doxycycline, and quinolones, clindamycin.

5.2 *Allowed Concomitant Drug Therapies*

During the trial, patients can be prescribed drugs e.g. paracetamol, and antibiotics with no known antimalarial activity (penicillins, cephalosporins). The dose, route, time and duration of any concomitant medical treatment will recorded in the CRF.

5.3 *Special Conditions*

Parents or guardians will be discouraged from obtaining drugs from any other source such as private pharmacies, markets or clinics. Parents/guardians will be encouraged to bring their children to the study clinic if their child is unwell or if they are worried about their child's health.

5.4 *Rescue Treatments*

All patients who develop severe/complicated malaria during active follow-up will be treated following National Guidelines. Patients randomized to ACT and experiencing treatment failure before 14 days will be treated with quinine 10 mg/kg orally three times a day in combination with clindamycin 10 mg/kg twice daily for 7 days. Patients who fail to quinine + clindamycin therapy shall be treated with the first line therapy (ASAQ or AL).

6. PATIENT WITHDRAWAL CRITERIA

Patients will be excluded from further assessment if there is withdrawal of informed consent.
Serious adverse events related to the study drug are also a reason for withdrawal from the study.
Intake of drugs stated in 5.1. leads to withdrawal of the patient from active follow up.
Participants also may be withdrawn if the study sponsor, government or regulatory authorities, or site Ethics Committee terminate the study prior to its planned end date.
Every reasonable effort will be made to complete a final evaluation of participants who terminate from the study prior to the planned termination time period and study staff will record the reason(s) for all withdrawals from the study in participants' study records.
At the time of withdrawal, all study procedures outlined for the End of Study visit should be completed. The primary reason for a patient's withdrawal from the study is to be recorded in the source documents.
Any withdrawal or lost to follow-up shall be documented and notified to the site principal investigator as soon as possible.

7. PROTOCOL VIOLATIONS

A protocol violation occurs when an event happens that does not allow for accurate interpretation of response to treatment. Protocol violations will be defined in the statistical analysis plan.

8. SAFETY VARIABLES

Safety and tolerability of the treatments will be evaluated by recording Adverse Events (AEs) and grading, laboratory, and vital signs evaluations.

8.1. Adverse Events

At each visit, the Investigator will ascertain the occurrence of any adverse events since the last visit. Any event must be recorded on the CRF.

8.1.1. Definition of an adverse event

An AE is any untoward medical occurrence in a patient or clinical investigation subject administered a pharmaceutical product and which does not necessarily have a causal relationship with this treatment.
An AE can therefore be any unfavourable and unintended sign (that could include a clinically significant abnormal laboratory finding), symptom or disease temporally associated with the use of a medicinal product, whether or not considered related to the medicinal product.

Examples of an AE **include**:

1. Significant or unexpected worsening or exacerbation of the condition under study.
2. Exacerbation of a chronic or intermittent pre-existing condition including either an increase in frequency and/or intensity of the condition.
3. New conditions detected or diagnosed after investigational product administration even though it may have been present prior to the start of the study.
4. Signs, symptoms, or the clinical sequelae of a suspected interaction.
5. Signs, symptoms, or the clinical sequelae of a suspected overdose of either investigational product or a concurrent medication (overdose as such should not be reported as an AE/SAE).
6. Significant failure of expected pharmacological or biological action.

Examples of an AE **do NOT include** a/an:

1. Situations where an untoward medical occurrence did not occur (social and/or convenience admission to a hospital).
2. Anticipated day-to-day fluctuations of pre-existing chronic disease(s) or condition(s) present or detected at the start of the study that do not worsen.

8.1.2. Severity, relationship of event to study drug, and outcome

The severity of a clinical adverse event is to be scored according to the following scale:

1	Mild	Awareness of sign or symptom, but easily tolerated
2	Moderate	Discomfort enough to cause interference with usual activity
3	Severe	Incapacitating with inability to work or perform usual activity
4	Life-threatening	Patients at risk of death at the time of the event

Assessment of Causality

The investigator is obligated to assess the relationship between investigational product and the occurrence of each AE/SAE. The investigator will use clinical judgment to determine the relationship. Alternative causes, such as natural history of the underlying diseases, concomitant therapy, other risk factors, and the temporal relationship of the event to the investigational product will be considered and investigated. The investigator will also consult the drug information and the DSMB as needed in the determination of his/her assessment.

There may be situations when an SAE has occurred and the investigator has minimal information to include in the initial report to TMG. However, it is very important that the investigator always make an assessment of causality for every event prior to transmission of the SAE report to TMG. The investigator may change his/her opinion of causality in light of follow-up information, amending the SAE case report form accordingly.

The relationship of an adverse event to study drug is to be assessed according to the following definitions:

1 Definitely unrelated

Should be reserved for those events which occur prior to test drug administration (e.g., washout or single-blind placebo) or for those events which cannot be even remotely related to study participation (e.g. injury caused by a third party).

2 Unlikely

There is no reasonable temporal association between the study drug and the suspected event and the event could have been produced by the subject's clinical state or other modes of therapy administered to the subject.

3 Possible

The suspected adverse event may or may not follow a reasonable temporal sequence from study drug administration but seems to be the type of reaction that cannot be dismissed as unlikely. The event could have been produced or mimicked by the subject's clinical state or by other modes of therapy concomitantly administered to the subject.

4 Probable

The suspected adverse event follows a reasonable temporal sequence from study drug administration, abates upon discontinuation of the drug, and cannot be reasonably explained by the known characteristics of the subject's clinical state.

5 Definitely related

Should be reserved for those events which have no uncertainty in their relationship to test drug administration: this means that a rechallenge was positive.

The outcome of each AE must be assessed according to the following classification:

- completely recovered :	The patient has fully recovered with no observable residual effects
- not yet completely recovered :	Improvement in the patient's condition has occurred, but the patient still has some residual effects
- deterioration :	The patient's overall condition has worsened
- permanent damage :	The AE has resulted in a permanent impairment
- death :	The patient died due to the AE
- ongoing :	The AE has not resolved and remains the same as at onset
- unknown :	The outcome of the AE is not known because the patient did not return for follow-up (lost to follow-up)

8.1.3. Definition of a serious adverse event

A serious adverse event (experience) (SAE) or reaction is any untoward medical occurrence that at any dose fulfils at least one of the following criteria:

* results in death;

* is life-threatening;

  NOTE: The term 'life-threatening' in the definition of 'serious' refers to an event in which the subject was at risk of death at the time of the event. It does not refer to an event, which hypothetically might have caused death, if it were more severe

* requires hospitalization (other than for drug administration) or prolongation of existing hospitalization;

  NOTE: In general, hospitalization signifies that the subject has been detained (usually involving at least an overnight stay) at the hospital or emergency ward for observation and/or treatment that would not have been appropriate in the physician's office or out-patient setting. Complications that occur during hospitalization are AEs. If a complication prolongs hospitalization or fulfills any other serious criteria, the event is an SAE. When in doubt as to whether "hospitalization" occurred or was necessary, the event should be considered an SAE. Hospitalization for elective treatment of a pre-existing condition that did not worsen from baseline is not considered an AE, nor hospitalization for non-medical reasons (e.g., the patient stays at the hospital overnight because she leaves too far and/or there is not transport).

* results in persistent or significant disability/incapacity;

  NOTE: The term disability means a substantial disruption of a person's ability to conduct normal life functions. This definition is not intended to include experiences of relatively minor medical significance such as uncomplicated headache, nausea, vomiting, diarrhea, influenza, and accidental trauma (e.g. sprained ankle) which may interfere or prevent everyday life functions but do not constitute a substantial disruption.

* Medical or scientific judgment should be exercised in deciding whether reporting is appropriate in other situations, such as important medical events that may not be immediately life-threatening or result in death or hospitalization but may jeopardize the subject or may require medical or surgical intervention to prevent one of the other outcomes listed in the above definition. These should also be considered serious. Examples of such events are invasive or malignant cancers, intensive treatment in an emergency room or at home for allergic bronchospasm, blood dyscrasias or convulsions that do not result in hospitalization, or development of drug dependency or drug abuse.

All serious adverse events, whether or not deemed drug-related, or expected, must be reported immediately or within 24 hours (one working day), using the Serious Adverse Event Notification Form, by telefax or email to +32-2652875 or international.health@ua.ac.be (International Health Unit, Department of Epidemiology and Social Medicine, University of Antwerp, Universiteitsplein 1, BE-2610 Antwerp (Wilrijk)**;**
Fax should state "Urgent Serious Adverse Event" and study code on cover page.
All other AEs not fulfilling the criteria of immediate reporting must be recorded on the Case Report Form. This AE information will be collected on a regular basis during the clinical trial.

8.1.4. Reporting of adverse events

For all adverse events identified, an adverse event report form will be completed.

For each possible adverse event identified and considered as **serious**, a serious adverse event notification form will be completed.

The following information will be recorded for all adverse events:

1) Study randomization number
2) Description of event
3) Date of event onset
4) Date event reported
5) Severity of the event
6) Possible relationship of the event to study medication
7) Is the event serious?
8) Initials of the person reporting the event
9) Was the event episodic or intermittent in nature?
10) Outcome of adverse event
11) Action taken
12) Date event resolved.

A severity grading scale, based on toxicity grading scales developed by the WHO and the National Institutes of Health, Division of Microbiology and Infectious Diseases, will be used to grade severity of all symptoms, physical exam findings, and haemoglobin results (see Appendix I). Any new event, or an event present at baseline that is increasing in severity, will be considered as an adverse event.

8.1.5. Length of follow-up for adverse events

AEs presenting during the study: A patient still experiencing an AE at the end of a follow up, i.e. at day 42 or 28 will be managed as follows:

- If the AE has been detected and reported before the last visit and
  - it is mild (Grade 1), the patient will be managed according to good medical practice and the active follow up will be stopped. The end date for the AE will be recorded as Day 28.
  - If its grade is more than 1, the patient will be followed until the AE resolves, improves, or stabilizes.
- If the AE is new, the AE will be reported and the patient will be followed until the AE resolves, improves, or stabilizes.

For patients classified as clinical treatment failures (ETF/LCF/LPF):
Formal study follow-up ends when a patient is classified as a treatment failure (ETF or LTF/LPF), and patients should be treated and managed according to good medical practice. Additional follow-up for AEs in patients classified as treatment failures, is not typically indicated, unless the AE is serious, or is felt to be probably or definitely related to the study medications..

For patients with Serious Adverse Events (SAEs):

Any patient who experiences a Serious Adverse Event should be followed until the SAE resolves or improves (< grade 1). Although formal study follow-up is typically terminated when a patient is classified as a treatment failure, any patient with severe malaria / danger signs should be followed up to ensure their SAE has resolved / improved.

8.2. Laboratory Evaluations

Blood samples will be properly labelled with patients' initials, study number, the study day and the date the sample is taken. Haematology assessments will be performed locally at sites.
All laboratory results will be reported in Standard International Units or in conventional units.
Blood samples collected on filter paper for PCR genotyping will be analysed at the Institute of Tropical Medicine, Antwerp, Belgium. Sample will be collected according to standard operating procedures.

9. CASE REPORT FORM (CRF)

All data and observations must be initially documented in the Case Report Form or copied from source documents to the CRF (i.e. laboratory data)

For this study we'll use DATAfax©

10. MONITORING AND QUALITY ASSURANCE

The Sponsor will share this task with the The Amsterdam Institute for Global Health and Development (AIGHD) based in Uganda. An agreementwill be signed in this regard. The task of the Monitor is to verify the best conduct of the study through frequent contacts by phone and in person with the Principal Investigator and site staff, in accordance with the Standard Operating Procedures and Good ClinicalPractice, with the purposes of facilitating the work and attaining the objectives of the study. These visits will enable the Monitor to maintain current, personal knowledge of the study through review of the records, comparison with source documents, observation and discussion of the conduct of the study with the Investigator. Each site should be visited 5 times during the conduct of the trial, including a pre-study visit and a close-out visit. The monitor will carry out at least 30% source data verification on the First Phase of the study (pre-RCT phase) and 30% source data verification on the second Phase of the study (RCT and post RCT phase); however, these percentages will be increased in case of serious or systematic findings during the SDV.

The investigator shall maintain source documents for each patient in the study, consisting in case and visit notes (hospital or clinic medical records) containing demographic and medical information, laboratory data, and the results of any other tests or assessments The data on the CRF shall be either the source or traceable to the source documents in the patient's file. The investigator shall keep one copy of the original informed consent form signed by the parent/guardian (the other will remain with the parent/guardian). The investigator shall give the monitor, auditors, inspectors, IRB, EC full access to all relevant source documents to confirm their consistency with the CRF entries.

11. DATA MANAGEMENT

IDI Uganda will be in charge of centralized data management. Clinical data, as explained above, will be collected and recorded on appropriate paper Case Report Forms (CRF's). Laboratory data, as requested in the protocol will be registered at the laboratory and recorded onto the CRF. All data will then be processed from the CRF's into an electronic database. During the conduct of the study, data will be verified and reviewed to produce and maintain high quality data. All unresolved issues will be queried and resolved before locking the database. Data transfer and handling is done with appropriate security measures and with regard to rights, safety and well-being of trial subjects.

A report on data management process will be produced. The report will include.

- A full field listing and description of the file structure of the electronic data
- Reference ranges and units for laboratory data
- A list a brief description of all programs run on the data
- Level of errors found at each stage of checking the data
- General comments on data quality and significant problems encountered with the data
- A detailed list of any unresolved data queries
- A statement of any queries/errors which have not been corrected on the database
- A statement of the storage location of the electronic database

The central data base will be managed at IDI in Kampala, Uganda by a data manager who will run regular consistency checks and produce queries to be resolved by the local investigators. The statistician (based at CTU ITM) will review the database prior to finalisation and report on any problem encountered during the analysis.

12. Statistical analysis

A detailed analysis plan will be drawn up prior to the analysis. The statistical analysis of the study will be performed by the RDC PI Dr Hypolite Muhindo Mavoko in consultation with statisticians at the ITM and/or UA who will also review the analysis plans and analysis results.

1) Baseline comparability

Children in the treatment groups in each country will be described separately with respect to baseline characteristics. The clinical importance of any imbalance will be noted though statistical tests of significance will not be undertaken.

2) Efficacy Analyses

Primary

The primary hypothesis of the study is that the PCR adjusted efficacy at 28 days of re-treatment with the same artemisinin-based combination therapy is at least 90%. This hypothesis will be tested by constructing a two-sided 95% confidence interval (CI) for the proportion of children without PCR adjusted rescue treatment failure. If the 95% CI lies entirely above 90% the hypothesis is accepted.

The confidence interval will be constructed using Wilson's score method, pooling the data from the two countries (i.e., ASAQ treatment group in DRC and AL treatment group in UG).

In addition, the relative efficacy of the re-treatment with the same artemisinin-based combination therapy compared to treatment with quinine + clindamycin and treatment with another artemisinin-based combination therapy will be assessed using a log-binomial models (14) with fixed effects for treatment group and country. From this model, the relative risks of treatment success will be estimated together with their 95% CI. Appropriateness of pooling the results over the two countries will be assessed by testing country by treatment interactions in this log-binomial model.

For the efficacy analysis, both an intention-to-treat and a per-protocol approach will be adopted, with the intention-to-treat analysis being the primary approach. Rules for inclusion/exclusion of children from the per-protocol population will be specified in advance.

Secondary analyses

Statistical methods for secondary analyses will be described in the data analysis plan.

Every effort will be made to minimize the amount of missing data in the trial. Whenever possible, information on the reason for missing data will be obtained. Sensitivity analyses will be undertaken to assess the robustness of the conclusions to the missing data.

3) Safety Analyses

All non-serious and serious adverse events will be grouped according to a pre-specified side-effect coding system and tabulated for each treatment group. In contrast to the primary efficacy analysis, actual treatment groups will be assessed

The number (and percentage) of patients experiencing any adverse event, any serious adverse event, and any drug-related serious adverse will be compared between treatment groups using Fisher's exact test. Safety will be analyzed using the all-patients-treated approach.
Data analysis will be primarily performed by the study statistician using STATA statistical software packages. Descriptive statistics will be used to summarize baseline characteristics of study patients. Efficacy and safety data will be evaluated using a modified intention-to-treat analysis and will only include patients who meet all selection criteria. Categorical variables will be compared between the treatment groups using odds ratio, chi-square tests or Fisher's exact tests and continuous variables will be compared using t-tests or non-parametric tests. A p-value of < 0.05 will be considered statistically significant. Estimates of the risk of failure for all primary outcomes will be made using the Kaplan-Meier product limit formula. Patients excluded after enrollment will be censored at the time of their last assessment. Additionally, for genotyping adjusted outcomes, patients with recurrent malaria or recurrent parasitemia due to new infections will be censored. Stratified analysis shall be done for outcomes in patients with recurrent symptomatic (ETF, LCF) and recurrent asymptomatic (LPF) malaria. The number (and percentage) of patients experiencing each adverse event will be compared between the treatment groups. No formal statistical testing will be undertaken.

13. INVESTIGATOR RESPONSIBILITY

The term "Investigator" as used in this protocol and on the CRFs refers to the Principal Investigator or a member of the staff that the Investigator designates to perform a certain duty under this protocol. The Investigator is ultimately responsible for the conduct of all aspects of the study. For all other relevant Investigator responsibilities see "CPMP/ICH/135/95 Topic E6 - Guideline for Good Clinical Practice", Chapter 4.

14. ADMINISTRATIVE PROCEDURES

14.1. Regulatory Authorities and Ethical Review Committee

This protocol will be submitted for approval by the Ethical committee of University of Antwerp, EC Amsterdam Medical Centre, Netherlands and the national committees of RDC and Uganda: EC of Public Health School in Kinshasa and the Makerere University Review , Ethics Committee and the Uganda National Council of Science and Technology, Patients can be enrolled only after formal approval from all respective Ethics Committees.
Copy of the national Competent Health Authority and of the national IEC/IRB approvals will be transmitted from the Investigator to the Sponsor before starting recruitment.

14.2. Informed Consent

All interviews will be conducted in the native language of the patients by the study personnel. Consent forms in the local language will be provided to the parents or guardians for their review (see Appendix IC). The parents or guardians will be asked to sign (or thumb-print whenever the parents/guardians are illiterate) consent to participate in a research study. The informed consent will describe the purpose of the study, the procedures to be followed, and the risks and benefits of participation. If a parent or guardian is unable to read or write an impartial witness will take part in the informed consent discussion and will sign the consent form. Parents or guardians will be informed that participation in the study is completely voluntary and that they may withdraw their child from the study at any time without any negative consequences.

14.3. Confidentiality and Publication of Results

All study documents are provided by the investigators and his/her appointed staff. None of this material may be disclosed to any part not directly involved in the study without written permission from the Investigator. Publication policy will be addressed in a separate agreement.

14.4. Protocol Amendments

Clinical protocol amendments are alterations to a legal document (the clinical protocol) and have the same legal status and must pass through the appropriate steps before being implemented. In general, any change must be approved by the IEC prior to be effective. Administrative changes need only notification to the IEC without approval. Any subsequent amendments shall be made available with an new protocol with the amendments incorporated and on separate sheets and must pass through the approval process.

14.5. Insurance

A no-fault liability insurance will be taken by the Sponsor and will cover both trial sites.

14.6. Archiving

The relevant study documents are those documents which individually and collectively permit to assess the conduct of the trial, the quality of the data produced and the compliance with GCP standards and applicable regulatory requirements. The PI's file on site should at least contain all the essential documents as listed in the Sponsor's SOP "Set up and maintenance of the Investigator Trial File". A copy of all source data and CRF's must always be kept on site.
The PI is responsible for ensuring a secure and appropriate location for his investigator's file and any other trial related documentation present at site, as well as for ensuring that only site staff that is competent and delegated to work for the trial has got access to the files.
All the relevant study documentation present at all partners involved should be retained for a minimum of five years and according to the applicable National Legislation. The Sponsor should always be informed prior to destruction of the files.
After completion of the study, the IF will remain available for internal audits and/or inspections of regulatory authorities for a period of twenty years, unless differently requested by national authorities.

15. DATA SAFETY AND MONITORING BOARD

An independent Data Safety and Monitoring Board (DSMB) with at least 3 members (a clinician, possibly a pediatrician; a statistician and a malariologist/ epidemiologist), will be created before any field activity starts. The DSMB will be established for the purpose of providing independent advice on the quality of the data produced, the efficacy and safety of the treatments tested, so contributing to safeguarding the interests of the trial participants. More specifically the DSMB will:

- assess the quality of data, including completeness;
- monitor recruitment figures and losts to follow-up;
- monitor compliance with the protocol by participants and investigators;
- monitor evidence for treatment differences in the main efficacy and safety outcome measures and thus recommending action when/whether the main trial question has been answered;
- monitor evidence for treatment harm e.g. toxicity, SAEs, deaths;
- recommend whether the trial should continue to recruit or follow-up;
- recommend any major changes to the protocol, where necessary (e.g. changes to recruitment procedures, inclusion criteria, endpoints, data collection, etc);
- advise on and/or endorsing any major protocol modifications suggested by the Trial Steering Committee (e.g. changes to the inclusion criteria, endpoints, data collection, etc);
- monitor planned sample size assumptions;
- suggest additional data analyses;
- assess the impact and relevance of any external evidence provided;
- monitor compliance with previous DSMB recommendations;
- consider the ethical implications of any recommendations made by the DSMB.

In this respect, the DSMB will have the possibility to monitor the safety of the treatment on a continuous basis and possibly stop the study if any major safety concern appears. A DSMB charter, where the relevant terms of reference are clearly defined, will be signed by each member of the Committee.

16. ETHICAL ISSUES

The Investigator agrees to conduct the present study in full agreement with the principles of the "Declaration of Helsinki" and subsequent relevant amendments (see Appendix IV).
All research activities will be run in accordance with the standards and codes of conduct accepted by the International Conference on Harmonisation (ICH) guidelines.

AL and ASAQ have been successfully used for the treatment of falciparum malaria in Phase III studies and are both widely used to treat uncomplicated malaria in Africa. The combination are well tolerated. In RDCongo quinine + clindamycin is the second line treatment for malaria. Uganda has not yet adjust her treatment policy. The treatment is cheap, widely available and generally considered to be effective.

Blood will be collected for the haematology, biochemistry, blood slides, and later genotyping and other tests that the clinician may request for. The amount collected for haemotology and biochemistry will be 3 ml (venous) while for the other tests it will amount to a few drops collected by fingerprick. Residual plasma will be separated, stored

and transported at –70°C to be assayed by ELISA and FACS for malarial antibody quantification (described in independent protocol after the operational part of the study is finished).

The study will be presented for ethical clearance to all concerned Ethical commitees, as described above. Prior to the start of the project, the study will be explained to the local authorities of the communities involved. Written informed consent will be obtained from the guardians for all children before entering the study.

17. REFERENCES

See APPENDIX VIII

APPENDICES

APPENDIX I

Guidelines for Grading Patient Symptoms, signs and laboratory findings.

Table A. Guidelines for Grading Patient Symptoms.

	Grade 1<br>MILD	Grade 2<br>MODERATE	Grade 3<br>SEVERE	Grade 4<br>LIFE THREATENING
**Subjective fever in the past 24 h**	N/A	Present (Yes)	N/A	N/A
Weakness	Mild decrease in activity; For children – weak, but still playing	Moderate decrease in activity; For children – weak, and playing limited	Not participating in usual activities; For children – not playing	Prostration
**Muscle and/or joint aches***	Mild and/or localized complaints	Diffuse complaints	Objective weakness; function limited	N/A
**Headache***	Mild, no treatment required	Transient, moderate; treatment required	Severe, constant; requires narcotic therapy	Intractable; requires repeated narcotic therapy
**Anorexia**	Decreased appetite, but still taking solid food	Decreased appetite, avoiding solid food but taking liquids	Appetite very decreased; Refusing to breast feed, no solids or liquids taken (< 2 years ≤ 12 hr; > 2 years ≤ 24 hr)	Appetite very decreased; Refusing to breast feed, no solids or liquids taken (< 2 years > 12 hr; > 2 years > 24 hr)
**Nausea***	Mild, transient feeling of impending vomiting; maintains reasonable intake	Moderate and/or constant feeling of impending vomiting; intake decreased	Severe, constant feeling of impending emesis; intake decreased significantly	N/A
**Vomiting**	1 episode per day	2-3 episodes per day	Orthostatic hypotension or IV fluids required	Hypotensive shock or physiological consequences requiring IV fluid therapy
**Abdominal pain***	Mild (1-3 on a scale of 1 to 10)	Moderate (4-6 on a scale of 1 to 10)	Moderate to severe (≥ 7 on a scale of 1 to 10)	Severe – hospitalization for treatment
**Diarrhea**	Transient 3-4 loose stools/day	5-7 loose stools/day	Orthostatic hypotension or > 7 loose stools/day or IV fluids required	Hypotensive shock or physiological consequences requiring IV fluid therapy
**Cough**	Transient / intermittent	Persistent / constant	Uncontrolled	Cyanosis, stridor, severe shortness of breath
**Pruritis**	Transient pruritis	Pruritis that disturbs sleep	Severe, constant pruritis, sleep disturbed	N/A
**Tinnitus***	Mild, transient ringing or roaring sound	Moderate, persistent ringing or roaring sound	Severe ringing or roaring sound with associated hearing loss	N/A
**Behavioural changes**	Mild difficulty concentrating; mild confusion or agitation; activities of daily living	Moderate confusion or agitation; some limitation of activities of daily living; minimal	Severe confusion or agitation; Needs assistance for activities of daily living;	Toxic psychosis; hospitalization for treatment

	unaffected; no treatment	treatment	therapy required	
**"Flu" (viral URI)**	Mild nasal congestion, mild rhinorrhea	Moderate nasal congestion, moderate rhinorrhea	N/A	N/A
**Allergic reaction**	N/A	N/A	Urticaria	Severe urticaria anaphylaxis, angioedema
**Convulsion**	N/A	N/A	Localized or generalized seizure	Status epilepticus
*** Assess only in children ≥ 3 years of age. Answer N/A for younger children and those unable to answer.**				

Reference – Based on WHO Toxicity Grading Scale for Determining the Severity of Adverse Events

Table B. Guidelines for Physical Examination

**Dehydration**	Assess skin touch and turgor, mucous membranes, eyes, crying, fontanelle, pulse, urine output
**Jaundice**	Assess for yellowing of the sclera. Also evaluate the palpebral conjunctiva, lips, and skin.
**Chest**	Observe the rate, rhythm, depth, and effort of breathing. Check the patient's colour for cyanosis.<br><br>The maximum acceptable respiratory rate by age: < 2 months = 60, 2-12 months = 50, 1-5 years = 40, above 5 years = 30.<br><br>Inspect the neck for the position of the trachea, for supraclavicular retractions, and for contraction of the sternomastoid or other accessory muscles during inspiration.<br><br>Auscultate the anterior and posterior chest for normal breath sounds and any adventitious sounds (crackles or rales, wheezes, and rhonchi). *Crackles are intermittent, non-musical, fine or coarse sounds that may be due to abnormalities of the lungs (pneumonia, fibrosis, early congestive heart failure) or airways (bronchitis or bronchiectasis). Wheezes are high-pitched and result from narrowed airways. Rhonchi are relatively low-pitched and suggest secretions in large airways.*<br><br>If abnormalities are identified, evaluate for transmitted voice sounds. In addition, palpate the chest to assess for tactile fremitus, and percuss the chest to assess for areas of dullness. *Normal, air-filled lungs emit predominantly vesicular breath sounds, transmit voice sounds poorly with "ee" = "ee", and have no tactile fremitus. Airless lung, as in lobar pneumonia, emits bronchial breath sounds, transmits spoken words clearly with "ee" = "aay" (egophany), and has an increase in tactile fremitus.*
**Abdomen**	Inspection and auscultation of the abdomen. Listen for bowel sounds in the abdomen before palpating it. Palpate the abdomen in all 4 quadrants lightly and then deeply. Assess the size of the liver and spleen. To assess for peritoneal inflammation, look for localised and rebound tenderness, and voluntary or involuntary rigidity.
**Skin**	Inspect the skin for colour, turgor, moisture, and lesions. If lesions are present, note their location and distribution (diffuse or localised), arrangement (linear, clustered, annular, dermatomal), type (macules, papules, vesicles) and colour.
**Tablet test**	For children $\geq$ 9 months of age, ask the patient to pick a tablet (or equivalent object) up off a flat surface using the thumb and index finger of their dominant hand. *This tests for co-ordination of the upper extremity assessing the function of the motor system, cerebellar system, vestibular system (for coordinating eye and body movements) and the sensory system, for position sense. When testing small children, be aware that they will likely attempt to put the object into their mouth.*

Table C. Grading Physical Examination Findings

	**Grade 1**<br>**MILD**	**Grade 2**<br>**MODERATE**	**Grade 3**<br>**SEVERE**	**Grade 4**<br>**LIFE-THREATENING**
**Temperature * (tympanic)**	38..0-38.5°C	38.5-40.0°C	> 40.0°C	Sustained fever, equal or greater than 40.0°C for longer than 5 days
**Dehydration**	Less than 2 of the following:<br>Restless, irritable<br>Sunken eyes<br>Drinks eagerly, thirsty<br>Skin pinch goes back slowly	2 of the following:<br>Restless, irritable<br>Sunken eyes<br>Drinks eagerly, thirsty<br>Skin pinch goes back slowly	Two of the following:<br>Lethargic or unconscious<br>Sunken eyes<br>Not able to drink or drinking poorly<br>Skin pinch goes back very poorly	Two of the following + shock:<br>Lethargic or unconscious<br>Sunken eyes<br>Not able to drink or drinking poorly<br>Skin pinch goes back very poorly
**Jaundice**	Slight yellowing of sclera and conjunctiva	Moderate yellowing of sclera and conjunctiva, yellowing of mucous membranes	Severe yellowing of sclera and conjunctiva, yellowing of skin	N/A
**Chest**	Mildly increased RR (for age, temperature), transient or localised adventitious sounds	Moderately increased RR, diffuse or persistent adventitious sounds	Rapid RR (< 2 months > 60, 2-12 months > 50, 1-5 years > 40, adults > 30)* nasal flaring, retractions	Cyanosis
**Abdomen**	Normal bowel sounds, mild localised tenderness, and/or liver palpable 2-4 cm below the right costal margin (RCM), and/or spleen palpable, and/or umbilical hernia present	Normal or mildly abnormal bowel sounds, moderate or diffuse tenderness; and/or mild to moderately enlarged liver (4-6 cm below the RCM) and/or spleen palpable up to half-way between umbilicus and symphysis pubis	Severely abnormal bowel sounds, severe tenderness to palpation. Evidence of peritoneal irritation and/or significant enlargement of liver (> 6 cm below the RCM) and/or spleen palpable beyond half-way between umbilicus and symphysis pubis	Absent bowel sounds. Involuntary rigidity
**Skin†**	Localised rash, erythema, or pruritis	Diffuse, maculopapular rash, dry desquamation	Vesiculation, moist desquamation, or ulceration	Exfoliative dermatitis, mucous membrane involvement or erythema multiforme or suspected Stevens-Johnson or necrosis requiring surgery

	**Grade 1 MILD**	**Grade 2 MODERATE**	**Grade 3 SEVERE**	**Grade 4 LIFE-THREATENING**
**Hearing**	*< 4 years: N/A*<br>≥ 4 years: Decreased hearing in one ear	*< 4 years: N/A*<br>≥ 4 years: Decreased hearing in both ears or severe impairment in one ear	*< 4 years: Any evidence of hearing impairment*<br>≥ 4 years: Severe impairment in both ears	N/A
**Tablet test**	Difficulty grasping tablet but able to pick up	Unable to pick up tablet without dropping	Unable to grasp tablet	N/A
**Clinical symptoms / sign *(not otherwise specified)***	No treatment required; monitor condition	Treatment required	Requires treatment and possible hospitalisation	Requires active medical intervention, hospitalisation, or hospice care

Reference – The Harriet Lane Handbook, 15th edition, 2000
† Reference – WHO Toxicity Grading Scale for Determining the Severity of Adverse Events

TABLE D. Guidelines for Grading of Laboratory results

	**Grade 1 MILD**	**Grade 2 MODERATE**	**Grade 3 SEVERE**	**Grade 4 LIFE-THREATENING**
**Absolute neutrophil count* *(/mm³)***	750-1200	400-749	250-399	< 250
**Hemoglobin** (*g/dL*)	9.0 – 9.9	7.0 – 8.9	5.0 – 6.9	< 5.0
**Platelets *(/mm³)****	N/A	50,000-75,000	25,000-49,999	< 25,000
**ALT *(U/L)*****	1.1-4.9 x ULN (50 – 224)	5.0-9.9 x ULN (225 – 449)	10.0-15.0 x ULN (450 – 675)	> 15.0 x ULN (> 675)
**Bilirubin (*mg/dL)****	1.1-1.9 x ULN (1.3 - 2.3)	2.0-2.9 x ULN (2.4 - 3.5)	3.0-7.5 x ULN (3.6 - 9.0)	> 7.5 x ULN (> 9.0)
**Creatinine *(mg/dl))** Age < 2 years**	0.6-0.8	0.9-1.1	1.2-1.5	> 1.5
**Creatinine *(mg/dl))** Age ≥ 2 years**	0.7-1.0	1.1-1.6	1.7-2.0	> 2.0
**Laboratory values** *(not otherwise specified)*	Abnormal but requiring no immediate intervention; follow	Sufficiently abnormal to require evaluation as to causality and perhaps mild therapeutic intervention	Sufficiently severe to require evaluation and treatment	Life-threatening severity; requires immediate evaluation, treatment, and usually hospitalization

*Reference – DMID Pediatric Toxicity Tables, May 2001
** Reference – DAIDS Pediatric guidelines December 2004

APPENDIX II*

Table A: Coartem® dose based on weight will be given daily. Tablets containing 20 mg of Artemether and 120 mg of Lumefantrine. Each dose to be taken with high-fat food or drinks (for example milk.)

Weight in kg	Number of tablet per dose
Age<br>5 to < 15 kg	1 tablet per dose
15 to < 25 kg	2 tablets per dose
25 to < 35 kg	3 tablets per dose

Table B: Co-arsucam® based on age will be given daily as described in table below

Age<br>(Weight in Kg)	Dose	Treatment duration
2 to 11 months<br>(= 4,5 to < 9kg)	1 tablet (25 mg Artesunate/ 67,5 mg Amodiaquine)	3 days
1 to 5 years<br>(= 9 kg to < 18 kg)	1 tablet (50 mg Artesunate/ 135 mg Amodiaquine)	3 days
6 to 13 years<br>(= 18 kg to < 36 kg)	1 tablet (100 mg Artesunate/ 270 mg Amodiaquine)	3 days

Table C : Quinimax® tablet 125mg based on weight will be given daily as described in table below in combination with clindamycin (10 mg/kg twice daily)

Weight in kg	Number of tablet per dose	Treatment duration
9 to < 11 kg	½ tablets per dose	7 days
12 to < 19 kg	1 tablets per dose	7 days
20 to < 27 kg	1½ tablets per dose	7 days
28 to < 35 kg	2 tablets per dose	7 days

APPENDIX III

LIST of drugs having antiplasmodium activities

Chloroquine.
Quinine, quinidine, cinchonine, cinchonidine.
Halofantrine.
Luméfantrine
Dérivés de l'Artemisinine
Proguanil.
Sulfalène.
Pyriméthamine.
Sulfadoxine.
Sulfisoxazole.
Sulfadiazine.
Sulfasalazine.
primaquine.
atovaquone.
Doxycycline et autres cyclines.
Azythromycine.
Erythromycine.
Pentamidine.
Clindamycine.
Rifampine.
Dapsone.
Triméthoprime sulfaméthoxazole.

APPENDIX IV

WORLD MEDICAL ASSOCIATION DECLARATION OF HELSINKI
Ethical Principles for Medical Research Involving Human Subjects

Adopted by the 18th WMA General Assembly, Helsinki, Finland, June 1964, and amended by the:
29th WMA General Assembly, Tokyo, Japan, October 1975
35th WMA General Assembly, Venice, Italy, October 1983
41st WMA General Assembly, Hong Kong, September 1989
48th WMA General Assembly, Somerset West, Republic of South Africa, October 1996
52nd WMA General Assembly, Edinburgh, Scotland, October 2000
53th WMA General Assembly, Washington 2002 (Note of Clarification on paragraph 29 added)
55th WMA General Assembly, Tokyo 2004 (Note of Clarification on Paragraph 30 added)
59th WMA General Assembly, Seoul, October 2008

A. INTRODUCTION

1. The World Medical Association (WMA) has developed the Declaration of Helsinki as statement of ethical principles for medical research involving human subjects**,** including research on identifiable human material and data.

   The Declaration is intended to be read as a whole and each of its constituent paragraphs should not be applied without consideration of all other relevant paragraphs.

2. Although the Declaration is addressed primarily to physicians, the WMA encourages other participants in medical research involving human subjects to adopt these principles.
3. It is the duty of the physician to promote and safeguard the health of patients, including those who are involved in medical research. The physician's knowledge and conscience are dedicated to the fulfilment of this duty.
4. The Declaration of Geneva of the WMA binds the physician with the words, “The health of my patient will be my first consideration,” and the International Code of Medical Ethics declares that, “A physician shall act in the patient's best interest when providing medical care.”
5. Medical progress is based on research that ultimately must include studies involving human subjects. Populations that are underrepresented in medical research should be provided appropriate access to participation in research.
6. In medical research involving human subjects, the well-being of the individual research subject must take precedence over all other interests.
7. The primary purpose of medical research involving human subjects is to understand the causes, development and effects of diseases and improve preventive, diagnostic and therapeutic interventions (methods, procedures and treatments). Even the best current interventions must be evaluated continually through research for their safety, effectiveness, efficiency, accessibility and quality.
8. In medical practice and in medical research, most interventions involve risks and burdens.
9. Medical research is subject to ethical standards that promote respect for all human subjects and protect their health and rights. Some research populations are particularly vulnerable and need special protection. These include those who cannot give or refuse consent for themselves and those who may be vulnerable to coercion or undue influence.
10. Physicians should consider the ethical, legal and regulatory norms and standards for research involving human subjects in their own countries as well as applicable international norms and standards. No national or international ethical, legal or regulatory requirement should reduce or eliminate any of the protections for research subjects set forth in this Declaration.

B. PRINCIPLES FOR ALL MEDICAL RESEARCH

11. It is the duty of physicians who participate in medical research to protect the life, health, dignity, integrity, right to self-determination, privacy, and confidentiality of personal information of research subjects.

12. Medical research involving human subjects must conform to generally accepted scientific principles, be based on a thorough knowledge of the scientific literature, other relevant sources of information, and adequate laboratory and, as appropriate, animal experimentation. The welfare of animals used for research must be respected.
13. Appropriate caution must be exercised in the conduct of medical research that may harm the environment.
14. The design and performance of each research study involving human subjects must be clearly described in a research protocol. The protocol should contain a statement of the ethical considerations involved and should indicate how the principles in this Declaration have been addressed. The protocol should include information regarding funding, sponsors, institutional affiliations, other potential conflicts of interest, incentives for subjects and provisions for treating and/or compensating subjects who are harmed as a consequence of participation in the research study. The protocol should describe arrangements for post-study access by study subjects to interventions identified as beneficial in the study or access to other appropriate care or benefits.
15. The research protocol must be submitted for consideration, comment, guidance and approval to a research ethics committee before the study begins. This committee must be independent of the researcher, the sponsor and any other undue influence. It must take into consideration the laws and regulations of the country or countries in which the research is to be performed as well as applicable international norms and standards but these must not be allowed to reduce or eliminate any of the protections for research subjects set forth in this Declaration. The committee must have the right to monitor ongoing studies. The researcher must provide monitoring information to the committee, especially information about any serious adverse events. No change to the protocol may be made without consideration and approval by the committee.
16. Medical research involving human subjects must be conducted only by individuals with the appropriate scientific training and qualifications. Research on patients or healthy volunteers requires the supervision of a competent and appropriately qualified physician or other health care professional. The responsibility for the protection of research subjects must always rest with the physician or other health care professional and never the research subjects, even though they have given consent.
17. Medical research involving a disadvantaged or vulnerable population or community is only justified if the research is responsive to the health needs and priorities of this population or community and if there is a reasonable likelihood that this population or community stands to benefit from the results of the research.
18. Every medical research study involving human subjects must be preceded by careful assessment of predictable risks and burdens to the individuals and communities involved in the research in comparison with foreseeable benefits to them and to other individuals or communities affected by the condition under investigation.
19. Every clinical trial must be registered in a publicly accessible database before recruitment of the first subject.
20. Physicians may not participate in a research study involving human subjects unless they are confident that the risks involved have been adequately assessed and can be satisfactorily managed. Physicians must immediately stop a study when the risks are found to outweigh the potential benefits or when there is conclusive proof of positive and beneficial results.
21. Medical research involving human subjects may only be conducted if the importance of the objective outweighs the inherent risks and burdens to the research subjects.
22. Participation by competent individuals as subjects in medical research must be voluntary. Although it may be appropriate to consult family members or community leaders, no competent individual may be enrolled in a research study unless he or she freely agrees.
23. Every precaution must be taken to protect the privacy of research subjects and the confidentiality of their personal information and to minimize the impact of the study on their physical, mental and social integrity.
24. In medical research involving competent human subjects, each potential subject must be adequately informed of the aims, methods, sources of funding, any possible conflicts of interest, institutional affiliations of the researcher, the anticipated benefits and potential risks of the study and the discomfort it may entail, and any other relevant aspects of the study. The potential subject must be informed of the right to refuse to participate in the study or to withdraw consent to participate at any time without reprisal. Special attention should be given to the specific information needs of individual potential subjects as well as to the methods used to deliver the information. After ensuring that the potential subject has understood the information, the physician or another appropriately qualified individual must then seek the potential subject's freely-given informed consent, preferably in writing. If the consent cannot be expressed in writing, the non-written consent must be formally documented and witnessed.

25. For medical research using identifiable human material or data, physicians must normally seek consent for the collection, analysis, storage and/or reuse. There may be situations where consent would be impossible or impractical to obtain for such research or would pose a threat to the validity of the research. In such situations the research may be done only after consideration and approval of a research ethics committee.

26. When seeking informed consent for participation in a research study the physician should be particularly cautious if the potential subject is in a dependent relationship with the physician or may consent under duress. In such situations the informed consent should be sought by an appropriately qualified individual who is completely independent of this relationship.

27. For a potential research subject who is incompetent, the physician must seek informed consent from the legally authorized representative. These individuals must not be included in a research study that has no likelihood of benefit for them unless it is intended to promote the health of the population represented by the potential subject, the research cannot instead be performed with competent persons, and the research entails only minimal risk and minimal burden.

28. When a potential research subject who is deemed incompetent is able to give assent to decisions about participation in research, the physician must seek that assent in addition to the consent of the legally authorized representative. The potential subject's dissent should be respected.

29. Research involving subjects who are physically or mentally incapable of giving consent, for example, unconscious patients, may be done only if the physical or mental condition that prevents giving informed consent is a necessary characteristic of the research population. In such circumstances the physician should seek informed consent from the legally authorized representative. If no such representative is available and if the research cannot be delayed, the study may proceed without informed consent provided that the specific reasons for involving subjects with a condition that renders them unable to give informed consent have been stated in the research protocol and the study has been approved by a research ethics committee. Consent to remain in the research should be obtained as soon as possible from the subject or a legally authorized representative.

30. Authors, editors and publishers all have ethical obligations with regard to the publication of the results of research. Authors have a duty to make publicly available the results of their research on human subjects and are accountable for the completeness and accuracy of their reports. They should adhere to accepted guidelines for ethical reporting. Negative and inconclusive as well as positive results should be published or otherwise made publicly available. Sources of funding, institutional affiliations and conflicts of interest should be declared in the publication. Reports of research not in accordance with the principles of this Declaration should not be accepted for publication.

C. ADDITIONAL PRINCIPLES FOR MEDICAL RESEARCH COMBINED WITH MEDICAL CARE

31. The physician may combine medical research with medical care only to the extent that the research is justified by its potential preventive, diagnostic or therapeutic value and if the physician has good reason to believe that participation in the research study will not adversely affect the health of the patients who serve as research subjects.

32. The benefits, risks, burdens and effectiveness of a new intervention must be tested against those of the best current proven intervention, except in the following circumstances:

    • The use of placebo, or no treatment, is acceptable in studies where no current proven intervention exists; or

    • Where for compelling and scientifically sound methodological reasons the use of placebo is necessary to determine the efficacy or safety of an intervention and the patients who receive placebo or no treatment will not be subject to any risk of serious or irreversible harm. Extreme care must be taken to avoid abuse of this option.

33. At the conclusion of the study, patients entered into the study are entitled to be informed about the outcome of the study and to share any benefits that result from it, for example, access to interventions identified as beneficial in the study or to other appropriate care or benefits.

34. The physician must fully inform the patient which aspects of the care are related to the research. The refusal of a patient to participate in a study or the patient's decision to withdraw from the study must never interfere with the patient-physician relationship.

35. In the treatment of a patient where proven interventions do not exist or have been ineffective, the physician, after seeking expert advice, with informed consent from the patient or a legally authorized representative, may use an unproven intervention if in the physician's judgement it offers hope of saving life, re-establishing health or

alleviating suffering. Where possible, this intervention should be made the object of research, designed to evaluate its safety and efficacy. In all cases, new information should be recorded and, where appropriate, made publicly available.

APPENDIX V

Criteria for Severe Malaria/Danger Signs

<u>Severe Malaria</u>

- Unarousable coma *(if after convulsion, > 30 min)*
- Repeated convulsions *(> 2 within 24 h)*
- Severe anaemia *(Hb < 5.0 g/dL)*
- Respiratory distress *(laboured breathing at rest)*

<u>Danger Signs</u>

- Recent convulsions *(>1 within 24 h)*
- Altered consciousness *(confusion)*
- Lethargy
- Unable to drink or breast feed
- Vomiting everything
- Unable to stand/sit due to weakness

APPENDIX VI. PARTICIPANT SELECTION AND ENROLLMENT

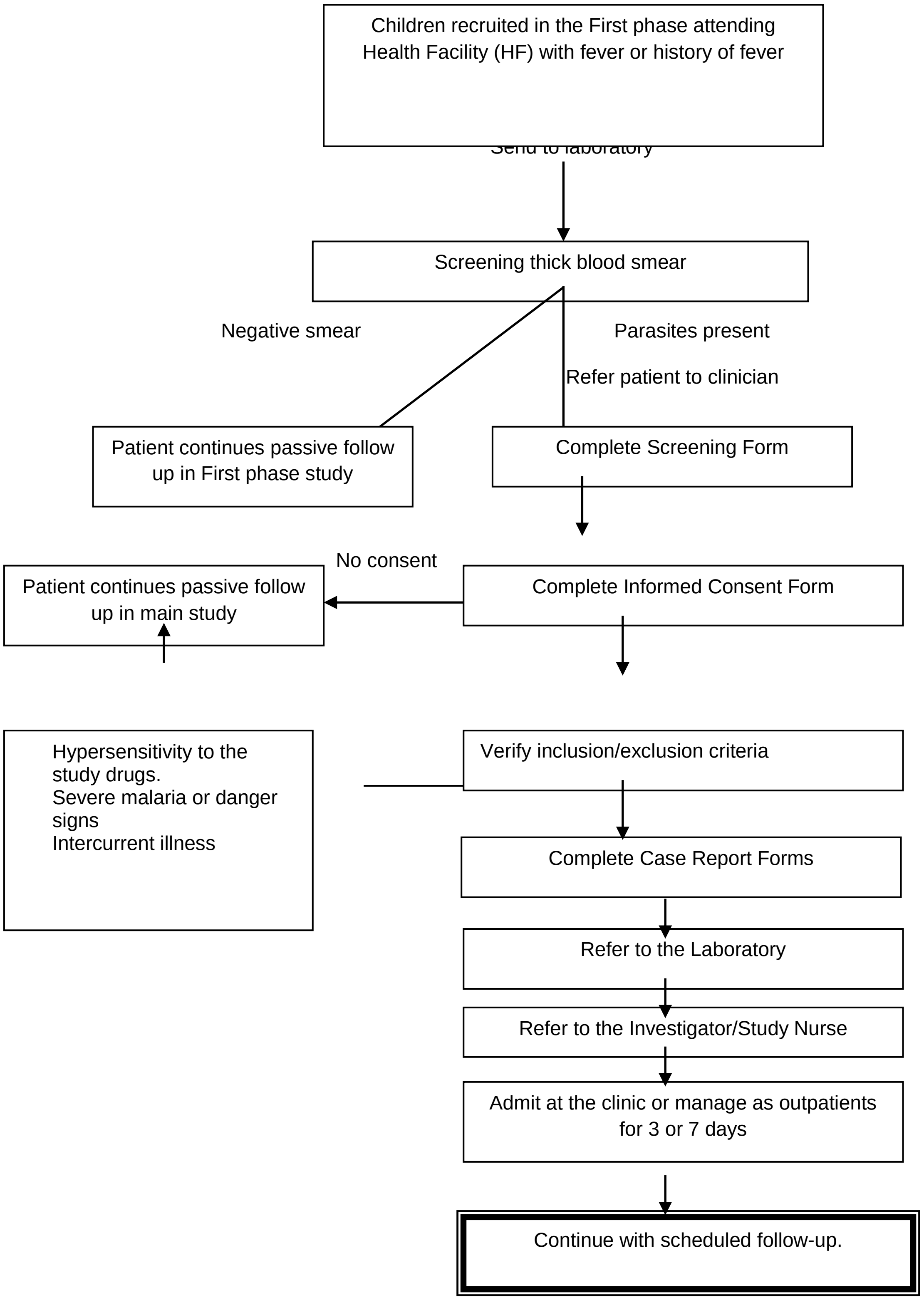

APPENDIX VII. CRITICAL STEPS.

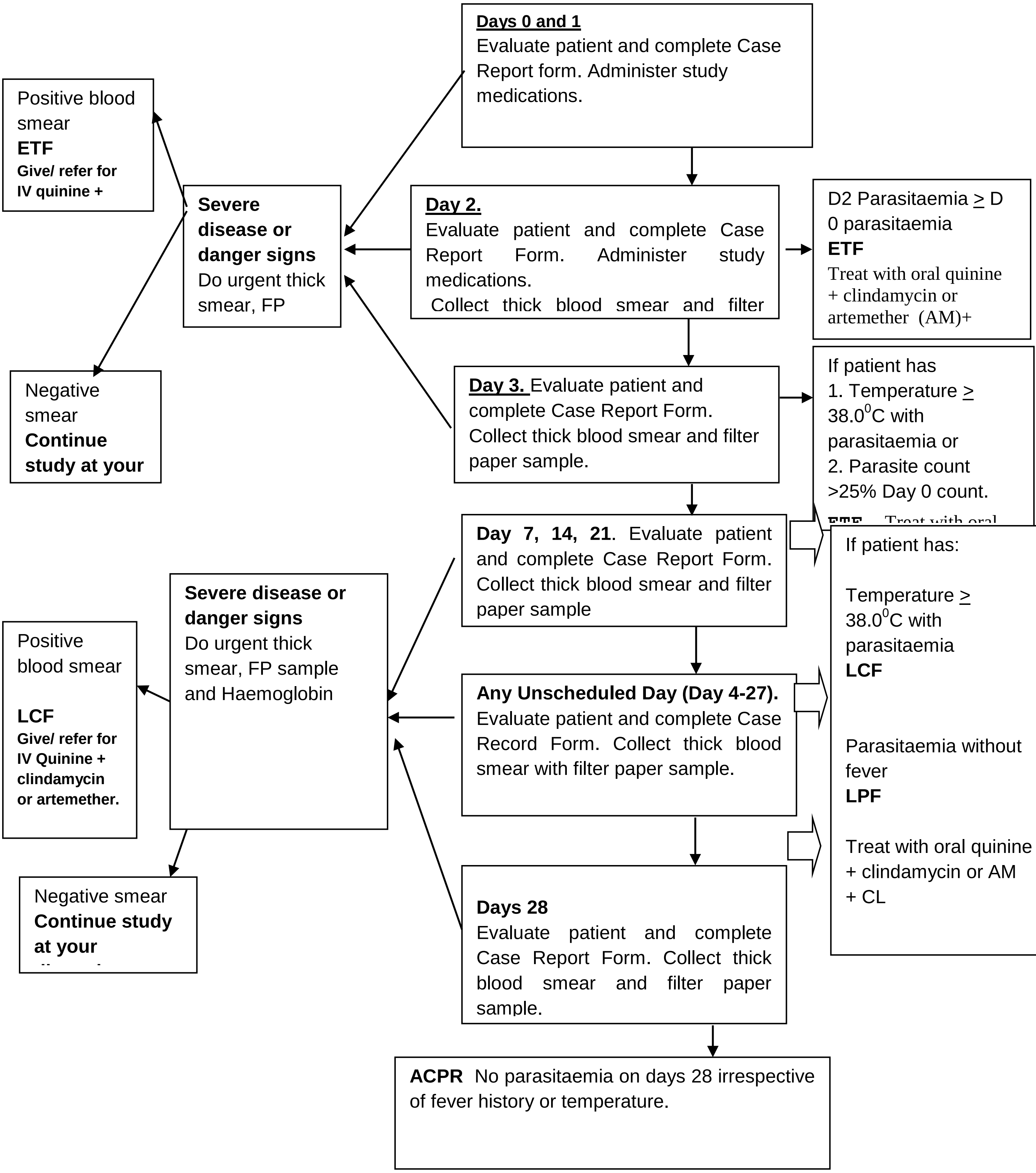

APPENDIX VIII..

Reference List

(1) Hay S, Guerra C, Tatem A, Noor A, Snow R. The global distribution and population at risk of malaria: past, present, and future. Lancet Infect Dis 2004;4:327-36.

(2) White NJ, Olliaro P. Artemisinin and derivatives in the treatment of uncomplicated malaria. Med Trop (Mars) 1998;58(3 Suppl):54-6.

(3) Ndiaye JL, Randrianarivelojosia M, Sagara I, et al. Randomized, multicentre assessment of the efficacy and safety of ASAQ--a fixed-dose artesunate-amodiaquine combination therapy in the treatment of uncomplicated Plasmodium falciparum malaria. Malar J 2009;8:125.

(4) Swarthout TD, van dB, IV, Kayembe G, Montgomery J, Pota H, Roper C. Artesunate + amodiaquine and artesunate + sulphadoxine-pyrimethamine for treatment of uncomplicated malaria in Democratic Republic of Congo: a clinical trial with determination of sulphadoxine and pyrimethamine-resistant haplotypes. Trop Med Int Health 2006 Oct;11(10):1503-11.

(5) Sirima SB, Tiono AB, Gansane A, et al. The efficacy and safety of a new fixed-dose combination of amodiaquine and artesunate in young African children with acute uncomplicated Plasmodium falciparum. Malar J 2009;8:48.

(6) Zwang J, Olliaro P, Barennes H, et al. Efficacy of artesunate-amodiaquine for treating uncomplicated falciparum malaria in sub-Saharan Africa: a multi-centre analysis. Malar J 2009;8:203.

(7) Byakika-Kibwika P, Lamorde M, Mayanja-Kizza H, Merry C, Colebunders B, van Geertruyden JP. Update on the efficacy, effectiveness and safety of artemether-lumefantrine combination therapy for treatment of uncomplicated malaria. Ther Clin Risk Manag 2010;6:11-20.

(8) de Vries PJ, Bich NN, Van Thien H, et al. Combinations of artemisinin and quinine for uncomplicated falciparum malaria: efficacy and pharmacodynamics. Antimicrob Agents Chemother 2000 May;44(5):1302-8.

(9) Yeka A, Achan J, D'Alessandro U, Talisuna AO. Quinine monotherapy for treating uncomplicated malaria in the era of artemisinin-based combination therapy: an appropriate public health policy? Lancet Infect Dis 2009 Jul;9(7):448-52.

(10) Bloland P. Drug resistance in malaria. A background document for the WHO global strategy for containment of antimicrobial resistance. WHO/ CDS/ CSR/ DRS/ 2001 4 2001

(11) Fungladda W, Honrado ER, Thimasarn K, et al. Compliance with artesunate and quinine + tetracycline treatment of uncomplicated falciparum malaria in Thailand. Bull World Health Organ 1998;76 Suppl 1:59-66.

(12) Achan J, Tibenderana JK, Kyabayinze D, et al. Effectiveness of quinine versus artemether-lumefantrine for treating uncomplicated falciparum malaria in Ugandan children: randomised trial. BMJ 2009;339:b2763.

(13) Bassat Q, Mulenga M, Tinto H, et al. Dihydroartemisinin-piperaquine and artemether-lumefantrine for treating uncomplicated malaria in African children: a randomised, non-inferiority trial. PLoS One 2009;4(11):e7871.

(14) McNutt LA, Wu C, Xue X, Hafner JP. Estimating the relative risk in cohort studies and clinical trials of common outcomes. Am J Epidemiol 2003 May 15;157(10):940-3.

(15) World Health Organization. Guidelines for Treatment of malaria second edition. Geneva: WHO, 2010. [http://www.who.int/malaria/docs/TreatmentGuidelines2010.pdf] (consulted on 30 November 2010)

(16) Bonnet M, Broek I, van Herp M, Urrutia PP, van Overmeir C, Kyomuhendo J, Ndosimao CN, Ashley E, Guthmann JP. Varying efficacy of artesunate+amodiaquine and artesunate+sulphadoxine-pyrimethamine for the treatment of uncomplicated falciparum malaria in the Democratic Republic of Congo: a report of two in-vivo studies. *Malar J* 2009;8:192.
